# Shared in planta population and transcriptomic features of nonpathogenic members of endophytic phyllosphere microbiota

**DOI:** 10.1073/pnas.2114460119

**Published:** 2022-03-28

**Authors:** André C. Velásquez, José C. Huguet-Tapia, Sheng Yang He

**Affiliations:** ^a^Howard Hughes Medical Institute, Duke University, Durham, NC 27708;; ^b^Department of Plant Pathology, University of Florida, Gainesville, FL 32611;; ^c^Department of Biology, Duke University, Durham, NC 27708

**Keywords:** plant microbiota, population stasis, bacterial transcriptome, *Pseudomonas syringae*, plant immunity

## Abstract

Plants and animals are in constant association with a variety of microbes. Although much is known about how pathogenic and symbiotic microbes interact with plants, less is known about the population dynamics, adaptive traits, and transcriptional features of the vast number of microbes that make up the bulk of the plant microbiota. The majority of microbiota taxa are either commensal, natural mutants of pathogens, or pathogens that encounter strong immune responses due to plant recognition of pathogen effectors. How these “nonpathogenic” microbes interact with plants is poorly understood, especially during long-term, steady-state interactions, which are more reflective of plant–microbiota interactions in nature. In this study, we embarked upon long-term population and in planta transcriptomic studies of commensal endophytic bacteria and compared them to nonpathogenic or effector-triggered immunity-inducing strains of the bacterial pathogen *Pseudomonas syringae*. Our results led to the discovery of multiplication–death equilibrium as a common basis for the shared long-term static population densities of these bacteria. A comprehensive in planta transcriptomic analysis using multiple time points after inoculation revealed a striking similarity between the transcriptomic features of nonpathogenic *P. syringae* to that of bacteria in stationary phase in vitro, a metabolically active physiological state in which the production of adaptive secondary metabolites and stress responses are induced. We propose that the long-term population and transcriptomic features of nonpathogenic bacteria captured in this study likely reflect the physiological steady state encountered by the bulk of endophytic microbiota—excluding virulent pathogens—in their life-long interactions with plants in nature.

## Significance

Plants evolved in an environment colonized by a vast number of microbes, which collectively constitute the plant microbiota. The majority of microbiota taxa are nonpathogenic and may be beneficial to plants under certain ecological or environmental conditions. We conducted experiments to understand the features of long-term interactions of nonpathogenic microbiota members with plants. We found that a multiplication–death equilibrium explained the shared long-term static populations of nonpathogenic bacteria and that in planta bacterial transcriptomic signatures were characteristic of the stationary phase, a physiological state in which stress protection responses are induced. These results may have significant implications in understanding the bulk of “nonpathogenic” plant–microbiota interactions that occur in agricultural and natural ecosystems.

Plants are exposed to a multitude of microbes during their life cycles. Interactions of plants with microbes may range from causing no observable effect (commensal interactions) to forming intricate symbiotic relationships with specialized plant organs for nutrient acquisition (1). Of the myriad extant microbes, only very few are capable of causing disease in a particular plant host (2). Due to their great impact on crop production and natural ecosystems, symbiotic and pathogenic microbes have received great attention in the past. By contrast, insight into the lives of the vast number of commensal microbes is lagging.

Of commensal bacteria that live on the phyllosphere (above-ground parts) of plants, most live on the surface as epiphytes, probably because the plant interior (including the apoplast) exerts a strong selection pressure on the type of microbes that can grow and multiply. Not only are bacterial epiphytes more than 100 times more abundant than endophytes, but also the microbiota composition is different in *Arabidopsis thaliana* (3). Moreover, commensal bacterial densities are generally low; in *Arabidopsis* the number of culturable endophytes appears to be fewer than 10^4^ colony-forming units (CFU) cm^−2^ (3, 4). By contrast, the population density of pathogenic endophytic bacteria, such as *Pseudomonas syringae*, can increase to almost the carrying capacity of the plant (approaching 10^8^ CFU cm^−2^) (5), unless the plant mounts effector-triggered immunity (ETI) upon recognition of virulence effector proteins by nucleotide-binding and leucine-rich repeat (NLR) proteins (6, 7). Interestingly, in the absence of virulence-promoting effectors and toxins, nonpathogenic mutants of *P. syringae*, such as the *ΔhrcCΔCFA* mutant (defective in type III secretion and coronatine production), are unable to multiply to high levels, resembling commensal bacteria that normally reside in the leaf apoplast (8).

The transcriptomes of phyllosphere-inhabiting pathogenic bacteria have been analyzed in the past few years to obtain clues into the processes that influence plant colonization. A microarray-based approach revealed differences in gene expression between epiphytic and apoplastic pathogenic populations of *P. syringae*, from which it was inferred that bacteria residing in the plant apoplast experience a more taxing osmotic stress than those that live on the surface of the leaves (at 48 to 72 h postinoculation [hpi]) (9). A more recent transcriptomics study focusing on the early responses of *P. syringae* (at 6 h after inoculation) showed a strong correlation between bacterial genes responsive to the plant immune system and future bacterial population densities at 48 h postinoculation (10). It should be pointed out that this early response captures transcriptomic changes during the transition of bacteria from nutrient-rich artificial media to the plant environment, a condition that may not be prevalent in nature. To date, the nature of long-term bacterial population homeostasis and associated transcriptomic dynamics of commensal endophytic phyllosphere bacteria has not been evaluated, leaving a significant gap in the understanding of population and molecular features that are important for long-term adaptation and survival of commensal microbiota to the apoplastic environment of the phyllosphere.

In this study, we conducted a detailed analysis of the population dynamics of two common commensal bacterial endophytes as well as a commensal-simulating mutant and an effector-triggered immunity-inducing strain of *P. syringae* pv. *tomato* DC3000 in *Arabidopsis* leaves. We then used multitime-point transcriptomic analysis to infer the biological processes important for long-term nonpathogenic commensal lifestyle in the leaf apoplast. Our results point to a previously unrecognized stationary-phase–like lifestyle for phyllosphere-inhabiting endophytic bacteria, which is likely reflective of what these bacteria experience during their steady-state interactions with plant hosts in nature. We provide multiple lines of evidence that this population stasis, also observed for ETI-inducing bacteria, was caused by an equilibrium in multiplication and death of phyllosphere-inhabiting endophytes. This finding has significant implications in understanding most plant–commensal endophytic microbiota interactions in nature and in guiding the application of endophytic commensal microbiota in agricultural and natural ecosystem settings.

## Results

### Static Long-Term Population Densities of Nonpathogenic Members of Endophytic Phyllosphere Microbiota.

**Fig. 1.**
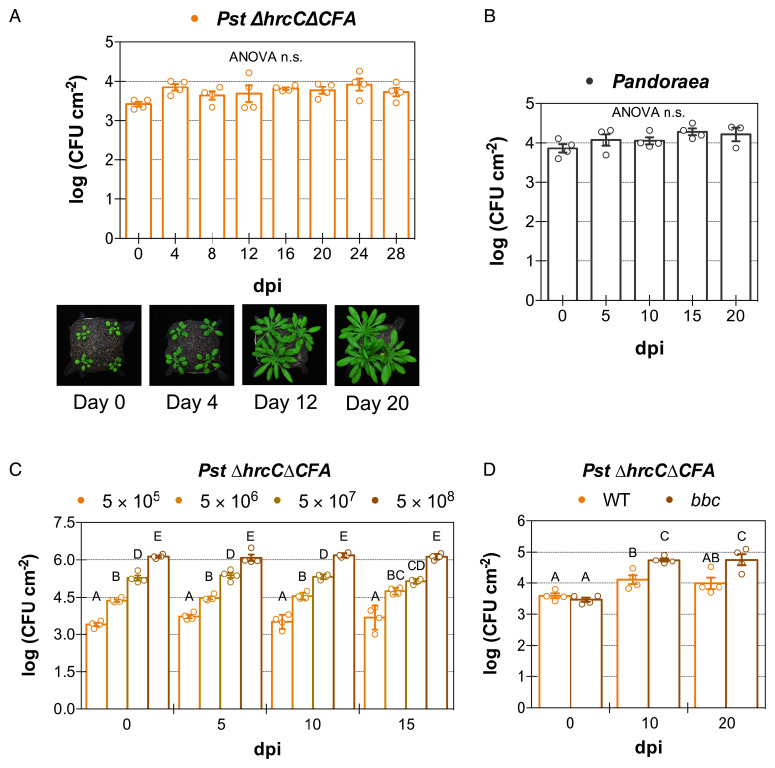
Nonpathogenic phyllosphere bacteria experience bacterial population stasis in planta. (*A*) Bacterial population density of nonpathogenic *P. syringae* pv. *tomato* (*Pst*) *ΔhrcCΔCFA* over the course of 28 d after infection of *Arabidopsis* accession Col-0 plants. Photographs of representative infected plants are shown beneath the graph. (*B*) Bacterial population density of endophytic microbiota strain *Pandoraea* sp. Col-0-28 over the course of 20 d in Col-0 plants. (*C*) Bacterial population density in Col-0 plants after infiltration with different inoculum concentrations of *Pst ΔhrcCΔCFA* (in CFU mL^−1^) remains static for over 2 wk. (*D*) Bacterial population density in Col-0 and PTI-compromised triple mutant *bak1 bkk1 cerk1* (*bbc*) of *Pst ΔhrcCΔCFA* (inoculum 5 × 10^5^ CFU mL^−1^) over the course of 20 d. Open circles show individual biological repetitions; error bars indicate the SEM. Different letters indicate different means (Tukey’s honestly significant difference test; *P* < 0.05). n.s., not significant.

Most previous laboratory studies have examined short-term (often within days of inoculation) population dynamics of nonpathogenic endophytic phyllosphere bacteria, which is only relevant during the initial bacterial plant colonization. Very few studies have evaluated the population dynamics of nonpathogenic endophytic bacteria over the longer time period that is likely characteristic of plant–microbiota interactions in nature (11). We first tested the long-term population dynamics of a disarmed (no longer pathogenic) strain of *P. syringae* pv. *tomato* (*Pst*) DC3000, *ΔhrcCΔCFA* (8), which should simulate the experience of commensal bacteria in nature, in *Arabidopsis* leaves over the course of 28 d. As shown in Fig. 1*A*, the bacterial population densities remained unchanged over the course of 4 wk. Next, we wanted to know whether the population stasis phenomenon was widespread and whether it also applied to commensal leaf microbiota strains. We inoculated three bacterial strains that had been previously isolated from *Arabidopsis* leaves: the β-Proteobacteria *Achromobacter xylosoxidans* Col-0-50 and *Pandoraea* sp. Col-0-28 (3), and the gram-positive Actinobacteria *Rhodococcus* sp. 964 (12) into *Arabidopsis* plants. These commensal microbiota strains were chosen because they did not cause any disease-like symptoms when inoculated into leaves even at very high population densities (3, 12). Similar to *Pst ΔhrcCΔCFA*, after 3 wk, population densities did not change for either Proteobacteria strain (Fig. 1*B* and *SI Appendix*, Fig. S1*A*), while there was a very small initial increase in growth for *Rhodococcus* (*SI Appendix*, Fig. S1*A*; this initial increase in growth was not always consistent, see *SI Appendix*, Figs. S1*D* and S2*D*).

To rule out the possibility that the population stasis phenomenon is observed only at population densities at which the carrying capacity of the apoplast has been reached, we inoculated *Pst ΔhrcCΔCFA* into *Arabidopsis* leaves at five different initial population densities (covering four orders of magnitude). Interestingly, irrespective of the bacterial inoculum, the population densities did not change over 2 wk, except for a slight increase early in the colonization in some experiments (Fig. 1*C* and *SI Appendix*, Fig. S1*B*). At the highest initial inoculum, the growth of inoculated leaves was arrested and an earlier onset-senescence phenotype was observed, in accordance with the well-known dichotomy between growth and defense (*SI Appendix*, Fig. S1*C*) (13). This result suggests that the growth–defense tradeoffs might not occur for plant–commensal interactions at the low natural endophyte population density that exists in nature. Additionally, the lack of differences in the overall population stasis phenotype at different initial population densities suggests that a lack of resources cannot explain why commensal phyllosphere bacteria are unable to multiply to achieve high population densities, as populations of up to 10^6^ CFU cm^−2^ were able to be maintained inside leaves.

A previous study showed that genetic disruption of pattern-triggered immunity (PTI) signaling alone does not allow a dramatic increase in nonpathogenic bacterial population density in planta, at least not within a few days after inoculation (less than a 10-fold increase) (4). We reasoned, however, that perhaps evaluating population densities over the course of weeks rather than days would show a continuous increase in bacterial population densities in PTI-compromised mutant plants. However, we still found static bacterial populations in a PTI mutant deficient in three PTI-associated coreceptors (*bak1 bkk1 cerk1*, involved in the recognition of multiple microbe-associated molecular patterns [MAMPs]) (4) almost 3 wk after inoculation (Fig. 1*D* and *SI Appendix*, Fig. S1*D*). Even though population densities of *Pst ΔhrcCΔCFA* and *Rhodococcus* were larger in the PTI triple mutant than in the wild type, these populations did not continue to increase over time and remained static after an initial growth (Fig. 1*D* and *SI Appendix*, Fig. S1*D*). The plant immune defense is composed of many layers, and we can infer from these results that the failure of nonpathogenic and commensal microbes to continuously increase their population densities is not exclusively determined by full-strength PTI.

### Equilibrium between Bacterial Multiplication and Death Underlying Static Population Densities.

**Fig. 2.**
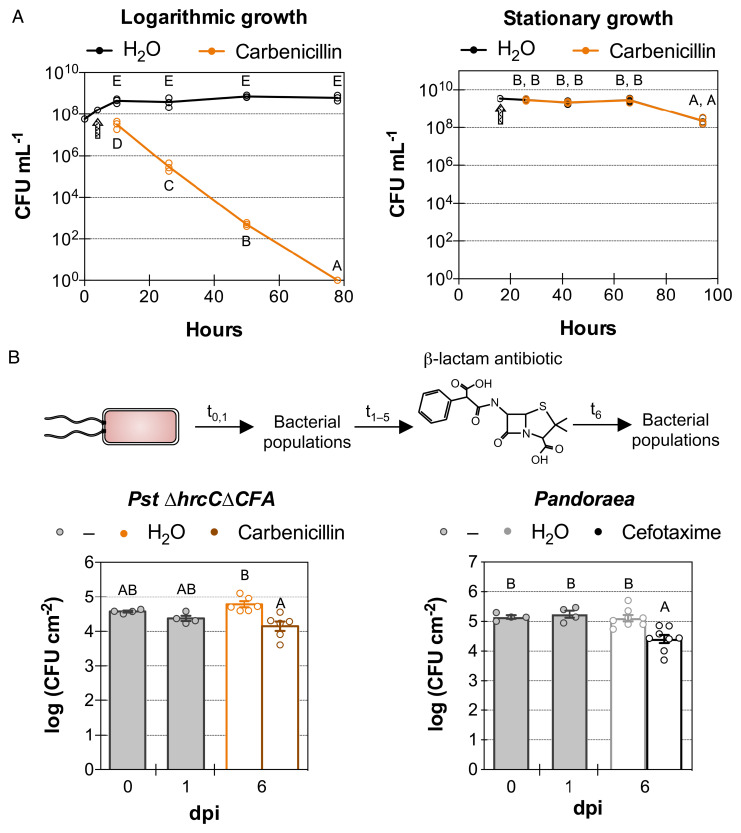
Bacterial population stasis is caused by an equilibrium between bacterial cell death and multiplication. (*A*) In vitro growth of *P. syringae* pv. *tomato* (*Pst*) *ΔhrcCΔCFA* cultures in logarithmic or stationary phase after the addition of 400 µg mL^−1^ of carbenicillin or H_2_O control. The arrow indicates the time at which H_2_O or the antibiotic was added to the culture. The *y* axis is in logarithmic scale. (*B*) In planta bacterial population density of *Pst ΔhrcCΔCFA* or *Pandoraea* sp. Col-0-28 in Col-0 plants after the addition of 400 µg mL^−1^ of carbenicillin or cefotaxime, antibiotics that kill dividing bacteria. Circles show individual biological repetitions; error bars indicate the SEM. Different letters indicate different means (Tukey’s honestly significant difference test; *P* < 0.05). In t_x_, x = number of days postinoculation.

Population density stasis might be reflective of bacterial cells that completely cease to divide or of an equilibrium between the rates of death and multiplication of such cells. To differentiate between these two possibilities, we used β-lactam antibiotics, which only target bacterial cells that are actively dividing (14). We first confirmed the in vitro inhibitory effect of carbenicillin on *Pst ΔhrcCΔCFA* (Fig. 2*A*). As expected, cells in the logarithmic phase were killed by carbenicillin, whereas adding a β-lactam antibiotic when bacteria had reached stationary phase had no effect on their viability (Fig. 2*A*).

To determine whether we can use carbenicillin to kill dividing bacteria inside plants, we inoculated *Arabidopsis* leaves with *Pst* DC3000, followed by consecutive daily infiltrations of carbenicillin. A greater than 100,000-fold increase in population density was observed between 1 and 3 d after inoculation with the virulent pathogen in the absence of carbenicillin, which corresponds to approximately 16 cell divisions per inoculated bacterial cell (*SI Appendix*, Fig. S2*A*). By contrast, in leaves infiltrated with carbenicillin, bacterial population densities were even lower at day 3 than they were before carbenicillin treatment (at day 1). Presumably, more than 99.99% of cells attempted to divide and were killed by the β-lactam antibiotic. We can infer from this result that carbenicillin is very effective in killing dividing *Pst* DC3000 cells within plants.

We next used carbenicillin to evaluate whether in planta population stasis of nonpathogenic *Pst ΔhrcCΔCFA* was a consequence of an equilibrium between multiplication and death or a true cellular stasis that does not allow bacteria to actively divide. We found that population densities of *Pst ΔhrcCΔCFA* treated with the antibiotic were reduced after 5 consecutive daily treatments (at 6 d postinfiltration) when compared to those in the H_2_O-treated control plants (Fig. 2*B*). Approximately 75% of all bacterial cells attempted to divide over 5 d and were killed by the antibiotic. When using experimental conditions that favored more in planta bacterial multiplication (by increasing the relative humidity to over 99%), the effect of carbenicillin was even more dramatic and more than 98% of bacterial cells were killed (*SI Appendix*, Fig. S2*B*).

A similar antibiotic treatment experiment using microbiota strains *Pandoraea* sp. Co-0-28 and *Rhodococcus* sp. 964 yielded similar results. Before in planta experiments, we confirmed that there was little to no effect of the β-lactam antibiotics when cells were in stationary phase (*SI Appendix*, Fig. S2*C*). In planta, 5 d after β-lactam antibiotic treatment, we observed a reduction in bacterial population densities to ∼80% for *Pandoraea* (this experiment used a different β-lactam antibiotic, cefotaxime, as *Pandoraea* is resistant to carbenicillin) (Fig. 2*B*), while for *Rhodococcus* this reduction reached almost 90% (*SI Appendix*, Fig. S2*D*). Overall, these results suggest that nonpathogenic and commensal bacterial endophytes are actively multiplying and dying inside plants, apparently at similar rates. This equilibrium causes microbiota population densities to effectively remain static over time.

### Spatial Visualization of Population Stasis In Planta.

**Fig. 3.**
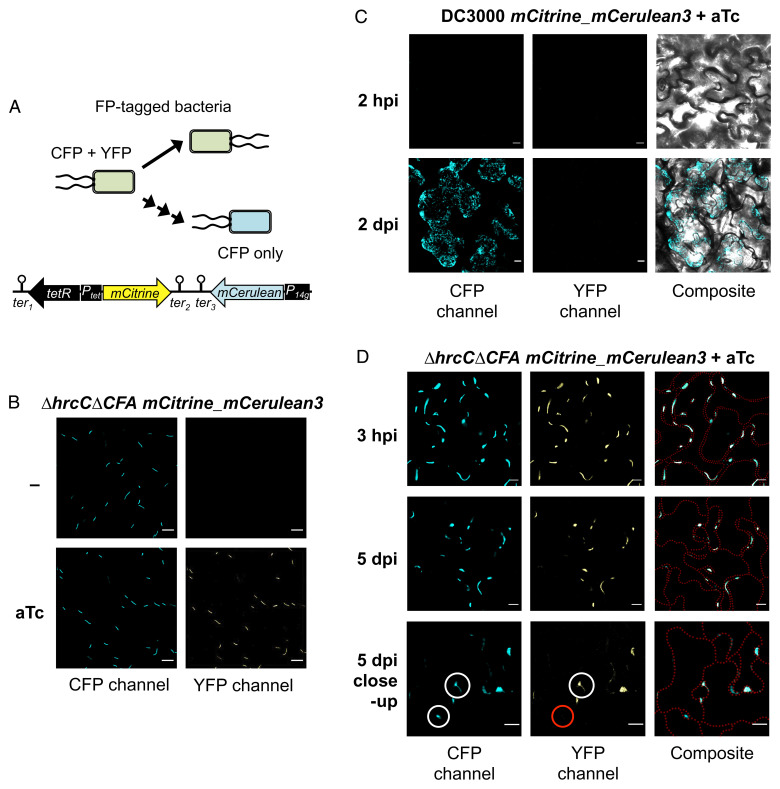
Visualization of bacterial endophyte population stasis in planta. (*A*) A diagram of the DNA construct integrated into the *P. syringae* pv. *tomato* (*Pst*) genome. The tetracycline promoter (*P_tet_*) controls the bidirectional expression of the *tetR* transcriptional regulator and of mCitrine. The strong constitutive *P_14g_* promoter controls the expression of mCerulean3. There are three different transcriptional terminators (*ter*). Above the construct is a schematic representation of the dilution of fluorescent protein (FP) signal when a bacterium divides. If the inducer is absent—as it occurs in planta—and bacteria multiply, only mCerulean3 signal would be observed. (*B*) Confocal images of *Pst ΔhrcCΔCFA* carrying the *P_tet_-mCitrine_P_14g_-mCerulean3* division reporter after in vitro growth with or without the addition of 67 ng µL^−1^ of anhydrotetracycline (aTc) to induce mCitrine expression. (*C*) Confocal images of virulent *Pst* DC3000 carrying the fluorescent division reporter (inoculum: 5 × 10^7^ CFU mL^−1^; grown with 80 ng mL^−1^ aTc) 2 h and 2 d after inoculation of Col-0 plants. Note that fluorescent signal from mCerulean3 is only observed when high bacterial population densities were achieved at 2 dpi. (*D*) Confocal images of nonpathogenic *Pst ΔhrcCΔCFA* carrying the fluorescent division reporter (inoculum: 10^9^ CFU mL^−1^; grown with 80 ng mL^−1^ aTc) 3 h and 5 d after inoculation of Col-0 plants. *Bottom* panels show a close-up of two bacterial aggregates, one with and one without mCitrine signal, indicating a static subpopulation (white circle overlaid on the YFP-channel image) and one that arose from in planta bacterial multiplication (red circle), respectively. Cyan, mCerulean3 signal; yellow, mCitrine signal. Composite image of both FPs is also shown; in *C*, the transmitted light image is overlaid, while in *D*, the cell outlines are overlaid as red dotted lines. (Scale bars, 10 µm.).

Next, we attempted to visualize and differentiate multiplying from static bacteria inside plant leaves. For this purpose, we integrated into the *Pst* genome a reporter that expressed two fluorescent proteins: mCerulean3 (15), expressed constitutively from synthetic promoter 14g (16); and mCitrine (17), expressed under the control of a tetracycline-inducible promoter (18) (Fig. 3*A*). When grown in the presence of a tetracycline analog (anhydrotetracycline), all bacteria carrying the division reporter are expected to be doubly fluorescent for mCerulean3 and mCitrine. If bacteria multiply in the absence of anhydrotetracycline (such as would occur inside plants), the mCitrine signal would become diluted after each cell division. If multiple cell divisions occur, the mCitrine signal would become diluted past the point of detection and the cells would only be detected by the constitutively expressed mCerulean3 signal (Fig. 3*A*). Therefore, static cells that do not multiply would be fluorescent for both fluorescent proteins, while newly divided cells would not express mCitrine and after several divisions would be fluorescent only for mCerulean3.

We first tested the inducibility of our cell division reporter system in vitro. Indeed, *Pst ΔhrcCΔCFA* cells carrying the reporter were detected by only mCerulean3 signal in the absence of anhydrotetracycline and by both mCerulean3 and mCitrine signals when anhydrotetracycline was used (Fig. 3*B*). We then tested our division reporter in planta. Virulent *Pst* DC3000 carrying the reporter was first grown in vitro in the presence of the anhydrotetracycline inducer and then inoculated into *Arabidopsis* Col-0 plants. At this low inoculum density, fluorescent signal from individual bacteria was well below the detection level of our confocal microscopy set-up (day 0) (Fig. 3*C*). However, after 2 d of bacterial growth to form microcolonies in planta, mCerulean3 signal became detectable as bacteria multiplied to population densities well past the detection minimum. mCitrine signal never became detectable in mCerulean3-positive cells, suggesting that most detectable mCerulean3-fluorescent cells arose from newly divided bacteria (Fig. 3*C* and *SI Appendix*, Fig. S3*A*). This result is consistent with the effect of carbenicillin on *Pst* DC3000 multiplication in planta (*SI Appendix*, Fig. S2*A*). After 2 d of infection with *Pst* DC3000, bacteria reached beyond the apoplastic space, colonizing the entirety of multiple plant cells (Fig. 3*C*). Most likely, these plant cells were dead due to bacterial infection, as at later time points *Pst* DC3000 causes wilting and necrosis in *Arabidopsis* (19).

To visualize a nonpathogenic endophyte in planta, *Pst ΔhrcCΔCFA* carrying the cell division reporter was grown in vitro in the presence of the anhydrotetracycline inducer and then inoculated into *Arabidopsis* leaves. At 3 h postinoculation, *Pst ΔhrcCΔCFA* had colonized multiple noncontinuous regions of the leaf apoplast. Both mCerulean3 and mCitrine signals were observed, and their signals overlapped (Fig. 3*D*). Observed bacterium-colonized apoplast areas are most likely those in which a liquid environment is present. Only in pathogenic organisms, the virulence mechanisms would allow access to other areas by using effectors that promote the water soaking of the entire leaf apoplast (4) (Fig. 3*C*). Interestingly, on day 5, signal from both fluorescent proteins in *Pst ΔhrcCΔCFA* was still observed (Fig. 3*D*). However, at this point, while some bacterial aggregates had signal from both fluorescent proteins, others had signal only from mCerulean3 (Fig. 3*D* and *SI Appendix*, Fig. S3*B*). This indicates that some cells of the *Pst ΔhrcCΔCFA* population have not divided and remain static (for at least 5 d postinoculation [dpi]), while other cells have divided and given rise to new bacterial microcolonies (those devoid of mCitrine signal). These results agree with the results obtained with the β-lactam antibiotic treatment (Fig. 2*B* and *SI Appendix*, Fig. S2*B*), in which some of the *Pst ΔhrcCΔCFA* cells in the population divided while others remained in stasis.

### In Planta Transcriptomics Analysis of Phyllosphere-Inhabiting Bacteria.

To understand which bacterial biological processes are affected during long-term colonization of leaves by nonpathogenic and commensal bacteria, we performed RNA sequencing (RNA-Seq) of *Arabidopsis* Col-0 leaves inoculated with two commensal microbiota strains, *A. xylosoxidans* Col-0-50 and *Pandoraea* sp. Col-0-28, and with *Pst ΔhrcCΔCFA* (20). In planta bacterial transcriptomes were evaluated at 6, 24, and 168 h (7 d) after inoculation and compared to their respective inocula. We selected 168 h as the last time point for our experiment with the expectation that at this time point, bacteria would have adapted to life in the apoplast and would more closely resemble the steady state of natural microbiota colonization. This is in contrast to previous in planta bacterial transcriptomic studies, which tend to focus on the earlier events during the transition of the endophyte from nutrient-rich artificial media to the plant environment, a condition that may not be prevalent in nature (10, 21). For *Pst ΔhrcCΔCFA*, we also included in vitro populations of cells that were in the logarithmic or stationary phase of growth. The bacterial population densities in the samples used for sequencing are shown in *SI Appendix*, Fig. S4 *A–C*. To be thorough in calculating differential gene expression (DGE) in the RNA-Seq analysis, we used three different methods to determine DGE (*SI* *Appendix*, *Supplementary Materials and Methods*; for a comparison, see *SI Appendix*, Fig. S4*D*). A principal component analysis (PCA) indicated that all the samples of every treatment clustered together (*SI Appendix*, Fig. S4 *E* and *F*).

In planta gene expression patterns of select biological pathways that were enriched in differentially expressed genes in at least one of the different treatment comparisons are presented in *SI Appendix*, Fig. S4*G* (for *Pst ΔhrcCΔCFA*; also see Fig. S5), S4*H* (for *A. xylosoxidans*), and S4*I* (for *Pandoraea* sp.). Below, we highlight bacterial biological processes whose gene expression was affected inside plants during endophyte colonization.

#### Primary metabolism.

**Fig. 4.**
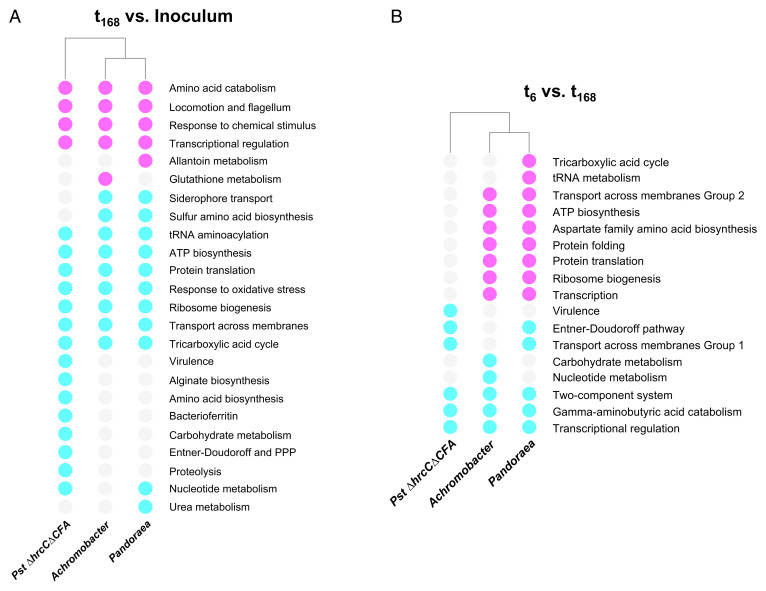
Long-term transcriptome analysis of three phyllosphere-inhabiting endophytic bacteria. Up- or down-regulation of expression of enriched biological processes based on differentially expressed groups of orthologous genes shared or not between *P. syringae* pv. *tomato* (*Pst*) *ΔhrcCΔCFA* and microbiota strains *Achromobacter xylosoxidans* Col-0-50 and *Pandoraea* sp. Col-0-28, when comparing (*A*) in planta samples at 168 h postinoculation (t_168_) to the in vitro grown inoculum or (*B*) in planta samples at 6 h postinoculation (t_6_) to in planta samples at 168 h postinoculation. Lack of enrichment is indicated by gray circles. Enriched down-regulated processes are indicated by magenta circles, while those up-regulated are indicated by cyan circles. PPP, pentose phosphate pathway.

For *Pst ΔhrcCΔCFA*, expression of genes associated with primary metabolic pathways for energy and intermediary metabolite generation involved in adenosine triphosphate (ATP) biosynthesis, sulfur metabolism (only during initial plant colonization) (*SI Appendix*, Table S1), and hexose catabolism, including the Entner–Doudoroff pathway, tricarboxylic acid (TCA) cycle, and the pentose phosphate pathway (*SI Appendix*, Fig. S5), were up-regulated inside plants. Expression of TCA, ATP biosynthesis, and sulfur metabolism genes was also up-regulated in planta when compared to the inoculum for microbiota strains *A. xylosoxidans* Col-0-50 and *Pandoraea* sp. Col-0-28 (Fig. 4*A* and *SI Appendix*, Tables S2 and S3); interestingly, however, only in the microbiota strains was expression of ATP biosynthesis genes up-regulated the longer the bacteria stayed inside the leaves (Fig. 4*B*).

#### Protein translation and gene transcription.

Similarly, expression of protein translation genes, especially those involved in ribosome biogenesis, was up-regulated inside plants when compared to the inoculum for all three endophytes (Fig. 4*A* and *SI Appendix*, Fig. S4 *G–I*). For *Pst ΔhrcCΔCFA*, tRNA-coding genes had an expression pattern opposite to genes involved in ribosome biogenesis; expression was lower inside plants than in the inoculum irrespective of the time point analyzed (*SI Appendix*, Fig. S5*E*). Like the expression of ATP biosynthesis genes mentioned above, expression of protein translation–related genes as well as genes directly involved in transcription, such as the different RNA polymerase subunits, was higher in both microbiota endophytes after 7 d postinoculation (t_168_) compared to earlier inoculation times, while no difference was observed for *Pst ΔhrcCΔCFA* (Fig. 4*B*). On the contrary, some transcriptional regulation processes were reduced in all three strains at 168 h postinoculation (hpi) when compared to earlier time points (Fig. 4*B* and *SI Appendix*, Tables S4–S6).

#### Stress adaptation.

As previously reported, expression of alginate biosynthesis genes and virulence-promoting mechanisms—including genes for coronatine biosynthesis and type III secretion system structural components and effectors—was up-regulated inside plants for *Pst ΔhrcCΔCFA* compared to inocula grown in media (*SI Appendix*, Figs. S4*G* and S5) (10). Interestingly, however, expression of these virulence-promoting mechanisms in *Pseudomonas* decreased inside plants over time (Fig. 4*B*). The response to oxidative stress was up-regulated in planta for all three strains (Fig. 4*A*), and for *A. xylosoxidans*, so was the osmotic response (*SI Appendix*, Table S2), perhaps as an adaptation to the harsh environment encountered by microbes in the plant apoplast. For all three endophytes, expression of two-component systems was reduced inside plants over time (Fig. 4*B*), suggesting that sensing and responding to the plant environment are more important during the initial phase of interaction, as opposed to long-term adaptation of the microbe with its host.

#### Chemotaxis and flagellum biosynthesis.

For all three endophytes, expression of chemotaxis and flagellum biosynthesis genes was lower in plants when compared to the inoculum (Fig. 4*A*). Flagellum biosynthesis genes were down-regulated even further as time inside plants went by for *Pst ΔhrcCΔCFA* and *A. xylosoxidans* (*SI Appendix*, Fig. S5 and Tables S4 and S5). Flagellum biosynthesis repression may be reflective of the strong pressure that flagellin detection by plants has over microbiota adaptation (22).

#### Secondary metabolites.

As secondary metabolites can play an important role in regulating microbe–microbe and plant–microbe interactions, we used antiSMASH (23) to identify potential secondary metabolite biosynthesis gene clusters in the three phyllosphere-inhabiting bacteria (*SI Appendix*, Tables S7 and S8). The analysis confirmed the presence of known secondary metabolite clusters in *Pst ΔhrcCΔCFA*, such as those for the biosynthesis of the phytotoxin coronatine and the siderophores pyoverdin and yersiniabactin. Other than those three clusters, a region involved in the biosynthesis of the dipeptide *N*-acetylglutaminylglutamine amide (NAGGN) showed a clear and stable up-regulation inside plants (*SI Appendix*, Table S7). NAGGN might function in osmoregulation, as has already been shown in other bacteria (24), and protect *Pst ΔhrcCΔCFA* from osmotic stress. No differentially expressed secondary metabolite biosynthetic clusters were observed in *Pandoraea* sp., while in *A. xylosoxidans*, expression of a cluster involved in the biosynthesis of the osmoprotectant ectoine was up-regulated in plants, especially early during plant colonization (*SI Appendix*, Table S8). This result independently confirmed the findings of the biological pathway enrichment analysis done previously (*SI Appendix*, Tables S2 and S5). A secondary metabolite cluster in *A. xylosoxidans* for the production of resorcinol, which could potentially have antimicrobial activity (25), was induced early in the interaction of *A. xylosoxidans* with plants, perhaps because this metabolite confers growth competition advantages. Finally, expression of genes predicted to be involved in the biosynthesis of a desferrioxamine-like siderophore was down-regulated inside plants in *A. xylosoxidans*, which suggests that this endophyte may not experience iron limitation, consistent with what has been observed during pathogenic infections of *Pst* DC3000 (26).

#### “Plant-associated” genes.

Recently, bacterial plant-associated (PA) genes were identified in a large-scale comparative metagenomics study using almost 4,000 bacterial genomes (27). We expected that PA genes would be enriched among genes up-regulated inside plants when comparing their expression to that of the inoculum, potentially highlighting the importance of PA genes for adaptation to plant survival. Surprisingly, however, no in planta up-regulated PA gene enrichment was observed when compared to the inoculum (t_6_, t_24_, or t_168_ vs. the inoculum) (*SI Appendix*, Table S9). For *Pst ΔhrcCΔCFA* and *Pandoraea*, within in planta comparisons, there was enrichment of up-regulated PA genes at earlier time points (t_6_ vs. t_24_ or t_168_) (*SI Appendix*, Table S9). For *Pst ΔhrcCΔCFA*, there was enrichment of up-regulated genes when the in planta populations were compared to those grown in liquid culture (logarithmic- and stationary-phase populations), as opposed to those grown in agar plates (the inoculum) (*SI Appendix*, Table S9).

### Stationary-Phase–Like Transcriptomic Features of Nonpathogenic Endophytes.

**Fig. 5.**
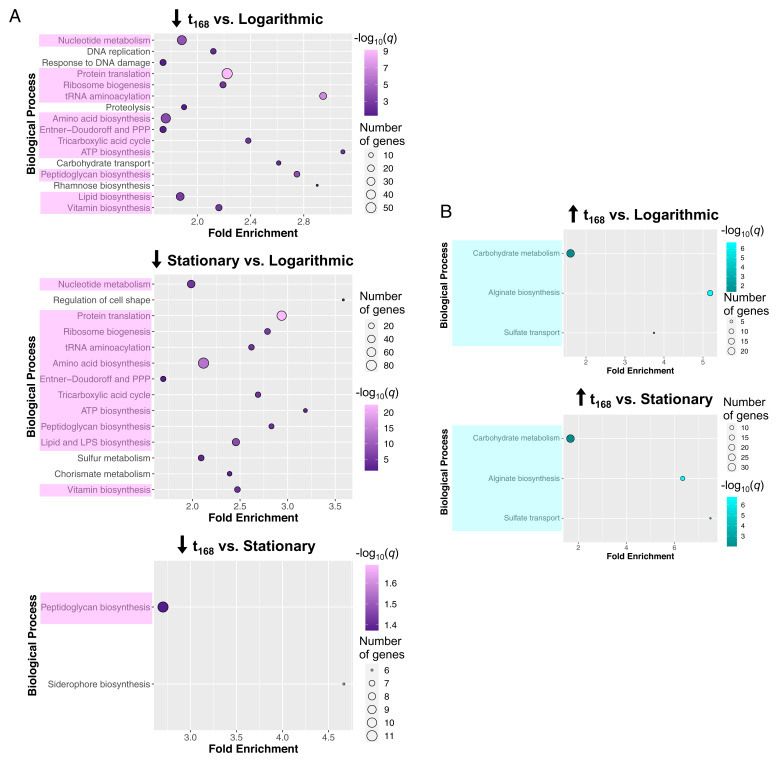
Static bacterial populations show similar transcriptional responses to populations in stationary phase. (*A*) Bubble graphs of enriched biological processes in differentially expressed genes (DEGs) down-regulated at 168 h postinoculation (t_168_) or in in vitro stationary-phase populations, when compared to in vitro logarithmic-phase populations, or when comparing t_168_ to stationary phase. Processes found in at least two comparisons are highlighted in pink. (*B*) Bubble graphs of biological processes from up-regulated DEGs that are exclusively enriched in planta using the same comparisons as in *A*. The fold enrichment, number of significant DEGs, and the log_10_ of the adjusted *P* value (*q*) are shown for each biological process. Only processes with at least four genes are shown.

Further comparisons of the in planta transcriptome with in vitro logarithmic- and stationary-phase transcriptomes of *Pst ΔhrcCΔCFA* revealed a striking feature. As expected, in a PCA of the expression of all genes, we observed that overall gene expression in vitro was more similar between in vitro populations (the inoculum and logarithmic and stationary populations) than with the overall gene expression observed inside plants (see PCAs in *SI Appendix*, Fig. S4 *E* and *F*). However, when we compared the enriched biological processes in down-regulated DEGs at 168 hpi in planta vs. the in vitro inoculum to those observed when comparing in vitro stationary vs. in vitro logarithmic phase (i.e., enrichment analysis) (*SI Appendix*, Table S1), we found globally similar enriched down-regulated biological processes, including processes involved in protein translation and the generation of metabolite precursors and energy (Fig. 5*A*). This similarity extends to two enriched biological processes in up-regulated DEGs: flagellum biosynthesis and two-component systems (*SI Appendix*, Fig. S6). The similarities in enriched biological processes between the transcriptome of in vitro stationary phase and 168 hpi in planta populations reinforces the idea that bacteria inside plants more closely resemble in vitro bacteria that have reached stationary phase and correlates with their inability to increase in population density inside plants.

Interestingly, we observed that the in planta transcriptome at 168 hpi has unique features that are not shared with the in vitro transcriptomes (irrespective of populations being in logarithmic or stationary phase). For example, alginate biosynthesis and sulfate transport were up-regulated after 7 d of endophytic lifestyle when compared to the in vitro logarithmic or stationary phase transcriptomes (Fig. 5*B*), suggesting that the overall stationary phase-like *Pst ΔhrcCΔCFA* in planta lifestyle has unique features that are not identical to the stationary phase in culture.

### ETI-Inducing Bacteria Experience Population Stasis.

**Fig. 6.**
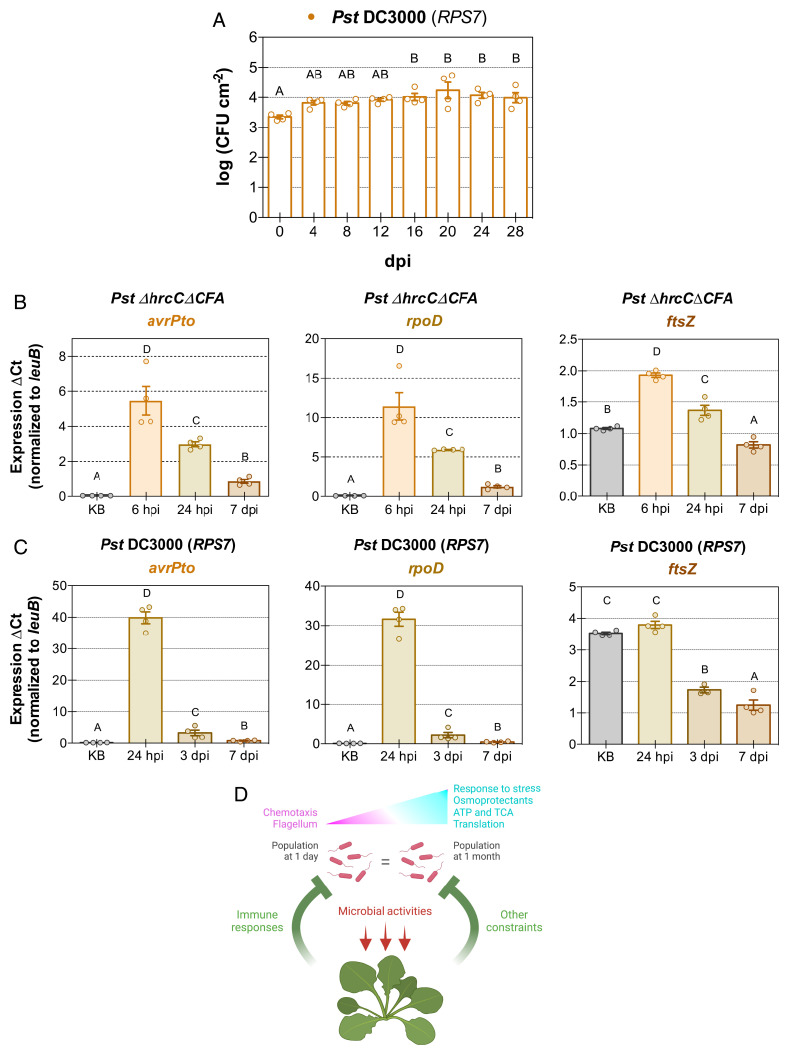
Bacteria activating effector-triggered immunity experience stasis. (*A*) Bacterial population density of *P. syringae* pv. *tomato* (*Pst*) DC3000 over the course of 28 d after infection of *Arabidopsis* accession Bu-22, which carries *RPS7*, a gene that recognizes *Pst* DC3000 effector AvrPto, and initiates effector-triggered immunity. (*B*) In planta gene expression of a type III effector (*avrPto*), the housekeeping RNA polymerase sigma factor (*rpoD*), and a gene involved in bacterial cell division (*ftsZ*) after inoculation with *Pst ΔhrcCΔCFA* into Col-0 plants. (*C*) In planta gene expression of the same genes as in *B* after inoculation with *Pst* DC3000 into Bu-22 plants. (*D*) Model illustrating the interaction between phyllosphere endophytes and plants. Circles show individual biological repetitions; error bars indicate the SEM. Different letters indicate different means (Tukey’s HSD test; *P* < 0.05, which was performed between the ΔCt values for qRT-PCR). For qRT-PCR, gene expression was evaluated using *leuB* as the reference gene and the ΔCt method. KB, bacterial inoculum; TCA, tricarboxylic acid cycle.

Phyllosphere-inhabiting microbiota also include pathogenic strains that trigger ETI in genetically resistant plants, and are therefore unable to cause disease. We wanted to compare the population dynamics and gene expression of nonpathogenic endophytes to that of ETI-inducing phyllosphere bacteria. Bu-22 is an *Arabidopsis* accession in which *Pst* DC3000 triggers ETI due to recognition of effector AvrPto by RPS7 (RESISTANCE TO PSEUDOMONAS SYRINGAE 7) (19). We found that the *Pst* DC3000 population increased slightly very early in the interaction with Bu-22 (before 4 dpi), after which the bacterial population densities remained static for 24 d (Fig. 6*A*). We also tested additional ETI-inducing strains containing effectors AvrRpt2, AvrRps4, and AvrPphB—recognized by RSP2, RPS4, and RPS5, respectively—and observed similar long-term population stasis (other than the small initial population density increase) (*SI Appendix*, Fig. S7*A*). Taken together, these results revealed a common theme for phyllosphere-inhabiting endophytic commensal, ETI-inducing, and nonpathogenic bacteria: the plant immune system maintains the microbial population numbers without fully eliminating bacteria from the plant apoplast.

Next, we conducted targeted quantitative PCR analysis to compare gene expression for nonpathogenic *Pst ΔhrcCΔCFA* with that for *Pst* DC3000 after AvrPto recognition. We selected genes involved in virulence (the virulence sigma factor *hrpL* and the type III effector *avrPto*) (28, 29), transcription (the housekeeping RNA polymerase sigma factor *rpoD*) (30), and bacterial cell division (*ftsZ*) (31). In *Pst ΔhrcCΔCFA*, *avrPto*, *hrpL*, and *rpoD*, gene expression was extremely low in the inoculum, increased exponentially by 6 h after inoculation into plants, and then decreased over time, with the lowest level by 168 h (Fig. 6*B* and *SI Appendix*, Fig. S7*B*). *ftsZ* showed the same trend of early increased expression and subsequent decrease in planta; however, the relative expression in the inoculum was more variable (Fig. 6*B* and *SI Appendix*, Fig. S7*C*). The same gene expression pattern as that observed with *Pst ΔhrcCΔCFA* was observed under ETI-inducing conditions with *Pst* DC3000 for *avrPto*, *hrpL*, *rpoD*, and *ftsZ* (Fig. 6*C* and *SI Appendix*, Fig. S7 *C* and *D*), with the highest expression for each of these genes observed at 24 h after inoculation into Bu-22 plants, and then decreasing afterward. We evaluated the expression of a few additional genes under ETI conditions, as we were unable to perform long-term RNA-Seq of ETI-inducing strains using a high inoculum (*SI Appendix*, *Supplementary Materials and Methods*). We selected an additional gene involved in virulence (the phytotoxin biosynthesis gene coronafacate ligase *cfl*) (8), a gene involved in the last step of the Entner–Doudoroff pathway (the pyruvate kinase *pyk*) (32), and a gene involved in protein translation (tRNA^fMet^-formyl transferase *fmt*, which formylates the initiator tRNA^fMet^ in eubacteria) (33). Expression of these three genes showed a temporal pattern similar to that observed previously; gene expression was very low in the inoculum, increased by 24 h after inoculation, and decreased at later time points (*SI Appendix*, Fig. S7*D*).

## Discussion

In this study, we characterized the long-term population dynamics of several representative phyllosphere endophytic bacteria. Our long-term evaluation of phyllosphere endophytic bacteria, in contrast to previous reports that focused on the early events after infections, are more likely reflective of the long-term association that various bacterial microbiota members experience inside plants in nature. We observed that population densities of commensal microbiota strains as well as nonpathogenic or ETI-inducing strains of the model phyllosphere pathogen *P. syringae* pv. *tomato* DC3000 remained static for weeks after introduction into the leaf apoplast and were not eliminated in planta. Static population densities of phyllosphere bacteria suggest that plants may have evolved to “ignore” endophytic bacteria that are below a certain population density threshold (below 10^6^ CFU cm^−2^) (Fig. 1*C*) or, perhaps, most phyllosphere microbiota bacteria might have evolved to maintain low population densities in order to not alert the immune system of their presence. Alternatively, plants could be actively controlling bacterial populations irrespective of the population density. The prevalent population stasis is caused by equilibrium in the rates of multiplication and death of these bacteria, and apparently not due to a lack of sufficient resources in the apoplast or an appropriate niche for microbial growth. In planta bacterial transcriptomes suggested that nonpathogenic bacterial physiology resembles the physiology that bacteria experience during the stationary phase, a condition in which housekeeping gene expression is reduced, while allowing selective expression of certain biological processes including those important for survival against stress (Fig. 6*D*) (34).

Our results raise several conceptual issues that are worth discussing. First, we found it surprising that the long-term population outcomes of examined bacterial strains were similar. We had initially hypothesized that plant immune responses may have differential effects on potential pathogens vs. commensal microbiota. For example, plants may eventually eliminate ETI-inducing pathogen populations while maintaining a steady population density of commensal microbiota, as the former poses a threat to plants in the long term and the latter may be needed for performing beneficial functions under certain conditions. Instead, we found that ETI-inducing pathogenic strains and nonpathogenic bacteria all could maintain a static population for several weeks (Figs. 1 and 6*A* and *SI Appendix*, Figs. S1*A* and S7*A*). Interestingly, population density stasis was observed during ETI interactions after activation by both NLRs of the coiled-coil and TIR classes of resistance proteins (RPS2 and RPS5 are of the coiled-coil class, while RPS4 is a TIR NLR) (*SI Appendix*, Fig. S7*A*) (6), suggesting that this phenomenon is widespread. The initial growth observed in the first days after inoculation for ETI-inducing strains implies that ETI-mediated population restriction mechanisms have yet to become active. In the host–pathogen battle experienced by ETI-inducing bacteria, the initial gains by bacterial virulence mechanisms seem to be outdone by the plant immune system at later time points. It is also notable that niche destruction caused by the hypersensitive response of the host cells is not enough to eliminate bacteria.

Furthermore, gene expression in *Pst* DC3000 after plants undergo ETI showed a pattern reminiscent of the one observed for nonpathogenic *Pst ΔhrcCΔCFA* (Fig. 6 *B* and *C*), highlighting further similarities between these two static population interactions. Under ETI conditions, there was a dramatic reduction of expression over time of genes involved in virulence (Fig. 6*C* and *SI Appendix*, Fig. S7*D*). In particular, the down-regulation of *avrPto*, which in *RPS7*-carrying plants betrays *Pst* DC3000 bacteria by alerting plants of their presence, might be an adaptation to quench the strong immune response experienced by bacteria during ETI, and “transform” *Pst* DC3000 into a nonpathogenic persisting microbe. Alternatively, this down-regulation might be due to a lack of type III secretion system–inducing compounds present in the plant apoplast during ETI, as has been previously suggested during PTI activation (35).

Our in planta transcriptome analysis also revealed an interesting difference between the transcriptomes of commensal *A. xylosoxidans* Col-0-50 and *Pandoraea* sp. Col-0-28 and that of *Pst ΔhrcCΔCFA* that may suggest a unique adaptation of commensal microbiota strains to the apoplast environment. Specifically, once bacteria were in the apoplast, the expression of genes involved in protein translation and ATP biosynthesis exhibited a sustained increase over time for the two-microbiota endophytes. In contrast, no such sustained increase was observed for *Pst ΔhrcCΔCFA*, suggesting that microbiota strains have developed a better adaptation than *Pst ΔhrcCΔCFA* for long-term survival inside plants. All three phyllosphere bacteria have adapted to colonize the apoplast and there was an induced expression of mechanisms involved in the response to stress, including genes putatively involved in the biosynthesis of osmoprotectants: *N*-acetylglutaminylglutamine for *Pst ΔhrcCΔCFA* and ectoine for *A. xylosoxidans*.

Although our detailed transcriptome analysis of *Pst ΔhrcCΔCFA* revealed features of a stationary phase–like state, this does not mean that bacteria are not metabolically active. For example, during stationary phase, protein expression is constant (36). Also, it is during such phase that secondary metabolites, such as osmoprotectants and competition-facilitator antibiotics, are synthesized (34). In culture, bacteria enter the stationary phase in part because of depletion of nutrients (37). Our observation of population stasis at different bacterial densities (three order of magnitudes), however, suggests that it is not a lack of resources alone that prevents bacterial endophytes from multiplying (Fig. 1*C*).

We did not observe enrichment of plant-associated genes [as determined by Levy et al. (27)] in the genes up-regulated inside plants when compared to the inoculum. This appears to be inconsistent with what has recently been observed for eight microbiota strains (38). However, if we compare in vitro planktonic populations grown in liquid media to those of populations grown inside plants for *Pst ΔhrcCΔCFA*, we do observe enrichment of up-regulated PA genes (*SI Appendix*, Table S9). The inoculum used for our transcriptome analysis was grown in solid agar plates while the inoculum for the Nobori et al. (38) study was grown in liquid media. Perhaps, solid agar mimics more closely the cues for the up-regulation of PA genes inside the leaf apoplast. If true, our results could provide indirect support for a long-standing observation that inert wooden splinters partially mimic plant roots for the assembly of root microbiota (39).

We provided multiple lines of evidence that population stasis in nonpathogenic microbiota was caused by an equilibrium in bacterial multiplication and death, as demonstrated by the killing of any bacteria that attempted to divide under β-lactam antibiotic treatment (Fig. 2*B* and *SI Appendix*, Fig. S2 *B* and *D*). This was further confirmed using a fluorescent division reporter (Fig. 3*D*), which allowed us to observe actively multiplying bacterial microcolonies in planta. It was also apparent that there is a fine-scale phenotypic heterogeneity of the clonal population of microbiota in plants: Some bacteria are physiologically able to multiply and are sensitive to β-lactam antibiotic–mediated killing, while others are in a β-lactam antibiotic–resistant quiescent state. Further understanding of the observed heterogeneity in endophytic bacterial populations will require adaptation and optimization of current techniques for single-cell bacterial RNA-Seq (40, 41) for future in planta transcriptomics. A better understanding of the nature of the long-term population dynamics and transcriptomic features associated with an endophytic commensal or pathogenic lifestyle sets a foundation for engineering of commensal microbiota or development of more effective means of pathogen population inhibition in agricultural settings.

## Materials and Methods

All experiments reported in this study were done at least thrice, except for the RNA-Seq experiments, which had a single experimental repeat with four biological replicates (n) per treatment. Detailed methods for bacterial population density quantification, gene cloning, gene integration of the fluorescent division reporter into the genome of *P. syringae*, in planta confocal microscopy of bacteria carrying the fluorescent division reporter, qRT-PCR, RNA-Seq, and subsequent bioinformatics analyses can be found in *SI Appendix*, *Supplementary Materials and Methods*.

## Supplementary Material

Supplementary File

## Data Availability

RNA-sequencing reads data have been deposited in Sequence Read Archive (SRA) (BioProject PRJNA738276).
