# Supplementary material for: Shared in planta population and transcriptomic features of nonpathogenic members of endophytic phyllosphere microbiota
Source: Proc Natl Acad Sci U S A. 2022 Mar 28;119(14):e2114460119. doi: 10.1073/pnas.2114460119 (PMC9168490; doi:10.1073/pnas.2114460119)
Supplement: Supplementary File [file pnas.2114460119.sapp.pdf]

PNAS
www.pnas.org

SUPPLEMENTARY INFORMATION FOR:

Shared *in planta* population and transcriptomic features of non-pathogenic members of endophytic phyllosphere microbiota

André C. Velásquez[1], José C. Huguet-Tapia[2], and Sheng Yang He[1,3]

[1]Howard Hughes Medical Institute, Duke University, Durham, NC 27708, USA; [2]Department of Plant Pathology, University of Florida, Gainesville, FL 32611, USA; and [3]Department of Biology, Duke University, Durham, NC 27708, USA.

André C. Velásquez
E-mail address: andre.velasquez@duke.edu.
Sheng Yang He
E-mail address: shengyang.he@duke.edu.

This PDF file includes:

Supplementary text
Figures S1 to S8
Tables S1 to S15
Legend for Datasets S1 to S3
SI References

Other supplementary materials for this manuscript include the following:

Datasets S1 to S3

SUPPLEMENTARY TEXT

SUPPLEMENTARY MATERIALS AND METHODS

In vitro bacterial growth and antibiotics used

Table S10 lists the bacterial strains used in this study. *Escherichia coli* strains were grown in LB (Lennox) medium at 37 °C, while all other strains were grown in either a modified LB medium (LM: 10 g L^{-1} tryptone, 6 g L^{-1} yeast extract, 1.5 g L^{-1} KH_2PO_4, 0.6 g NaCl, and 0.4 g $MgSO_4$•$7H_2O$) or King's B medium at 30 °C.

Antibiotics and derivatives were used at the following concentrations: 100 µg mL^{-1} ampicillin, 67–80 ng mL^{-1} anhydrotetracycline, 400 µg mL^{-1} carbenicillin, 400 µg mL^{-1} cefotaxime, 75 µg mL^{-1} cycloheximide (to prevent fungal growth), 1–15 µg mL^{-1} gentamycin, 50 µg mL^{-1} kanamycin, 100 µg mL^{-1} rifampicin, and 50 µg mL^{-1} spectinomycin. Diaminopimelic acid (DAP) was used at a concentration between 200–400 µg mL^{-1}.

To evaluate the response of logarithmic-phase *Pseudomonas syringae* pv. *tomato* (*Pst*) *ΔhrcCΔCFA* and stationary-phase *Pst ΔhrcCΔCFA*, *Pandoraea* sp. Col-0-28, and *Rhodococcus* sp. 964 cultures to the β-lactam antibiotics carbenicillin or cefotaxime, bacteria were grown in liquid LM overnight at 30 °C. For logarithmic-phase samples, overnight bacterial suspensions were diluted with LM to 10^8 CFU mL^{-1}, and grown for 4 hours before antibiotic treatment. Stationary-phase samples were not diluted. H_2O control or 400 µg mL^{-1} carbenicillin or cefotaxime were added to the cultures and grown for over 3 days. Before plating serial dilutions of the samples to determine population densities, cultures were washed twice with 10 mM $MgCl_2$ to remove any trace of the antibiotic from the bacterial suspension.

Plant growth conditions

Arabidopsis plants were grown in a growth chamber with the following conditions: 12-hour day length, 80 µmoles m^{-2} s^{-1} of photon flux, a temperature of 24.5 °C during the day and 23.0 °C during the night, and a relative humidity between 65% and 75%. Table S11 lists the plant material used for this study. For some experiments (Figure S2*B*, and for the *in planta* RNA-Seq samples of *Achromobacter xylosoxidans* and *Pandoraea*), the relative humidity was increased to be higher than 99% after bacterial inoculation. Seeds were stratified for 2 to 5 days before sowing. Plants were watered with one-half strength Hoagland's solution when needed. All plants were grown partially covered under a plastic transparent dome.

Bacterial population density quantification assays

Bacterial inoculum suspensions were prepared from cultures grown in LM or KB agar plates to stationary phase. Bacteria were resuspended in 0.25 mM $MgCl_2$ to an appropriate OD_{600}, after which they were infiltrated using a needleless syringe into the abaxial side of Arabidopsis leaves. To determine the *in planta* population density numbers, leaf-disc punches were collected from plants and ground in 250 µL of 10 mM $MgCl_2$ using a TissueLyser II (QIAGEN; 2 cycles of 30 seconds at 25 Hz) and 3-mm zirconium oxide beads (Glen Mills Inc.). Serial dilutions of the ground tissue were spotted onto LM plates with appropriate antibiotics

and grown overnight. Colony forming units (CFUs) per cm^2 were determined for each sample, while CFUs per mL were determined for each inoculum.

To determine endophytic bacterial populations, leaves were placed in a 0.825% sodium hypochlorite solution for 1 minute, and then washed twice in distilled H_2O, each for a minute. Leaves were blotted dry, after which leaf discs were collected as described above.

As described before (1), the *in planta* transcriptome analysis requires an inoculum greater than 10^9 CFU mL^{-1}; otherwise the sequencing would be overwhelmed by mostly plant RNAs. The observed slight decrease in population densities between 6 and 24 hours (Figure S4*A*, S4*B*, and S4*C*) could be caused by a strong initial PTI response due to the overabundance of MAMPs in the inoculum.

For *in planta* antibiotic treatments, 400 μg mL^{-1} of a β-lactam antibiotic, carbenicillin or cefotaxime, was infiltrated into plants every day until the end of the experiment starting at 1 day post-inoculation with the bacterial endophytes. H_2O was used as a mock control. To calculate the percentage of cells that attempted to divide, we divided the average CFU cm^{-2} treated with the β-lactam antibiotic by the average CFU cm^{-2} mock-treated with H_2O, multiplied this by 100, and subtracted the result from 100.

Cloning

The dual fluorescent division reporter construct pUC18-mini-Tn7T-Gm::*tetR(BD)-P_{tet}-mCitrine_mCerulean3-BCD2-P_{14g}* was created in two steps. In the first step, a DNA fragment containing a strong transcriptional terminator, TpheA-1 (2), repressor *tetR(BD)* [sequence is the fusion of two alleles of *tetR*, carrying 50 codons of *tetR(B)* and the last 158 codons of *tetR(D)* (3)], the tetracycline promoter from *E. coli* Tn10 (which controls expression of both *tetR(BD)* and *mCitrine*; GenBank accession number AF162223), fluorescent protein mCitrine (4), and two strong transcriptional terminators in opposite orientation, ECK125109870 and L3S2P56 (2), was synthesized by GenScript®. This DNA fragment was cloned into pUC18-mini-Tn7T-Gm (5) (cut with NsiI and StuI [New England Biolabs®, Inc.]) using Gibson assembly.

In the second step, two DNA fragments were PCR amplified using primer pairs AVL001 and AVL002, and AVL003 and AVL004 (Table S12): one amplicon included the promoter P14g and translational coupler BCD2 from plasmid pBG42 (6), while the other amplicon included the gene coding for the fluorescent protein mCerulean3 (7). These fragments were introduced into the previously generated intermediate plasmid (after cutting the plasmid with SpeI [New England Biolabs®, Inc.]) by Gibson assembly to generate the division reporter pUC18-mini-Tn7T-Gm::*tetR(BD)-P_{tet}-mCitrine_mCerulean3-BCD2-P_{14g}* (Figure 3*A*).

Gene integration into the *P. syringae* genome

Integration into the *Pst* genome was performed by site-specific transposition by Tn7 into the unique attTn7 site of *Pst* DC3000. For this, triparental mating was set up between the *Pst* recipient strain, the strain containing the transposase helper plasmid pTNS3 (8), and the *E. coli* RHO5 donor strain (9) (carrying plasmid pUC18-mini-Tn7T-Gm with the genes to be integrated). Three to six days after conjugation and selection in plates containing 1.5 μg mL^{-1} of gentamycin, colony PCRs were set up to identify the putative

transconjugants using three primer pairs: AVL005 and AVL006, AVL007 and AVL008, and AVL005 and AVL007 (Table S12).

Confocal microscopy

Images were taken using the Nikon A1Rsi confocal microscope with a 20X objective (numerical aperture of 0.7 and a pinhole of 1.2 airy units). The CFP and YFP channels are detected using gallium arsenide phosphide photomultiplier (PMT) detectors. For the CFP channel, an excitation of 443.6 nm and an emission between 467 to 502 nm were used. For the YFP channel, an excitation of 513.9 nm and an emission of 530 to 600 nm were used. Images were acquired at a gain and offset at which the negative control (Arabidopsis plants infected with *Pst* DC3000 or *Pst* Δ*hrcC*Δ*CFA* without the fluorescent division reporter) had no signal being detected.

To determine the distribution of the overlap in signal intensity between mCitrine and mCerulean in segmented regions of interest, we used ImageJ (version 2.3.0/1.53f).

RNA extraction

For *A. xylosoxidans*, we used only the endophytic bacteria by eliminating the surface-inhabiting bacteria with sodium hypochlorite before RNA extraction. Frozen plant tissue was ground using a TissueLyser II (QIAGEN; 2 cycles of 30 seconds at 27 Hz) and 3-mm zirconium oxide beads (Glen Mills Inc.). RNA was extracted using TRIzol™ (Thermo Fisher Scientific) and the Direct-zol RNA miniprep kit (Zymo Research), using a 30-minute on-column treatment with 12 U of DNase I. A second DNase I treatment to remove all residual genomic DNA was performed using 30 U of recombinant DNase I (Roche) and 10 U of Protector RNase inhibitor (Roche) for 1 hour at 37 °C. RNA was purified a second time using TRIzol™ and the Direct-zol RNA miniprep kit.

To prepare logarithmic- and stationary-phase *Pst* Δ*hrcC*Δ*CFA* cultures for RNA extraction, bacteria were grown in liquid KB overnight at 30 °C. For logarithmic-phase samples, overnight bacterial suspensions were diluted with KB to 10^8 CFU mL^{-1} and grown for 6 hours. Stationary-phase samples were not diluted and grown for 6 additional hours.

To extract RNA from bacterial cultures grown *in vitro*, 10^9 CFUs of bacteria were resuspended in 150 µL of buffer TE (10 mM Tris-HCl, 1 mM EDTA, pH 8.0) with 5 mg mL^{-1} of lysozyme (Roche) for 5 minutes at room temperature. For the *in vitro* liquid-grown logarithmic- and stationary-phase bacteria, an extra step that used the RNA stabilizer RNAprotect Bacteria Reagent (QIAGEN) was used before the addition of lysozyme. After incubation with lysozyme, 400 µL of buffer RLT (QIAGEN) were added to the suspension, which was then applied through QIA shredder columns (QIAGEN). The eluate was used for RNA purification using the RNeasy mini kit (QIAGEN). The only modification to the protocol of the kit was the extension of the on column DNase I (QIAGEN) digestion to 30 minutes. After RNA purification, a second DNase I treatment and purification using the Direct-zol RNA miniprep kit was performed as described above.

To check for the presence of genomic DNA (gDNA) contamination in the RNA samples, a PCR using 50–100 ng of RNA was performed using primers AVL009 and AVL010 (to check for *Pst* gDNA

contamination), AVL011 and AVL012 (to check for *Pandoraea* sp. Col-0-28 gDNA contamination), AVL013 and AVL014 (to check for *A. xylosoxidans* sp. Col-0-50 gDNA contamination), and AVL015 and AVL016 (to check for *Arabidopsis thaliana* gDNA contamination; Table S12).

cDNA preparation and quantitative reverse transcription-PCR (qRT-PCR)

Three-hundred ng to two µg of RNA were used to prepare cDNA using 2.5 µM of random hexamer primers (Integrated DNA Technologies, Inc.), 40 U of Protector RNase inhibitor and 200 U of M-MLV reverse transcriptase (Invitrogen™), following the protocol described by the manufacturer. Between 0.4 and 1 µL of cDNA was used with appropriate primers (Table S12) and SYBR® Green PCR master mix (Applied Biosystems) for qRT-PCR. Two technical replicates were done per sample. PCR was performed on the 7500 Fast Real-Time PCR System (Applied Biosystems™) using automatic C_T threshold detection. Bacterial transcripts were normalized to the *leuB* (*PSPTO_2175*) reference gene using the ΔC_T method (10). In test RNA-Seq experiments, *leuB* showed very little variability in expression under multiple conditions. *rpoD*, one of our evaluated genes, has been used in the past as a qPCR reference gene (11); however, its expression, at least under certain conditions, is too variable for this purpose (Figures 6*B* and 6*C*). The efficiency of all primer pairs was validated to be between 90 to 110%. Amplicon melting curves had a single predominant peak.

For qRT-PCR of *Pst* DC3000 genes under ETI-inducing conditions (in Bu-22 plants; Figures 6*C*, S7*C* and S7*D*), we initially tried using a high bacterial inoculum, similar to the inoculum that had been used in previous *in planta* transcriptomic studies (1, 12). However, even though a faster hypersensitive response is observed at a high inoculum in Bu-22 plants (13), ETI restriction of bacterial multiplication is compromised (Figure S7*E*). As such, we decided to use a lower inoculum for evaluating gene expression in an incompatible interaction (Figure S7*F*). Samples for ETI-inducing *Pst* DC3000 were collected starting at 24 hours post-inoculation, once population density became static (Figure S7*F*).

RNA-Sequencing and bioinformatic analysis

RNA integrity was evaluated using the 2100 BioAnalyzer (Agilent Technologies, Inc.) and RNA concentration was determined with the Qubit™ RNA HS assay kit (Thermo Fisher Scientific). Libraries for RNA-Seq were prepared using the protocol described in Nobori *et al.* (1), but using the newest iteration of the library preparation kit from NuGEN® (Tecan Genomics, Inc.), the Universal RNA-Seq with NuQuant® kit, and skipping the use of the MICROBEnrich kit. This protocol allows the removal of plant and bacterial rRNAs and of the most abundant Arabidopsis transcripts. Libraries were pooled and sequenced in the Illumina® HiSeq 4000 using HiSeq 4000 SBS reagents to obtain single 50-bp reads. Base calling was done by Illumina Real Time Analysis (RTA version 2.7.7). RNA-Seq reads were deposited under BioProject PRJNA738276 of the Sequence Read Archive (SRA) (14).

The read quality was evaluated with FASTQC (version 0.11.7) (15), while adapters and low quality sequences were trimmed with Cutadapt (version 2.9) (16). Three different pipelines were used to calculate differential gene expression (DGE), as DGE varies depending on the models used for transcript estimation

(17). In the first pipeline, reads were aligned to the reference genomes using HISAT2 (version 2.1.0) (18). Aligned reads were processed using SAMTools (version 1.9) (19), after which StringTie (version 2.1.3) (20) was used to calculate transcript frequency, to finally estimate DGE using DESeq2 (average expression per treatment is shown on Datasets S1, S2, and S3) (21). On the second pipeline, reads were also aligned with HISAT2, but this time DGE was calculated directly using Cuffdiff (version 2.2.1) (17). In the third pipeline, reads were pseudo-aligned using Salmon (version 0.11.3 for *Pst ΔhrcCΔCFA* or 1.2.1 for the other two endophytes) (22), after which DESeq2 was performed for DGE. Differentially expressed genes present in at least one of the three analyses were used for subsequent analysis. Comparison of the three pipelines for each endophyte is shown in Tables S13, S14 and S15.

Principal component analysis (PCA) of transcript reads after DESeq2 analysis used the rlog and plotPCA functions of DESeq2 in Rstudio (version 1.3.1093). Significantly up- or down-regulated genes were identified as those in any of the three pipelines in which the absolute value of the logarithm (in base 2) of the fold change was greater than 0.58, and the adjusted false discovery rate *p* value of the statistical test was less than 0.05. GO (gene ontology) enrichment analysis used the Cytoscape application BINGO (version 3.0.4) (23). For *Pst ΔhrcCΔCFA*, we used the default database incorporated into the bioinformatics tool, while for *A. xylosoxidans* and *Pandoraea*, we created reference databases using InterProScan (version 5.47-82.0). GO results were summarized with REVIGO to remove GO term redundancies (24), after which manual curation of the enriched pathways was done. A complete list of biological pathways enriched in differentially expressed up- or down-regulated genes is shown in Tables S1, S2, and S3. PaintOmics 3 (version 0.4.5) (25) was used for initial visualization of *Pst ΔhrcCΔCFA* gene expression in biological pathways. We used antiSMASH (version 6.0.0) (26) to identify clusters of genes predicted to be involved in the production of secondary metabolites.

STEM (Short-Time series Expression Miner; version 1.3.13) (27) analysis was used to look for enriched patterns of expression over time between the samples collected at 0 (the inoculum), 6, 24, and 168 hours post-inoculation (hpi) into Col-0 plants, to identify sets of genes whose expression increased or decreased once phyllosphere bacteria were inside plants. Analysis used the logarithmically (in base 2) normalized expression data obtained from StringTie. GO pathway enrichment analysis used the BINGO application, and summarization of the results was done as described above (Table S4, S5, and S6).

Orthologous gene groups between bacterial strains were identified using OrthoMCL (28). Each orthologous group may have more than one gene from each strain, and were identified as having an expected value of less than 10^{-5}. Genes that had the same pattern of differential up- or down-regulation for a comparison between two treatments in one, two or all three strains were identified (Figure S8). We only compared the inoculum or 6 hpi with 168 hpi, as we expected these two comparisons to be the most significant for the long-term adaptation of phyllosphere microbiota to plants. Biological process enrichment analysis of orthologous groups used the *Pst ΔhrcCΔCFA* genes as references.

Plant-associated genes (PA) were retrieved from Levy *et al.* (29) using the PA protein sequences provided. *Pst* DC3000 PA proteins were directly retrieved from such publication. To identify PA proteins in *Achromobacter xylosoxidans* Col-0-50, proteins from 11 strains that belonged to the genus *Achromobacter*

(29) were used to identify PA proteins in *A. xylosoxidans* Col-0-50, using the program CD-HIT (version 4.8.1) (30). The same program was used to identify PA proteins in *Pandoraea* sp. Col-0-28, this time using the PA proteins from 5 *Pandoraea* strains, plus three closely related strains isolated from plants from the genus *Burkholderia* (*Burkholderia andropogonis* Ba3549, *Burkholderia* sp. JPY347, and *Burkholderia* sp. YR520) (29). Proteins were considered orthologous if they had at least 80% amino acid identity and coverage with a protein from Levy *et al.* (29). PA gene enrichment was calculated using a hypergeometric test and a Bonferroni-Hochberg correction (p < 0.05).

The reference genomes used for analysis are those from *Pst* DC3000: GenBank accession AE016853.1 for the genomic DNA, and GenBank accessions AE016855.1 and AE016854.1 for the two plasmids. For *A. xylosoxidans* Col-0-50 and *Pandoraea* sp. Col-0-28, the genome sequences are under the BioProject PRJNA555902 deposited in the SRA (sample runs SRR9732406 and SRR9732397, respectively).

Graphs and statistical analysis were performed using Prism (version 6.0b) or Rstudio (Bubble graphs and Venn diagrams). Upset plots and the heatmaps for Figures 4*D* and 4*E* were graphed using TBTools (version 1.0) (31). Figure 6*D* and part of Figure S5 were created with BioRender.com.

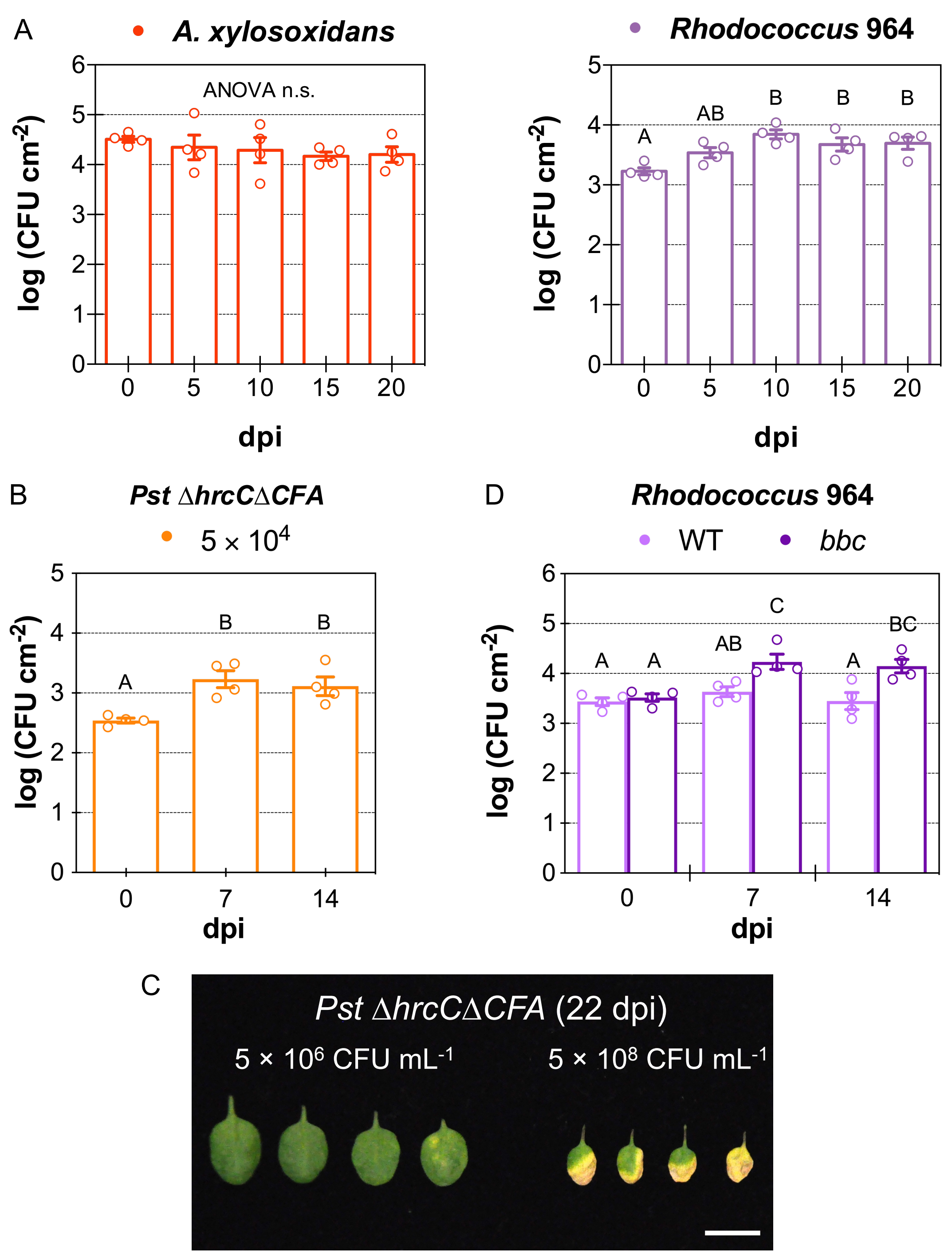

Fig. S1. Endophytic bacterial populations experience stasis inside plants. (*A*) Bacterial population density of gram-negative and gram-positive microbiota members *Achromobacter xylosoxidans* Col-0-50 (inoculum: 10^7 CFU mL^{-1}) and *Rhodococcus* sp. 964 (inoculum: 10^6 CFU mL^{-1}) over the course of 20 days after infection of Col-0 plants. (*B*) Bacterial population density of *Pst* Δ*hrcC*Δ*CFA* inoculated at 5×10^4 CFU mL^{-1} into Arabidopsis leaves. (*C*) Photographs of Col-0 leaves inoculated with non-pathogenic *Pseudomonas syringae* pv. *tomato* (*Pst*) Δ*hrcC*Δ*CFA* at two different inoculum levels 22 days after infiltration. Leaves infiltrated at the higher inoculum were chlorotic and barely grew over the course of 22 days. Scale bar represents 1 cm. (*D*) Population density of *Rhodococcus* sp. 964 (inoculum: 5×10^5 CFU mL^{-1}) over the course of 2 weeks after infiltration into leaves of Col-0 and PTI-compromised triple mutant *bak1 bkk1 cerk1* (*bbc*).

Individual biological repetitions for each treatment are shown as open circles. Error bars indicate the standard error of the mean. When appropriate, different letters indicate differences in means, as judged by a Tukey HSD test ($p < 0.05$). dpi indicates days post-inoculation.

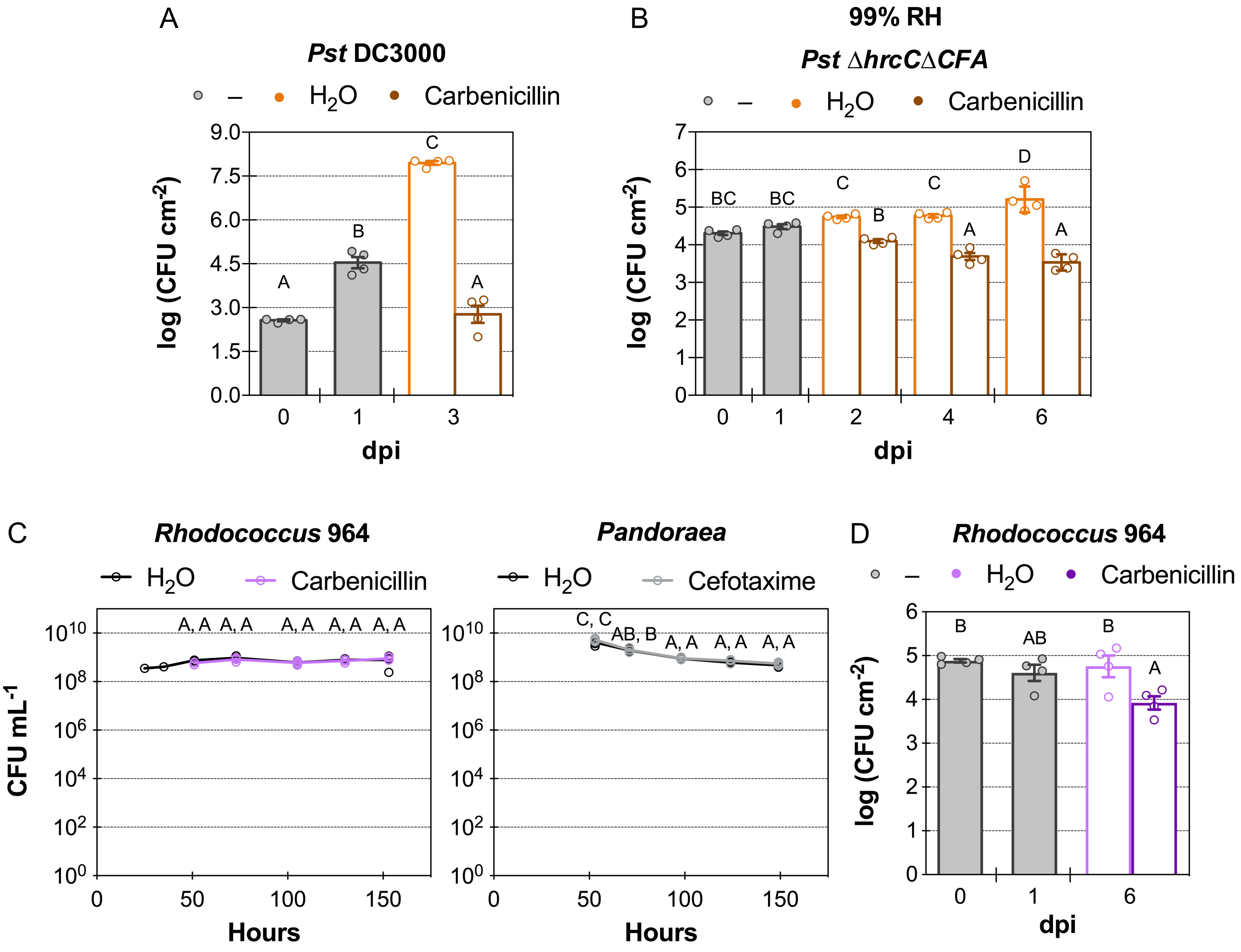

Fig. S2. Equilibrium in bacterial multiplication and death underlies bacterial population stasis. (*A*) Bacterial population density of virulent *Pseudomonas syringae* pv. *tomato* (*Pst*) DC3000 (inoculum: 10^5 CFU mL^{-1}) in Col-0 plants after infiltration with 400 μg mL^{-1} of carbenicillin, an antibiotic that kills dividing bacteria. Note the dramatic reduction in population density after carbenicillin treatment to 0.001% of the water control. (*B*) Same experiment as in Fig. 2*B*, but, using a relative humidity of over 99% throughout the experiment. *Pst ΔhrcCΔCFA* was inoculated at 10^7 CFU mL^{-1}, and 400 μg mL^{-1} of carbenicillin was infiltrated daily starting at 24 hours post-inoculation. Over 98% of the population is killed over the course of 5 days. (*C*) *In vitro* β-lactam antibiotic effect of 400 μg mL^{-1} of carbenicillin or cefotaxime on stationary-phase *Rhodococcus* sp. 964 and *Pandoraea* sp. Col-0-28. The y-axis is in logarithmic scale. (*D*) *In planta* population density of *Rhodococcus* sp. 964 (inoculum: 2 × 10^7 CFU mL^{-1}) after addition of 400 μg mL^{-1} of carbenicillin. Over 87% of the population was killed after 5 days.

Individual biological repetitions for each treatment are shown as circles. Error bars indicate the standard error of the mean. Different letters indicate differences in means, as judged by a Tukey HSD test ($p < 0.05$). dpi indicates days post-inoculation.

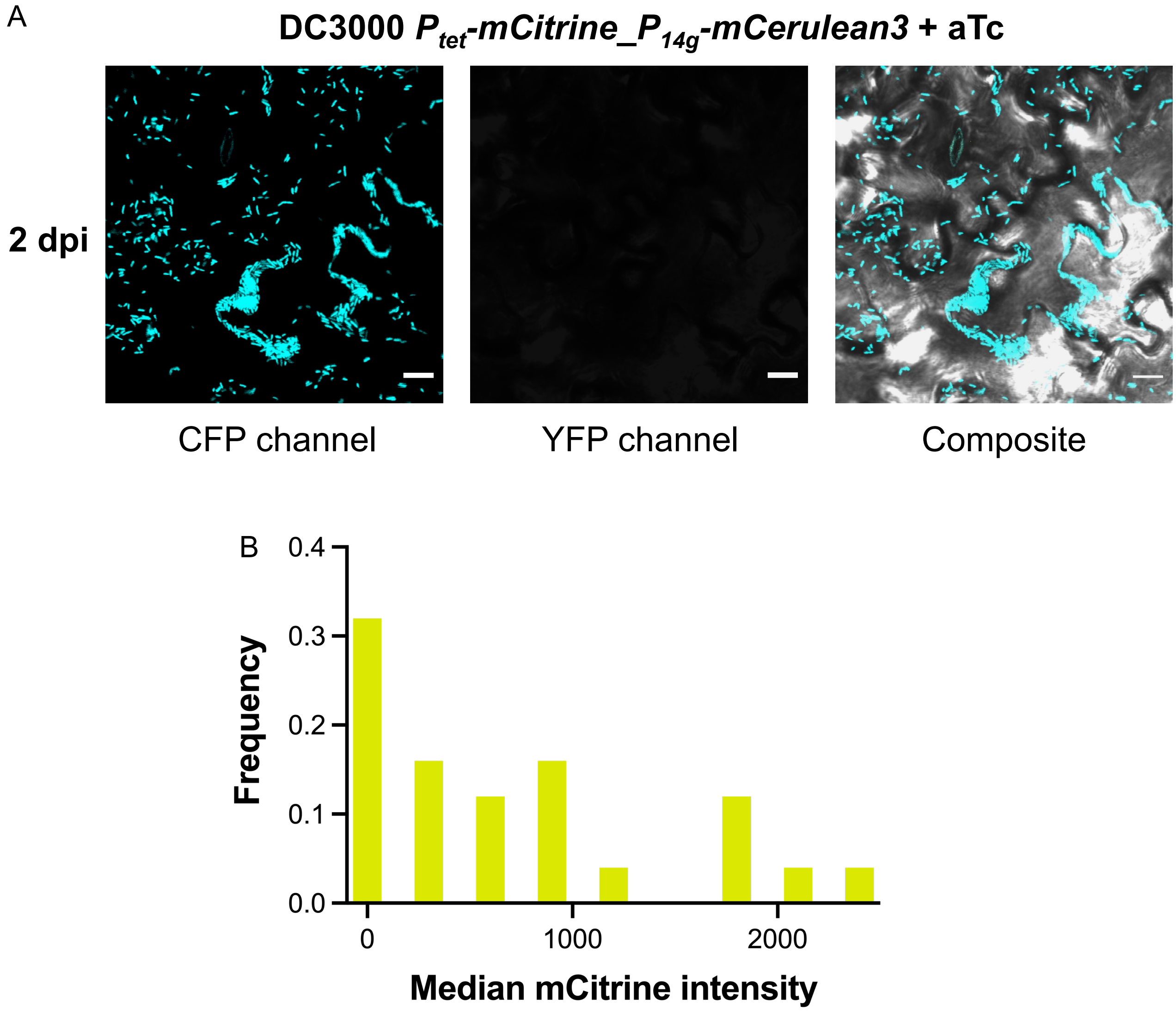

Fig. S3. *In planta* visualization of bacterial endophytes. (*A*) Visualization of pathogenic *Pseudomonas syringae* (*Pst*) carrying the fluorescent division reporter on the surface of Arabidopsis leaves. Confocal images of virulent *Pst* DC3000 carrying the P_{tet}-*mCitrine*_P_{14g}-*mCerulean3* division reporter (inoculum: 5×10^7 CFU mL^{-1}; grown with 80 ng mL^{-1} anhydrotetracycline [aTc]) 2 days after inoculation of Col-0 plants. Images were taken at the leaf surface, where individual fluorescent bacteria can be observed. Note that no mCitrine signal is observed in the samples, as it was diluted out during *in planta* bacterial multiplication. Cyan represents signal from mCerulean3. Yellow represents signal from mCitrine. Composite image of both fluorescent proteins and the transmitted light image is shown. Scale bars represent 10 μm. (*B*) Relative frequency of median mCitrine signal intensity (in arbitrary units) in areas in which there was detection of constitutive mCerulean3 signal from endophytic *Pst* Δ*hrcC*Δ*CFA* carrying the division reporter P_{tet}-*mCitrine*_P_{14g}-*mCerulean3* after 5 days post-inoculation into Arabidopsis leaves. Quantification was performed on the confocal image presented on Fig. 3*D*.

Fig. S4

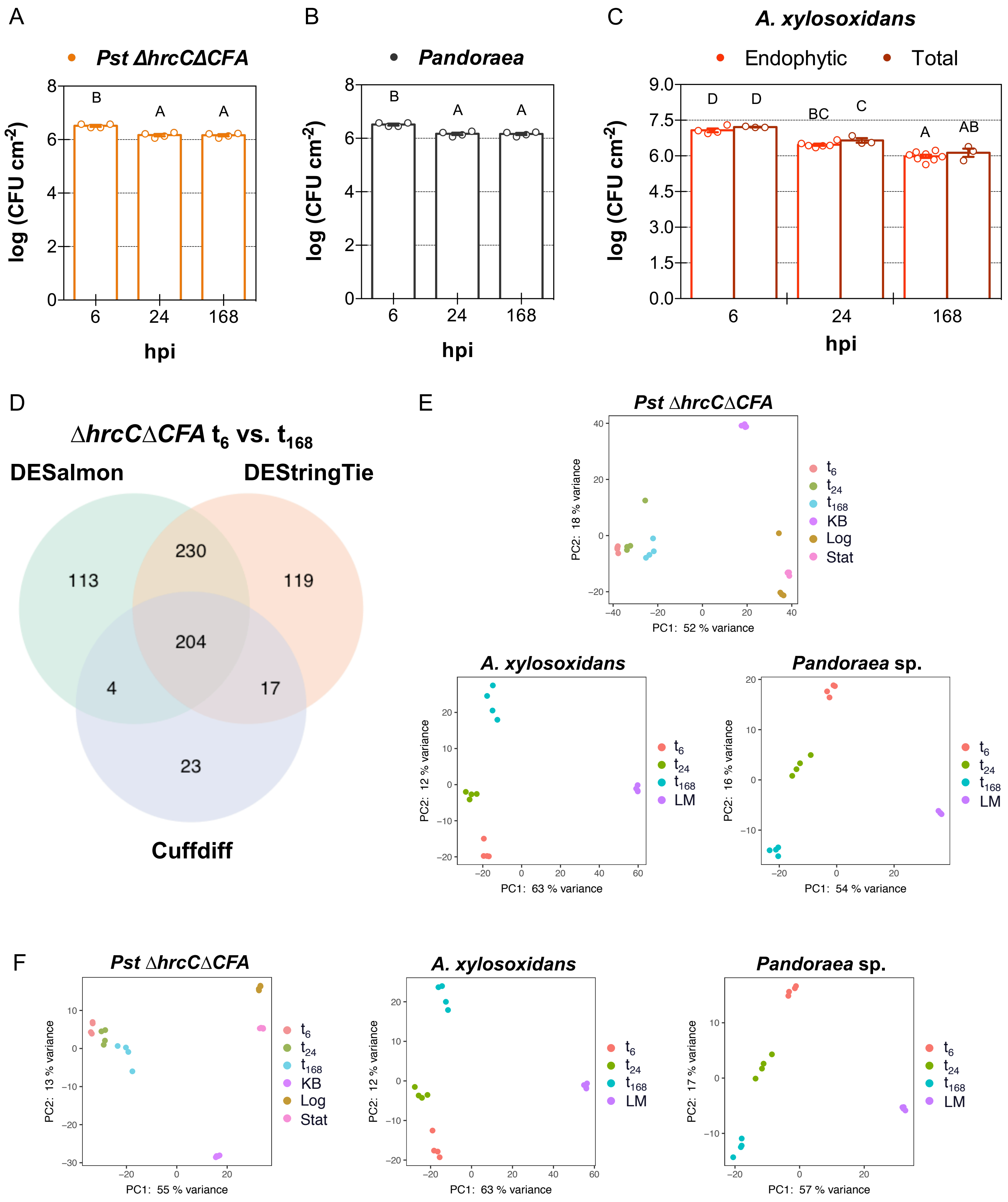
A
Pst ΔhrcCΔCFA
log (CFU cm-2)
hpi
B
Pandoraea
C
A. xylosoxidans
Endophytic
Total
D
ΔhrcCΔCFA t6 vs. t168
DESalmon
DEStringTie
Cuffdiff
113
230
119
204
4
17
23
E
Pst ΔhrcCΔCFA
A. xylosoxidans
Pandoraea sp.
PC1: 52 % variance
PC2: 18 % variance
PC1: 63 % variance
PC2: 12 % variance
PC1: 54 % variance
PC2: 16 % variance
t6
t24
t168
KB
Log
Stat
LM
F
PC1: 55 % variance
PC2: 13 % variance
PC1: 57 % variance
PC2: 17 % variance

Fig. S4

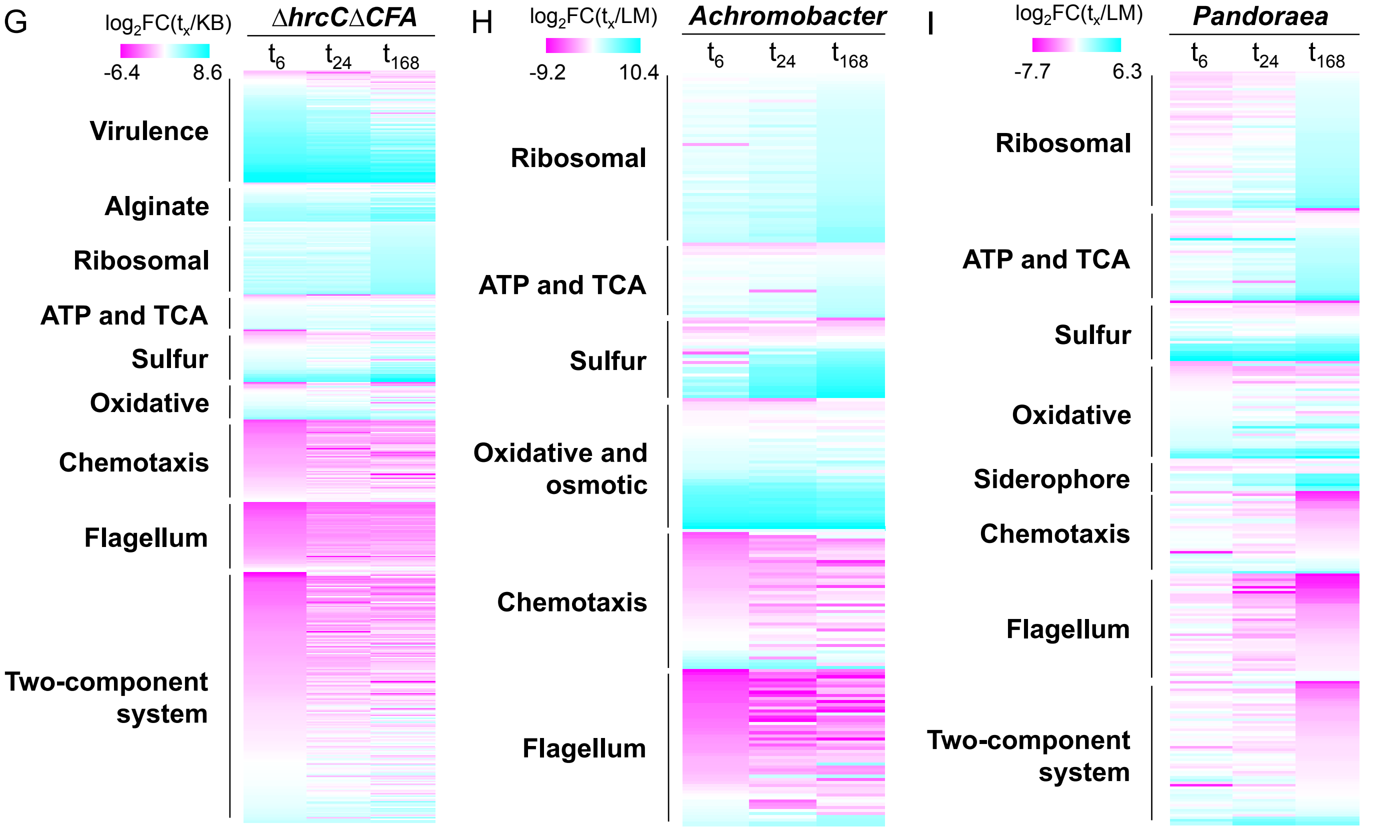

Fig. S4. Transcriptome and population analysis of three phyllosphere-inhabiting bacteria. (*A*) Bacterial population density of non-pathogenic *Pseudomonas syringae* pv. *tomato* (*Pst*) Δ*hrcC*Δ*CFA* mutant (inoculum: 2 × 10^9 CFU mL^{-1}) over the course of 7 days in Col-0 plants. These samples were used for the bacterial RNA-Seq analysis presented in Fig. S4*G*. (*B*) Bacterial population density of *Pandoraea* sp. Col-0-28 (inoculum: 2 × 10^9 CFU mL^{-1}) over the course of 7 days in Col-0 plants. These samples were used for the bacterial RNA-Seq analysis presented in Fig. S4*H*. (*C*) Total and endophytic bacterial population density of *Achromobacter xylosoxidans* Col-0-50 (inoculum: 2 × 10^9 CFU mL^{-1}) over the course of 7 days in Col-0 plants. Endophytic samples were used for the bacterial RNA-Seq analysis presented in Fig. S4*I*. (*D*) Venn diagram showing the number of differentially expressed *Pst* Δ*hrcC*Δ*CFA* genes between 6 (t_6) and 168 (t_{168}) hpi into Arabidopsis leaves after using one of three methods: DESeq2 after pseudo-alignment with Salmon (DESalmon), DESeq2 after alignment with HISAT2 and analysis with StringTie (DEStringTie), and Cuffdiff after alignment with HISAT2 (Cuffdiff). (*E*) Principal component analysis (PCA) of transcript reads pseudo-aligned with Salmon, and then analyzed with DESeq2 with the rlog and plotPCA functions in R. There were three experiments: Col-0 plants were inoculated with *Pst* Δ*hrcC*Δ*CFA*, *A. xylosoxidans* Col-0-50, or *Pandoraea* sp. Col-0-28 at 6 (t_6), 24 (t_{24}), and 168 (t_{168}, 7 days) hpi, while their respective inoculum was grown in King's (KB) or modified LB (LM) medium. *In vitro* logarithmic (Log) or stationary (Stat) phase treatments were also included for *Pst* Δ*hrcC*Δ*CFA*. (*F*) PCA of transcript reads aligned with HISAT2, and then analyzed with StringTie and DESeq2. Same experiments as in Fig. S4*E* were analyzed. (*G*) Long-term *in planta* transcriptome heatmap of gene expression of select enriched biological processes in non-pathogenic *Pst* Δ*hrcC*Δ*CFA* and microbiota strains (*H*) *Achromobacter xylosoxidans* Col-0-50 and (*I*) *Pandoraea* sp. Col-0-28. Gene expression values of select categories were graphed in ascending order of their log_2 fold change (FC) of the ratio between 6 (t_6), 24 (t_{24}) or 168 (t_{168}) hpi and the expression in the inoculum (KB or LM). FC was color coded for repression (magenta), no change (white), and induction (cyan). Individual biological repetitions for each treatment are shown as circles. Error bars indicate the standard error of the mean. Different letters indicate differences in means, as judged by a Tukey HSD test ($p < 0.05$). hpi indicates hours post-inoculation. TCA = tricarboxylic acid cycle.

Fig. S5

A

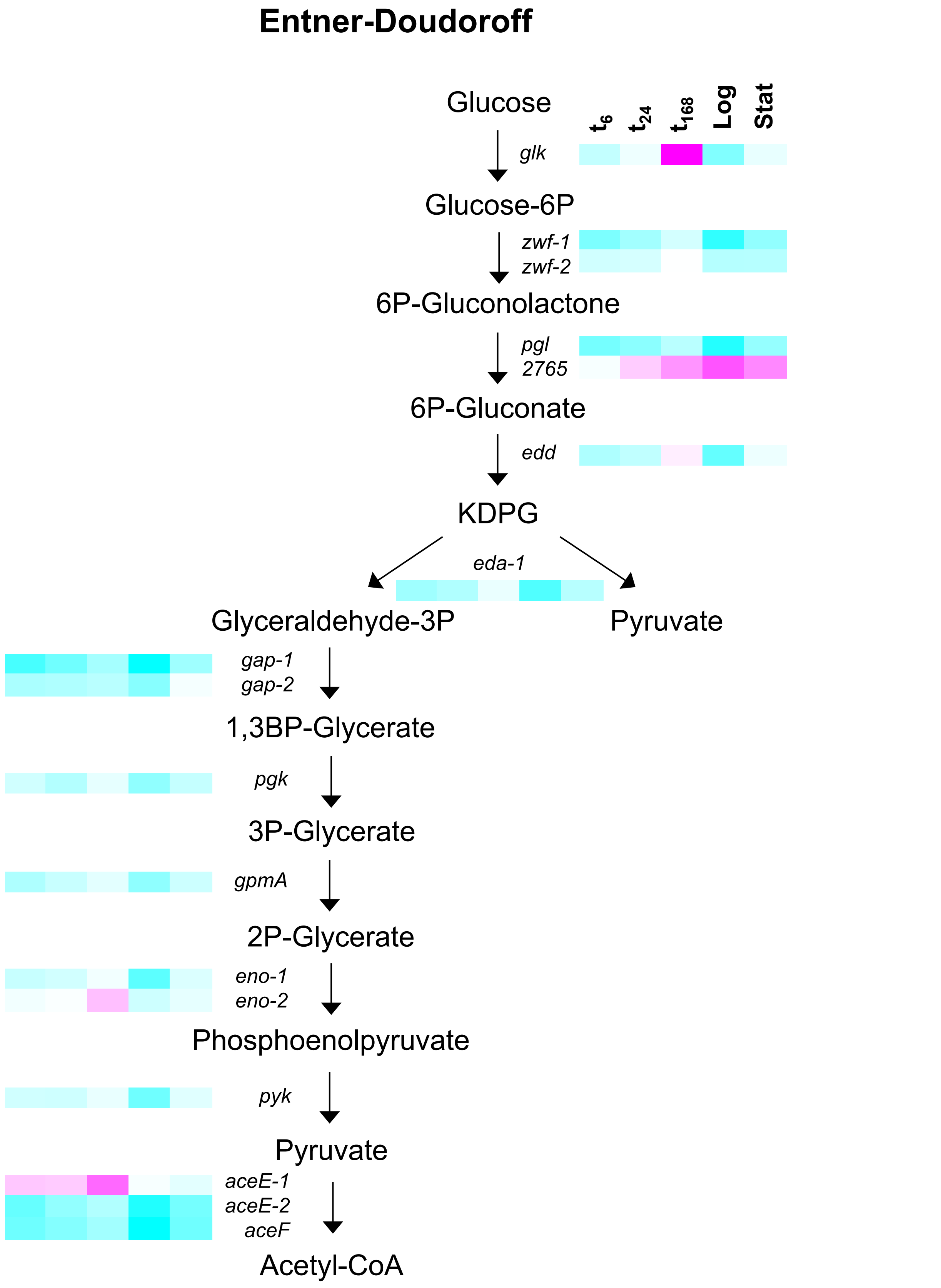
Entner-Doudoroff
Glucose
t_6
t_{24}
t_{168}
Log
Stat
glk
Glucose-6P
zwf-1
zwf-2
6P-Gluconolactone
pgl
2765
6P-Gluconate
edd
KDPG
eda-1
Glyceraldehyde-3P
Pyruvate
gap-1
gap-2
1,3BP-Glycerate
pgk
3P-Glycerate
gpmA
2P-Glycerate
eno-1
eno-2
Phosphoenolpyruvate
pyk
Pyruvate
aceE-1
aceE-2
aceF
Acetyl-CoA
$log_2FC(t_x/KB)$
-2.1
6.2

Fig. S5

B

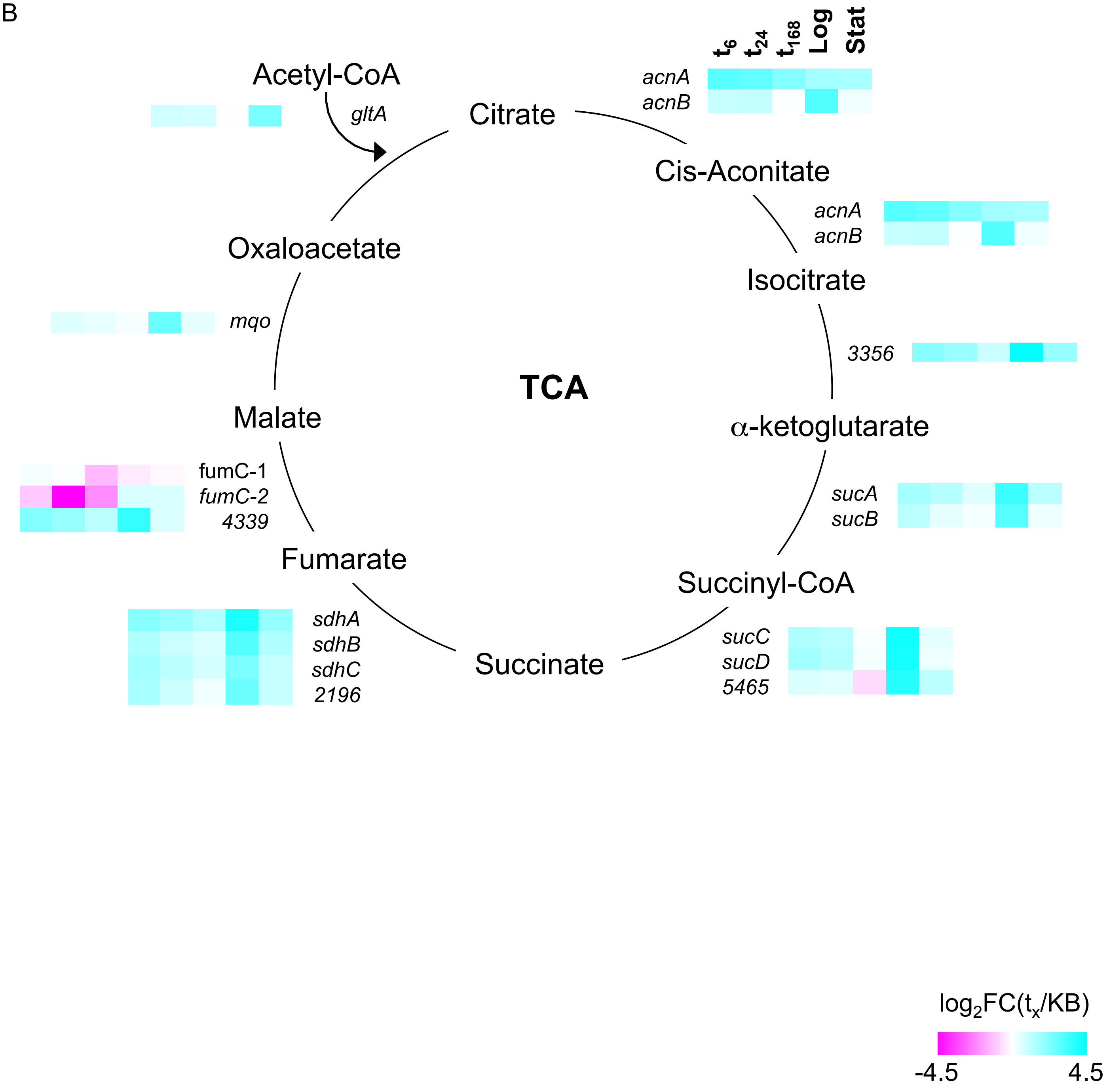
t6
t24
t168
Log
Stat
Acetyl-CoA
gltA
Citrate
acnA
acnB
Cis-Aconitate
acnA
acnB
Isocitrate
3356
α-ketoglutarate
sucA
sucB
Succinyl-CoA
sucC
sucD
5465
Succinate
sdhA
sdhB
sdhC
2196
Fumarate
fumC-1
fumC-2
4339
Malate
mqo
Oxaloacetate
TCA
log2FC(tx/KB)
-4.5
4.5

Fig. S5

C

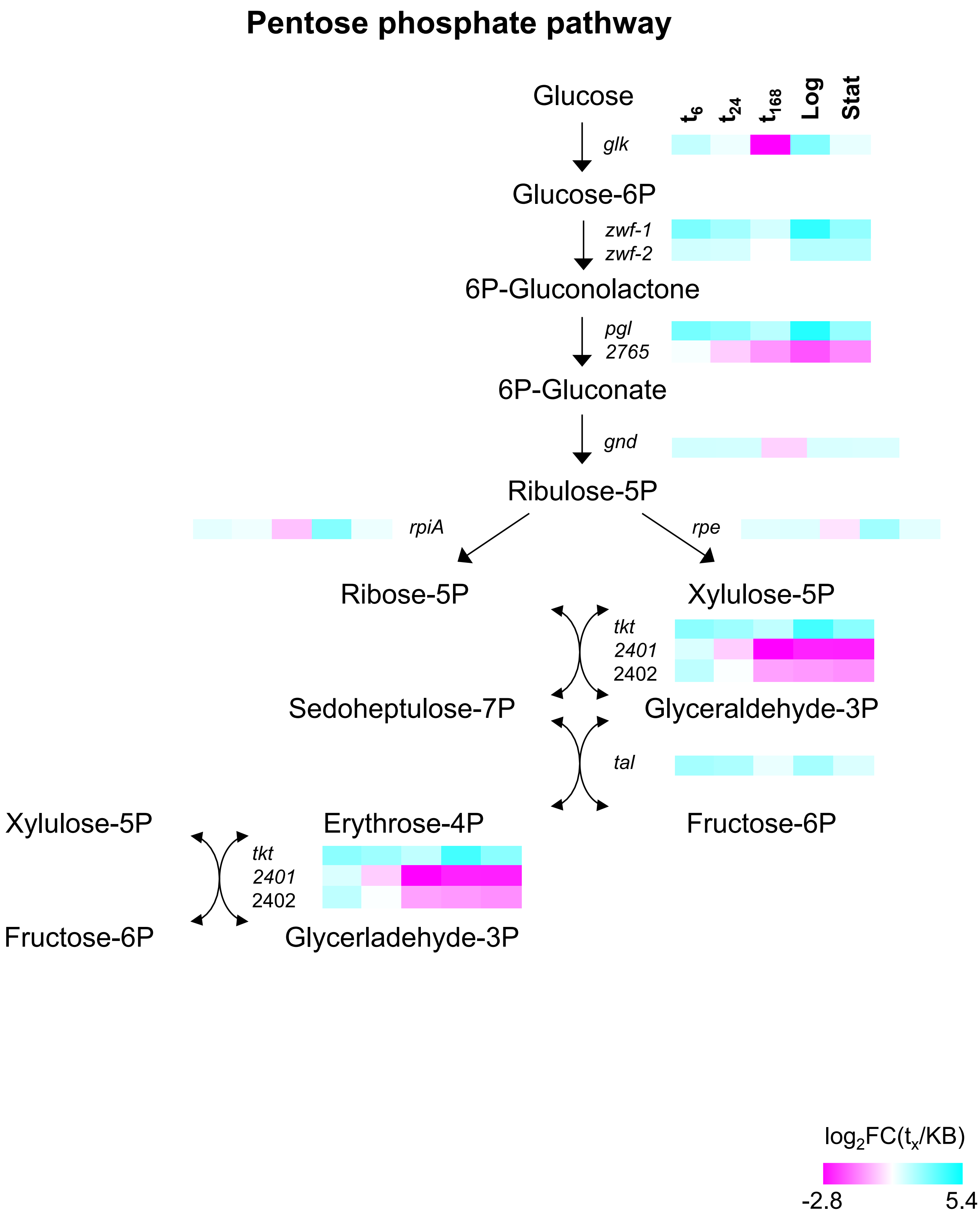

Pentose phosphate pathway
t6
t24
t168
Log
Stat
Glucose
glk
Glucose-6P
zwf-1
zwf-2
6P-Gluconolactone
pgl
2765
6P-Gluconate
gnd
Ribulose-5P
rpiA
rpe
Ribose-5P
Xylulose-5P
tkt
2401
2402
Sedoheptulose-7P
Glyceraldehyde-3P
tal
Xylulose-5P
Erythrose-4P
Fructose-6P
tkt
2401
2402
Fructose-6P
Glycerladehyde-3P
log2FC(tx/KB)
-2.8
5.4

Fig. S5

D

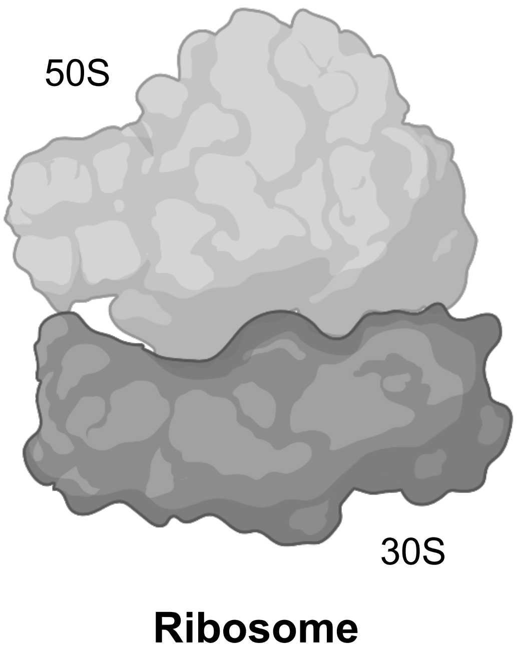
50S
30S
Ribosome

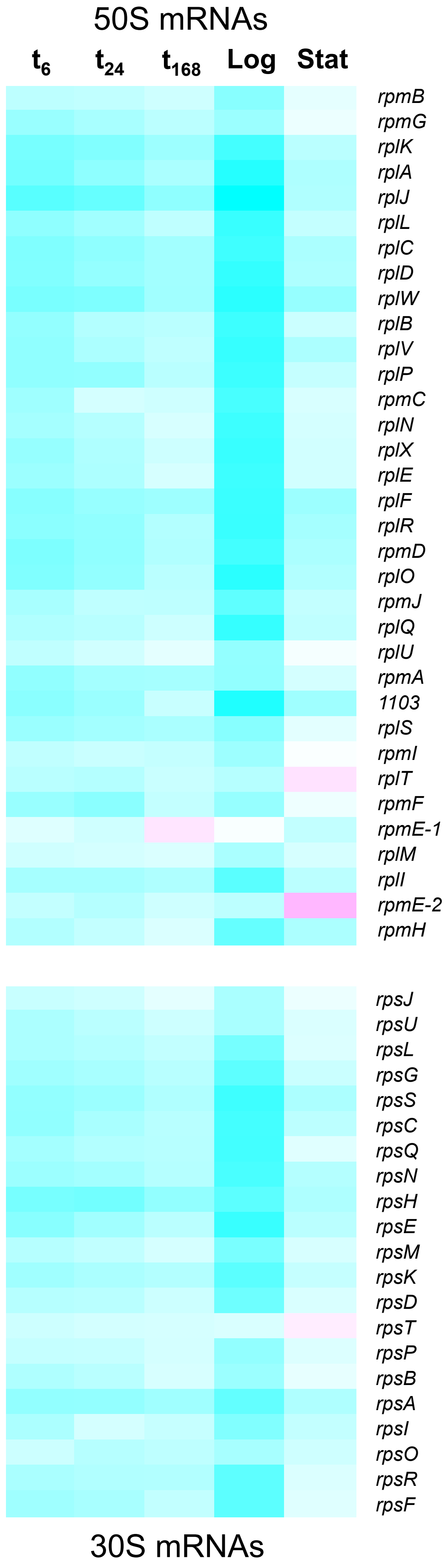
50S mRNAs
t6
t24
t168
Log
Stat
rpmB
rpmG
rplK
rplA
rplJ
rplL
rplC
rplD
rplW
rplB
rplV
rplP
rpmC
rplN
rplX
rplE
rplF
rplR
rpmD
rplO
rpmJ
rplQ
rplU
rpmA
1103
rplS
rpmI
rplT
rpmF
rpmE-1
rplM
rplI
rpmE-2
rpmH
rpsJ
rpsU
rpsL
rpsG
rpsS
rpsC
rpsQ
rpsN
rpsH
rpsE
rpsM
rpsK
rpsD
rpsT
rpsP
rpsB
rpsA
rpsI
rpsO
rpsR
rpsF
30S mRNAs
log2FC(tx/KB)
-2.7
6.8

Fig. S5

E

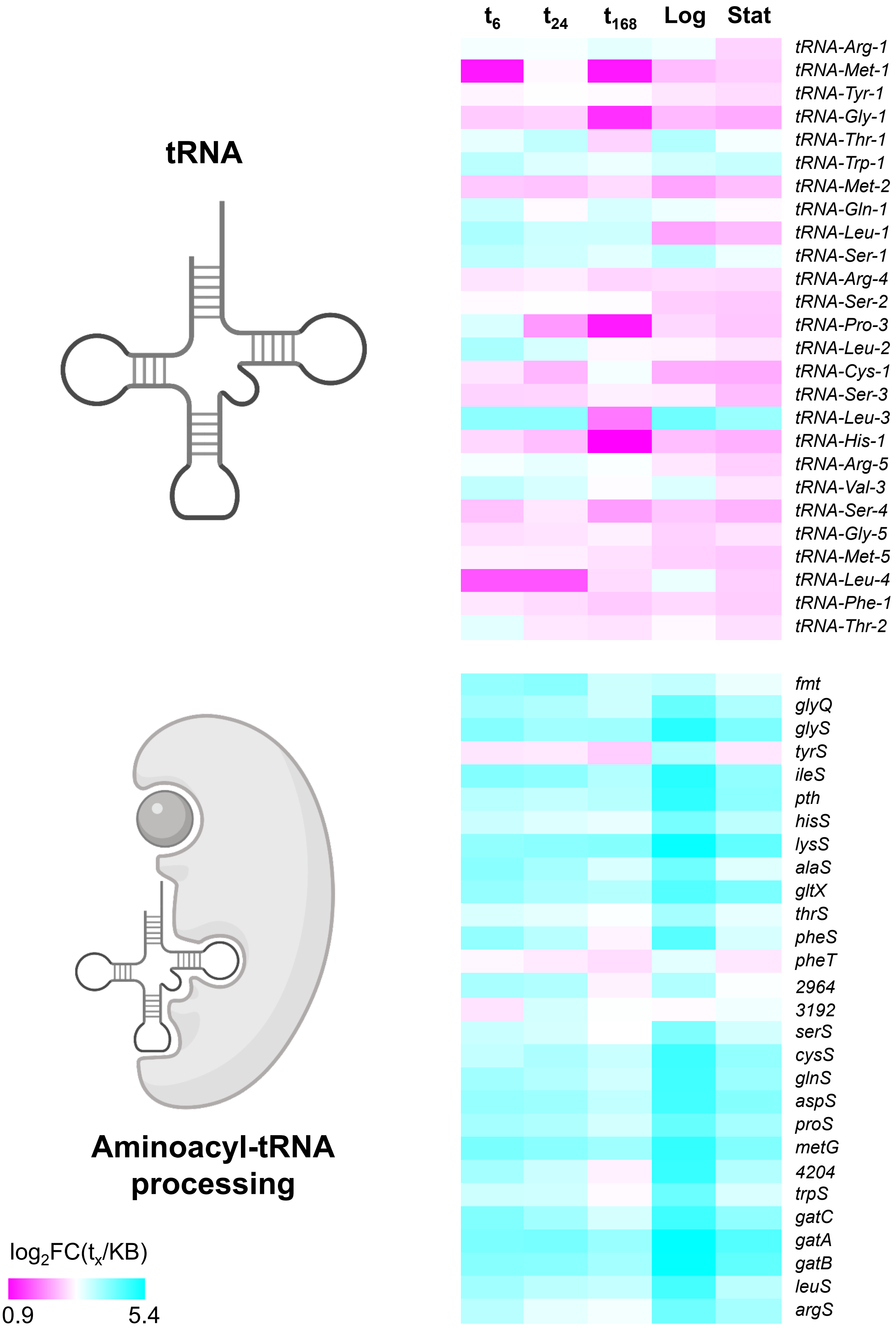
tRNA
Aminoacyl-tRNA processing
log2FC(tx/KB)
-10.9
5.4
t6
t24
t168
Log
Stat
tRNA-Arg-1
tRNA-Met-1
tRNA-Tyr-1
tRNA-Gly-1
tRNA-Thr-1
tRNA-Trp-1
tRNA-Met-2
tRNA-Gln-1
tRNA-Leu-1
tRNA-Ser-1
tRNA-Arg-4
tRNA-Ser-2
tRNA-Pro-3
tRNA-Leu-2
tRNA-Cys-1
tRNA-Ser-3
tRNA-Leu-3
tRNA-His-1
tRNA-Arg-5
tRNA-Val-3
tRNA-Ser-4
tRNA-Gly-5
tRNA-Met-5
tRNA-Leu-4
tRNA-Phe-1
tRNA-Thr-2
fmt
glyQ
glyS
tyrS
ileS
pth
hisS
lysS
alaS
gltX
thrS
pheS
pheT
2964
3192
serS
cysS
glnS
aspS
proS
metG
4204
trpS
gatC
gatA
gatB
leuS
argS

Fig. S5

F

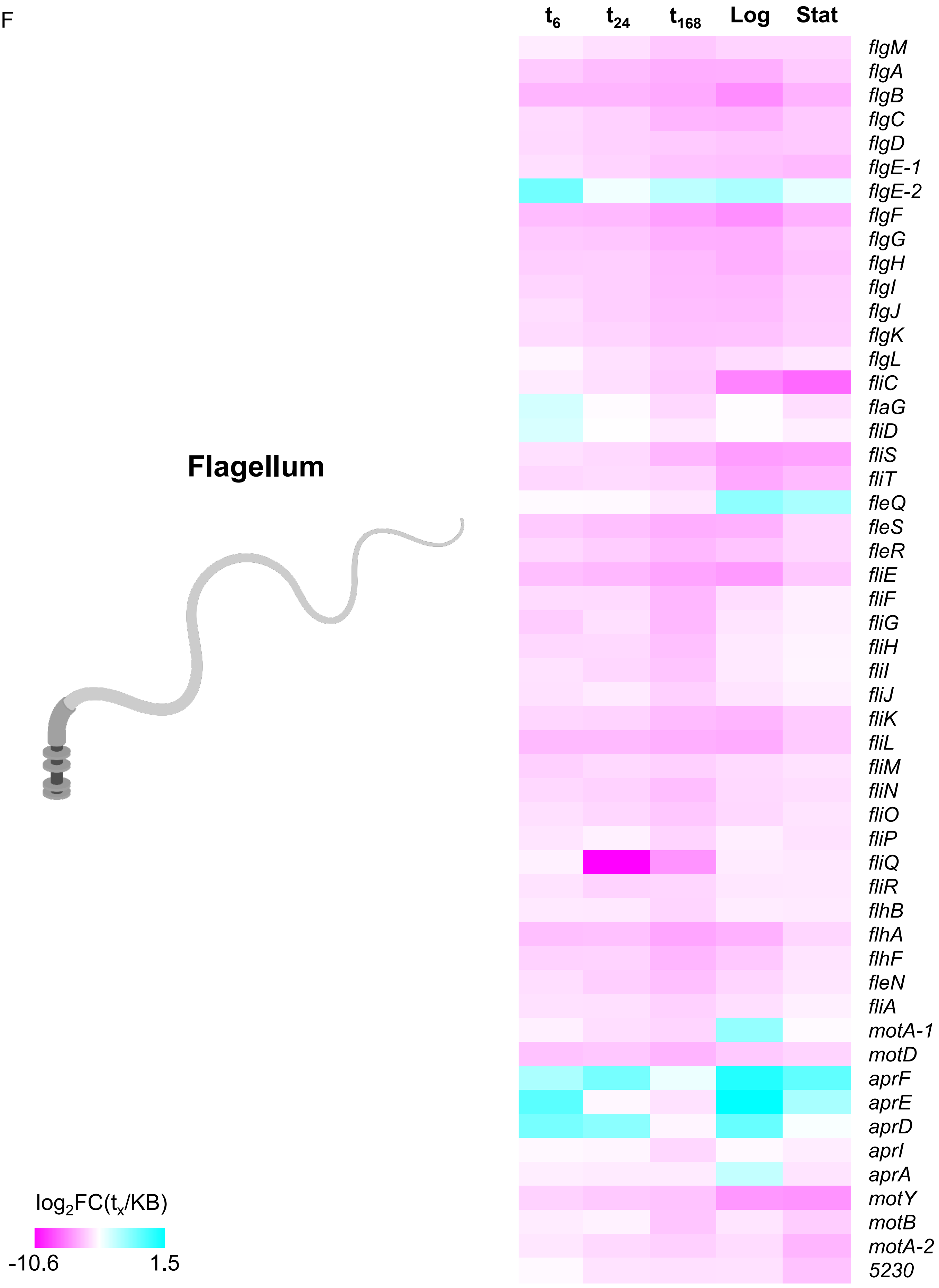

t6
t24
t168
Log
Stat
flgM
flgA
flgB
flgC
flgD
flgE-1
flgE-2
flgF
flgG
flgH
flgI
flgJ
flgK
flgL
fliC
flaG
fliD
fliS
fliT
fleQ
fleS
fleR
fliE
fliF
fliG
fliH
fliI
fliJ
fliK
fliL
fliM
fliN
fliO
fliP
fliQ
fliR
flhB
flhA
flhF
fleN
fliA
motA-1
motD
aprF
aprE
aprD
aprI
aprA
motY
motB
motA-2
5230
Flagellum
log2FC(tx/KB)
-10.6
1.5

Fig. S5

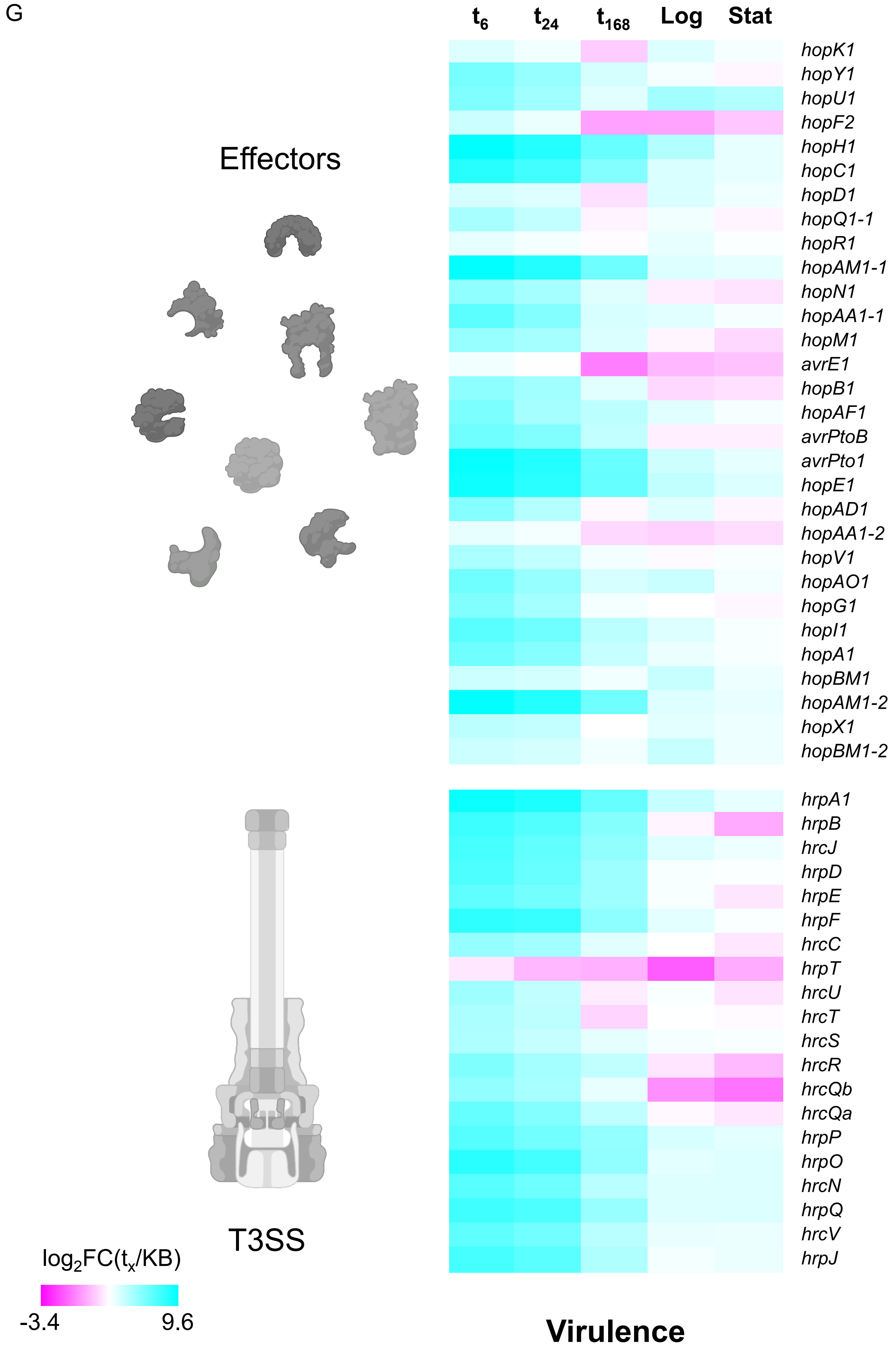
G
Effectors
T3SS
log2FC(tx/KB)
-3.4
9.6
t6
t24
t168
Log
Stat
hopK1
hopY1
hopU1
hopF2
hopH1
hopC1
hopD1
hopQ1-1
hopR1
hopAM1-1
hopN1
hopAA1-1
hopM1
avrE1
hopB1
hopAF1
avrPtoB
avrPto1
hopE1
hopAD1
hopAA1-2
hopV1
hopAO1
hopG1
hopI1
hopA1
hopBM1
hopAM1-2
hopX1
hopBM1-2
hrpA1
hrpB
hrcJ
hrpD
hrpE
hrpF
hrcC
hrpT
hrcU
hrcT
hrcS
hrcR
hrcQb
hrcQa
hrpP
hrpO
hrcN
hrpQ
hrcV
hrpJ
Virulence

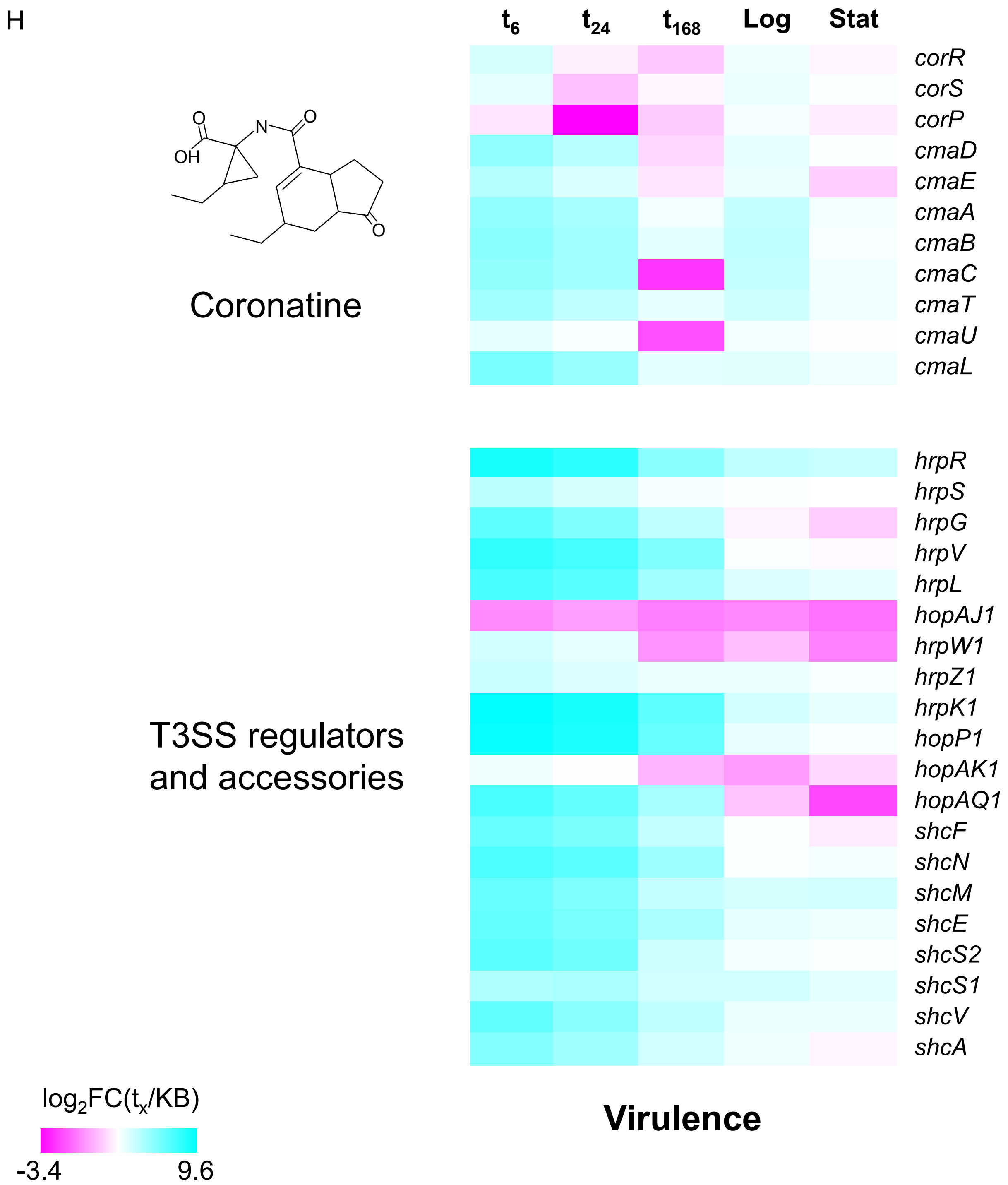

Fig. S5. Expression of genes from select biological pathways for *Pseudomonas syringae pv. tomato* Δ*hrcC*Δ*CFA*. Pathways represented were (*A*) the Entner-Doudoroff pathway (with the addition in the pathway of the conversion of pyruvate to acetyl-CoA), (*B*) the tricarboxylic acid cycle (TCA), (*C*) the pentose phosphate pathway, (*D*) ribosome biogenesis genes, (*E*) tRNAs and genes involved in tRNA aminoacylation, (*F*) flagellar biosynthesis genes, and (*G*) and (*H*) virulence-promoting genes encoding structural and regulatory components of the type III secretion system (T3SS), effectors, and coronatine biosynthesis genes.

Expression of each gene was calculated as the average $\log_2$ fold change (FC) of the ratio between the treatment and the inoculum. Expression of each gene is represented by five rectangles corresponding to each treatment in the following order: 6, 24, and 168 hours post-inoculation, and *in vitro* logarithmic and stationary phase populations. FC was color coded for repression (magenta), no change (white), and induction (cyan). When locus names are shown, "*PSPTO_*" is omitted from the name.

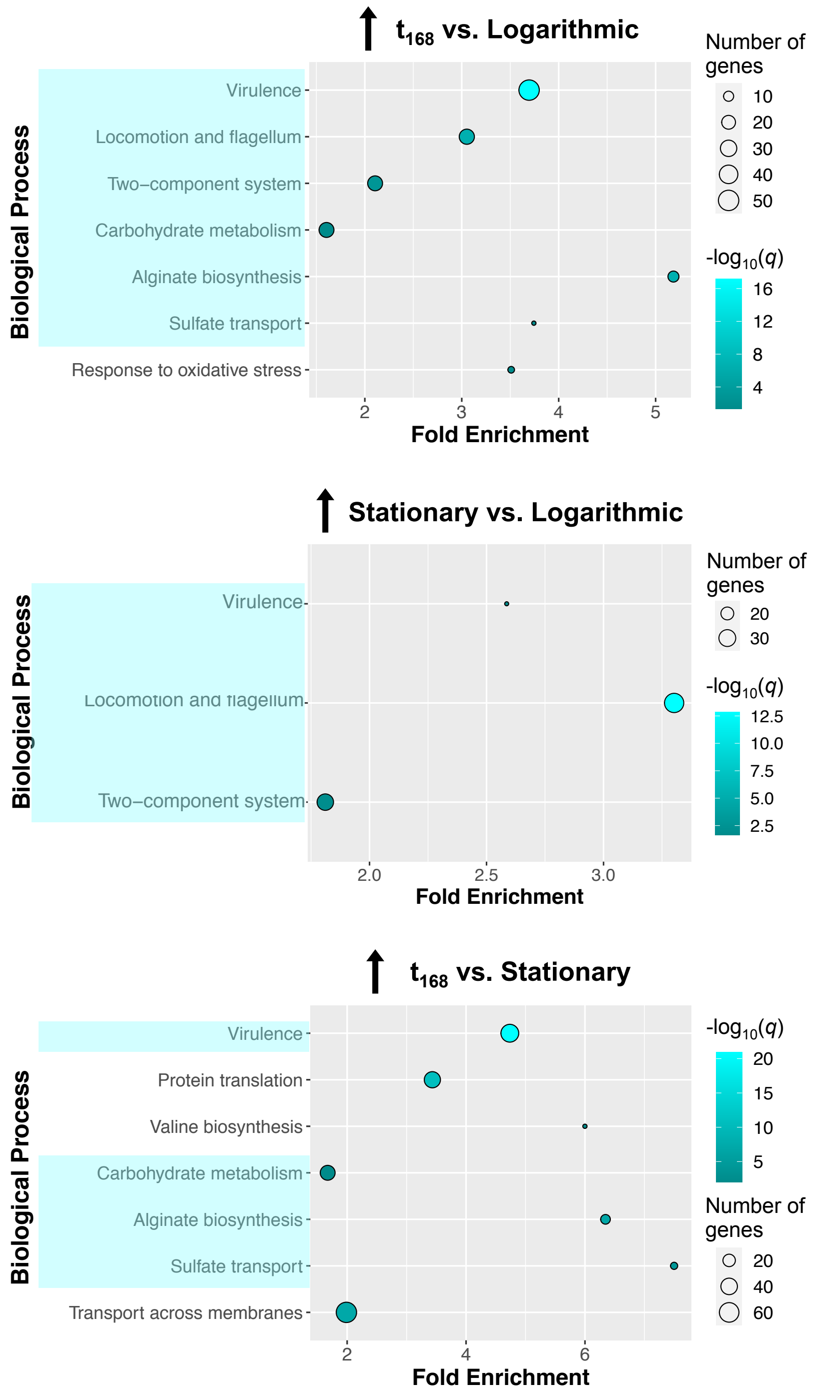

Fig. S6. Enriched biological processes in up-regulated differentially expressed genes (DEGs). Bubble graphs compared 168 hours post-inoculation (hpi; t_{168}) or *in vitro* stationary-phase populations with *in vitro* logarithmic-phase populations, or t_{168} with stationary-phase. Processes found in at least two comparisons are highlighted in light blue. The fold-enrichment, number of significant DEGs, and the $\log_{10}$ of the adjusted *p*-value (*q*) are shown for each biological process. Only processes with at least 4 genes are shown. Refers to Fig. 5*B*.

Fig. S7

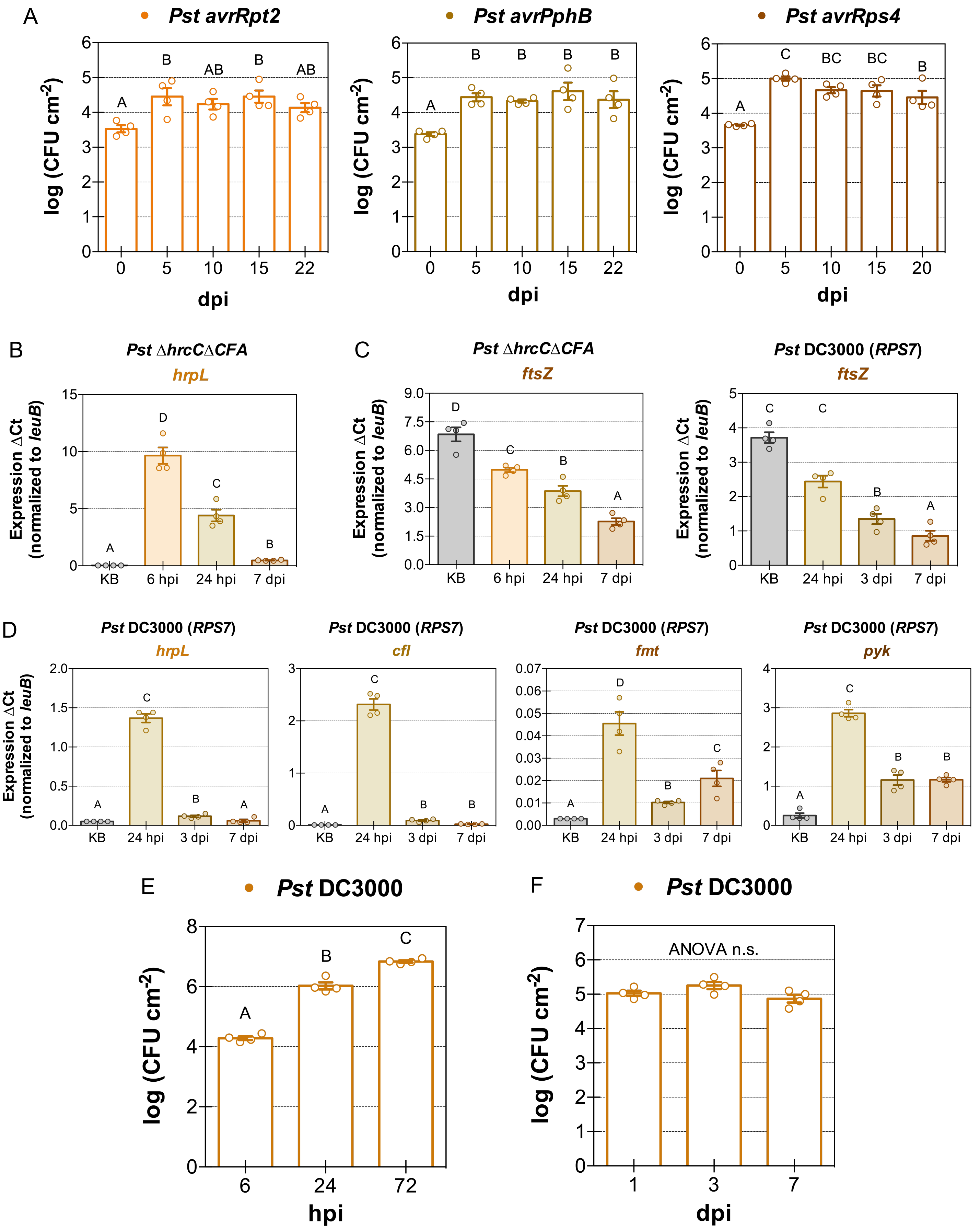
A
Pst avrRpt2
Pst avrPphB
Pst avrRps4
log (CFU cm^{-2})
dpi
A B AB B AB
A B B B B
A C BC BC B
0 5 10 15 22
0 5 10 15 20
B
Pst ΔhrcCΔCFA
hrpL
Expression ΔCt (normalized to leuB)
KB 6 hpi 24 hpi 7 dpi
A D C B
C
Pst ΔhrcCΔCFA
ftsZ
D C B A
Pst DC3000 (RPS7)
ftsZ
KB 24 hpi 3 dpi 7 dpi
C C B A
D
Pst DC3000 (RPS7)
hrpL
A C B A
cfl
A C B B
fmt
A D B C
pyk
A C B B
E
Pst DC3000
hpi
6 24 72
A B C
F
Pst DC3000
ANOVA n.s.
1 3 7

Fig. S7. Similar *in planta* gene expression patterns for non-pathogenic and effector triggered immunity-inducing bacteria. (*A*) Bacterial population density of three effector-triggered immunity-inducing strains (inoculum: 5×10^5 – 2×10^6 CFU mL^{-1}) over the course of 3 weeks after infection of Col-0 plants. In Col-0 plants, effector AvrRpt2 is recognized by RPS2, AvrPphB is recognized by RPS5, whereas AvrRps4 is recognized by RPS4. (*B*) *In planta* gene expression of the RNA polymerase sigma factor controlling virulence in *Pseudomonas syringae tomato* (*Pst*) DC3000 (*hrpL*) after inoculation of *Pst* Δ*hrcC*Δ*CFA* into Col-0 plants. (*C*) An experimental repeat of *ftsZ* expression. Related to Fig. 6*B* and 6*C*. *Pst* Δ*hrcC*Δ*CFA* was inoculated into Col-0 plants, whereas *Pst* DC3000 was inoculated into Bu-22 plants. (*D*) *In planta* gene expression of the virulence master regulator *hrpL*, a gene involved in the biosynthesis of the phytotoxin coronatine (coronafacate ligase, *cfl*), a gene involved in mRNA translation ($tRNA^{fMet}$-formyl transferase, *fmt*), and a gene involved in the final step of the Entner-Doudoroff pathway (pyruvate kinase, *pyk*) after inoculation of *Pst* DC3000 into Bu-22 plants. (*E*) Bacterial population density after high inoculum infiltration of *Pst* DC3000 (inoculum: 10^7 CFU mL^{-1}) into Arabidopsis Bu-22 plants. Note the loss of containment of bacterial growth at high inoculum. (*F*) Bacterial population density after infiltration of *Pst* DC3000 at a low inoculum (4×10^5 CFU mL^{-1}) into Arabidopsis Bu-22 plants. The same plants were also used to collect samples for qRT-PCR.

Individual biological repetitions for each treatment are shown as circles. Error bars indicate the standard error of the mean. When appropriate, different letters indicate differences in means, as judged by a Tukey HSD test ($p <$ 0.05, which was performed between the ΔCt values for qRT-PCR). For qRT-PCR, gene expression was evaluated using *leuB* as the reference gene and the ΔCt method. hpi indicates hours post-inoculation. dpi indicates days post-inoculation.

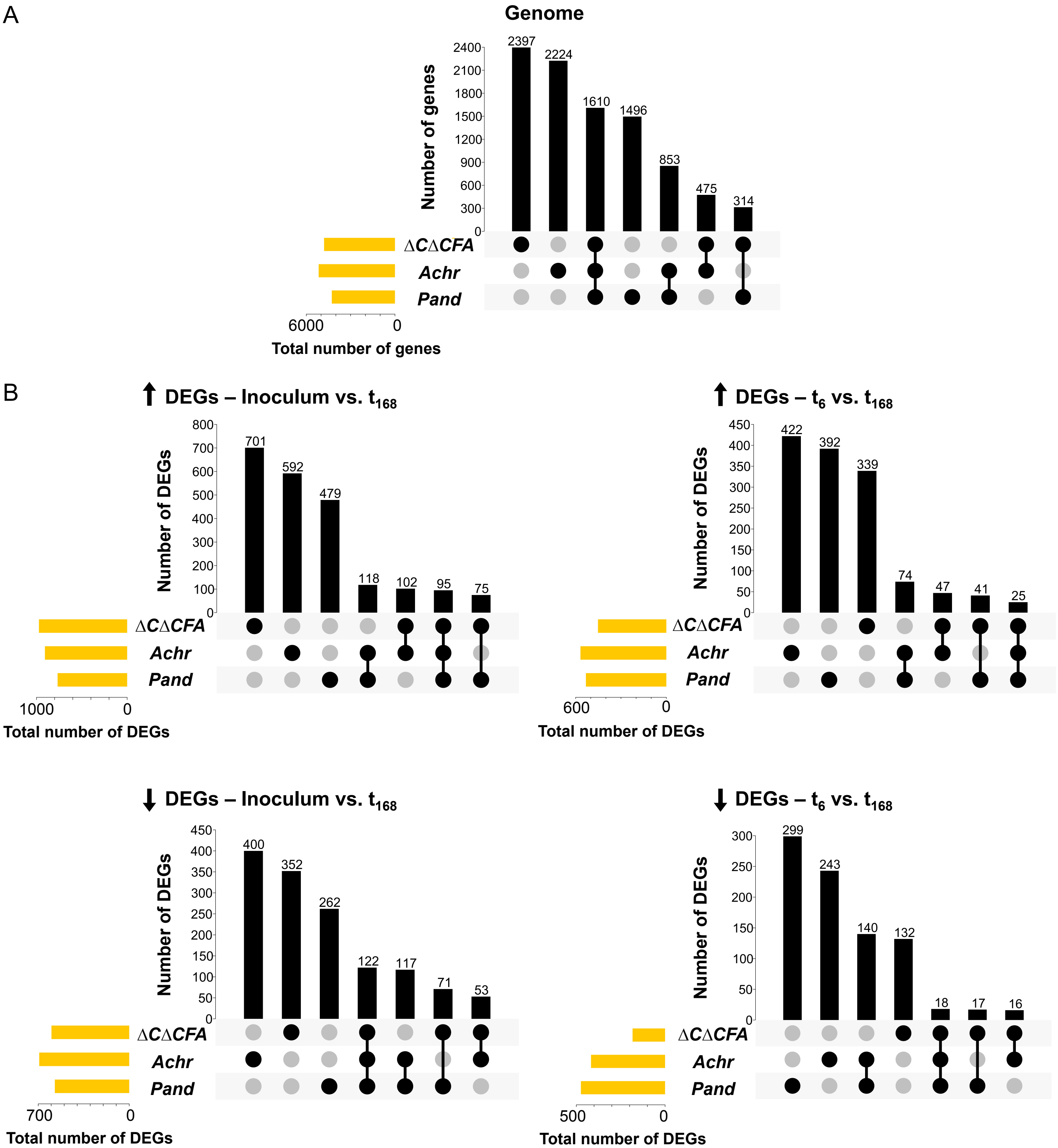

Fig. S8. Number of shared and unique differentially expressed genes between three phyllosphere-inhabiting endophytes. (*A*) Upset graph showing the number of unique and shared orthologous gene groups in the genomes of *Pseudomonas syringae* pv. *tomato* (*Pst*) Δ*hrcC*Δ*CFA* (Δ*C*Δ*CFA*), *Achromobacter xylosoxidans* Col-0-50 (*Achr*), and *Pandoraea* sp. Col-0-28 (*Pand*). The core genome encompasses 1,610 gene groups shared by all three bacterial strains. (*B*) Upset graphs showing the number of unique and shared orthologous up-regulated and down-regulated differentially expressed gene (DEG) groups in *Pst* Δ*hrcC*Δ*CFA*, *A. xylosoxidans* Col-0-50, and *Pandoraea* sp. Col-0-28 in the inoculum or 6 hours post-inoculation (hpi; t_6) of Col-0 plants, when compared to 168 hpi (t_{168}).

Table S1. Enriched up- and down-regulated biological processes of differentially expressed genes (DEG) in *Pseudomonas syringae* pv. *tomato* (*Pst*) ∆*hrcC*∆*CFA*.

**Up-regulated processes in *Pst* ∆*hrcC*∆*CFA***															
**t_6 vs. t_{24}**	***q*-value**	**t_6 vs. t_{168}**	***q*-value**	**t_6 vs. KB**	***q*-value**	**t_6 vs. Stat**	***q*-value**	**t_{24} vs. t_{168}**	***q*-value**	**t_{24} vs. KB**	***q*-value**	**t_{24} vs. Stat**	***q*-value**	**t_{168} vs. KB**	***q*-value**
Virulence	2.12E-14	Virulence	7.43E-25	Virulence	1.30E-19	Virulence	1.05E-28	Virulence	5.36E-18	Virulence	3.86E-20	Virulence	5.92E-26	Virulence	1.31E-11
Deoxyribonucleotide metabolism*	4.33E-02	Transcriptional regulation	5.39E-03	Primary metabolism	6.96E-18	Protein translation	2.43E-09	Regulation of transcription	3.16E-04	Primary metabolism	8.11E-17	Protein translation	6.97E-06	Primary metabolism	5.09E-23
		Transport across membranes and ATP	1.56E-02	Nucleotide metabolism	1.01E-03	Valine biosynthesis*	1.24E-02	Transport across membranes	3.75E-02	Nucleotide metabolism	2.01E-05	Valine biosynthesis*	1.07E-02	Nucleotide metabolism	3.44E-04
		Response to chemical stimulus	2.44E-02	Protein translation	1.41E-30	Carbohydrate metabolism	1.09E-03			Transcription	4.10E-03	Carbohydrate metabolism	2.53E-03	Transcription	2.10E-02
				Ribosome biogenesis	9.98E-05	Alginate biosynthesis	7.61E-07			Protein translation	2.10E-32	Alginate biosynthesis	6.10E-07	Protein translation	9.52E-41
				tRNA aminoacylation	1.57E-06	Transport across membranes	3.54E-04			Ribosome biogenesis	3.07E-05	Transport across membranes	7.21E-04	Ribosome biogenesis	7.88E-05
				Amino acid biosynthesis	1.89E-07	Sulfate transport*	2.91E-02			tRNA aminoacylation	2.77E-06	Phosphate transport	1.34E-02	tRNA aminoacylation	1.07E-07
				Carbohydrate metabolism	3.75E-08	Bacterioferritin*	1.64E-02			Amino acid biosynthesis	5.92E-06	Response to chemical stimulus	4.64E-02	Amino acid metabolism	2.51E-06
				Entner-Doudoroff and Pentose phosphate pathways	2.26E-02	Response to chemical stimulus	7.54E-03			Carbohydrate metabolism	1.35E-06	Response to oxidative stress	1.64E-03	Carbohydrate metabolism	6.13E-07
				Tricarboxylic acid cycle	4.37E-02	Response to oxidative stress	1.63E-02			Entner-Doudoroff and Pentose phosphate pathways	8.25E-04	Bacterioferritin*	1.50E-02	Entner-Doudoroff and Pentose phosphate pathways	1.85E-02
				ATP biosynthesis	7.76E-03					Acetyl-CoA metabolism and Tricarboxylic acid cycle	1.11E-02	3,4-dihydroxybenzoate metabolism*	4.66E-02	Tricarboxylic acid cycle	6.99E-03
				Lipid and LPS biosynthesis	4.48E-02					ATP biosynthesis	4.10E-03			Alginate biosynthesis	2.25E-08
				Peptidoglycan biosynthesis	3.95E-03					Alginate biosynthesis	1.12E-09			Transport across membranes and ATP	4.23E-03
				Alginate biosynthesis	1.55E-07					Transport across membranes	2.75E-02			Response to oxidative stress	1.53E-03
				Transport across membranes	4.54E-03					Response to oxidative stress	1.90E-03				
				Sulfur metabolism	3.11E-02										
				Response to oxidative stress	3.86E-03										

**Up-regulated processes in *Pst* ∆*hrcC*∆*CFA***													
**t_{168} vs. Stat**	***q*-value**	**KB vs. Stat**	***q*-value**	**Log vs. t_6**	***q*-value**	**Log vs. t_{24}**	***q*-value**	**Log vs. t_{168}**	***q*-value**	**Log vs. KB**	***q*-value**	**Log vs. Stat**	***q*-value**
Virulence	1.31E-21	Locomotion and flagellum	9.24E-17	Primary metabolism	3.40E-23	Primary metabolism	3.77E-22	Primary metabolism	1.08E-07	Primary metabolism	4.28E-29	Primary metabolism	1.69E-17
Protein translation	1.61E-11			Nucleotide metabolism	1.09E-11	Nucleotide metabolism	2.25E-08	Nucleotide metabolism	7.69E-05	Nucleotide metabolism	2.89E-10	Nucleotide metabolism	9.11E-06
Valine biosynthesis	6.63E-03			DNA replication	1.97E-04	DNA replication	1.52E-05	DNA replication	8.99E-03	DNA replication	2.54E-02	Regulation of cell shape*	2.71E-02
Carbohydrate metabolism	9.77E-03			DNA repair	9.43E-03	DNA repair	4.13E-02	Response to DNA damage	3.89E-02	DNA repair	6.07E-04	Protein translation	3.91E-23
Alginate biosynthesis	1.78E-07			Regulation of cell shape*	2.28E-02	Protein translation	5.41E-19	Protein translation	7.22E-10	Cytokinesis	3.90E-03	Ribosome biogenesis	1.98E-06
Transport across membranes	1.29E-07			Protein translation	2.87E-16	Ribosome biogenesis	1.24E-05	Ribosome biogenesis	2.04E-03	Regulation of cell shape*	4.42E-02	tRNA aminoacylation	3.23E-05
Sulfate transport	5.13E-05			Ribosome biogenesis	3.66E-06	tRNA aminoacylation	1.57E-11	tRNA aminoacylation	1.23E-07	Transcription	3.23E-02	Amino acid biosynthesis	5.06E-14
Bacterioferritin*	1.05E-02			tRNA aminoacylation	1.15E-08	Amino acid biosynthesis	4.56E-06	Proteolysis	4.79E-02	Protein translation	1.34E-27	Carbohydrate metabolism	2.14E-02
3,4-dihydroxybenzoate metabolism*	3.32E-02			Protein folding*	2.28E-02	Carbohydrate metabolism	4.56E-05	Amino acid biosynthesis	2.20E-04	Ribosome biogenesis	5.29E-08	Entner-Doudoroff and Pentose phosphate pathways	4.18E-02
				Amino acid biosynthesis	1.08E-08	Entner-Doudoroff and Pentose phosphate pathways	1.83E-02	Carbohydrate metabolism	2.16E-02	tRNA aminoacylation	9.46E-09	Tricarboxylic acid cycle	3.23E-05
				Carbohydrate metabolism	9.90E-05	Tricarboxylic acid cycle	7.71E-04	Entner-Doudoroff and Pentose phosphate pathways	3.37E-02	Proteolysis	1.50E-02	ATP biosynthesis	1.72E-03
				Entner-Doudoroff and Pentose phosphate pathways	6.66E-03	ATP biosynthesis	8.32E-04	Tricarboxylic acid cycle	2.83E-03	Amino acid biosynthesis	1.07E-16	Lipid and LPS biosynthesis	1.31E-08
				Tricarboxylic acid cycle	5.21E-03	Lipid and LPS biosynthesis	6.50E-05	ATP biosynthesis	2.44E-03	Carbohydrate metabolism	8.69E-08	Peptidoglycan biosynthesis	6.95E-05
				ATP biosynthesis	1.24E-03	Peptidoglycan biosynthesis	8.25E-05	Carbohydrate transport	8.58E-03	Entner-Doudoroff and Pentose phosphate pathways	3.31E-04	Sulfur metabolism	9.55E-04
				Fermentation	1.68E-02	Vitamin biosynthesis	1.11E-02	Peptidoglycan biosynthesis	1.35E-04	Tricarboxylic acid cycle	8.12E-03	Chorismate metabolism	4.37E-03
				Lipid biosynthesis	2.84E-03	Riboflavin biosynthesis	2.46E-02	Rhamnose biosynthesis	4.52E-02	ATP biosynthesis	4.87E-03	Vitamin biosynthesis	6.89E-05
				Peptidoglycan biosynthesis	2.20E-04			Lipid biosynthesis	1.21E-03	One-carbon metabolism	1.50E-02		
				Rhamnose biosynthesis	2.58E-02			Vitamin biosynthesis	1.87E-03	Peptidoglycan biosynthesis	6.54E-06		
				Vitamin biosynthesis	5.21E-03					Rhamnose biosynthesis	6.46E-03		
				Cobalamin biosynthesis	2.58E-02					Lipid and LPS biosynthesis	1.88E-04		
				Siderophore biosynthesis	2.58E-02					Vitamin biosynthesis	7.63E-07		

Table S1 (Continued).

Down-regulated processes in *Pst* Δ*hrcC*Δ*CFA*															
t_6 vs. t_{24}	*q*-value	t_6 vs. t_{168}	*q*-value	t_6 vs. KB	*q*-value	t_6 vs. Stat	*q*-value	t_{24} vs. t_{168}	*q*-value	t_{24} vs. KB	*q*-value	t_{24} vs. Stat	*q*-value	t_{168} vs. KB	*q*-value
–		–		Locomotion and flagellum	5.30E-19	–		–		Locomotion and flagellum<br>Fatty acid biosynthesis	3.87E-23<br>4.97E-02	Locomotion and flagellum<br>LPS biosynthesis*<br>Siderophore biosynthesis	3.95E-02<br>4.81E-02<br>7.47E-03	Locomotion and flagellum	1.20E-16

Down-regulated processes in *Pst* Δ*hrcC*Δ*CFA*													
t_{168} vs. Stat	*q*-value	KB vs. Stat	*q*-value	Log vs. t_6	*q*-value	Log vs. t_{24}	*q*-value	Log vs. t_{168}	*q*-value	Log vs. KB	*q*-value	Log vs. Stat	*q*-value
Peptidoglycan biosynthesis<br>Siderophore biosynthesis	4.23E-02<br>2.06E-02	Primary metabolism<br>Nucleotide metabolism<br>DNA repair<br>Protein translation<br>Ribosome biogenesis<br>tRNA aminoacylation<br>Protein secretion<br>Amino acid biosynthesis<br>Carbohydrate metabolism<br>ATP biosynthesis<br>One-carbon metabolism<br>Peptidoglycan biosynthesis<br>Alginate biosynthesis<br>Vitamin biosynthesis<br>Siderophore biosynthesis	8.83E-16<br>6.53E-06<br>1.43E-02<br>5.48E-16<br>1.31E-06<br>1.96E-05<br>2.50E-03<br>4.55E-09<br>2.91E-07<br>6.55E-04<br>1.83E-02<br>1.27E-06<br>8.12E-05<br>8.76E-03<br>2.31E-02	Virulence<br>Locomotion and flagellum<br>Two-component system<br>Alginate biosynthesis<br>Response to oxidative stress<br>Bacterioferritin*	2.31E-27<br>8.51E-08<br>7.94E-03<br>3.37E-07<br>5.53E-03<br>2.99E-02	Virulence<br>Locomotion and flagellum<br>Two-component system<br>Branched-chain amino acid catabolism*<br>Carbohydrate metabolism<br>Alginate biosynthesis<br>Response to oxidative stress<br>Bacterioferritin*	2.14E-24<br>1.34E-08<br>1.34E-05<br>2.91E-02<br>1.11E-02<br>3.47E-07<br>5.50E-03<br>2.91E-02	Virulence<br>Locomotion and flagellum<br>Two-component system<br>Branched-chain amino acid catabolism*<br>Carbohydrate metabolism<br>Alginate biosynthesis<br>Sulfate transport*<br>Response to oxidative stress	6.58E-18<br>3.97E-07<br>1.18E-03<br>2.71E-02<br>4.05E-02<br>2.41E-07<br>4.42E-02<br>2.77E-02	Virulence<br>Locomotion and flagellum<br>Two-component system	2.76E-02<br>3.61E-19<br>1.47E-03	Virulence<br>Locomotion and flagellum<br>Two-component system	2.28E-02<br>1.47E-13<br>1.55E-02

Comparisons for enrichment of biological processes were done between the inoculum (KB), 6 (t_6), 24 (t_{24}) and 168 (t_{168}) hours post-inoculation into Arabidopsis leaves, and *in vitro* cultures grown to logarithmic (Log) or stationary (Stat) phase. An asterisk denotes that a biological process had less than 5 DEGs. False discovery rate adjusted *p*-values (*q*-values) calculated by BINGO for each comparison are shown.

Table S2. Enriched up- and down-regulated biological processes of differentially expressed genes (DEG) in *Achromobacter xylosoxidans* Col-0-50.

**Up-regulated processes in *Achromobacter xylosoxidans* Col-0-50**											
**t_6 vs. t_{24}**	***q*-value**	**t_6 vs. t_{168}**	***q*-value**	**t_6 vs. LM**	***q*-value**	**t_{24} vs. t_{168}**	***q*-value**	**t_{24} vs. LM**	***q*-value**	**t_{168} vs. LM**	***q*-value**
–		–		Primary metabolism	6.36E-08	Osmolyte (ectoine) biosynthesis*	3.30E-02	Primary metabolism	4.85E-11	Primary metabolism	2.56E-08
				Protein translation	7.21E-18			RNA primer synthesis for DNA replication*	4.36E-02	Protein translation	5.63E-28
								Protein translation	5.54E-22	Protein folding	1.85E-02
								Carbohydrate metabolism	3.01E-02	Ion transport	2.63E-03
								ATP biosynthesis	1.47E-03	ATP biosynthesis	3.58E-05
								Histidine biosynthesis	7.89E-03	Osmolyte (ectoine) biosynthesis*	3.55E-02
								Osmolyte (ectoine) biosynthesis*	4.36E-02		

**Down-regulated processes in *Achromobacter xylosoxidans* Col-0-50**											
**t_6 vs. t_{24}**	***q*-value**	**t_6 vs. t_{168}**	***q*-value**	**t_6 vs. LM**	***q*-value**	**t_{24} vs. t_{168}**	***q*-value**	**t_{24} vs. LM**	***q*-value**	**t_{169} vs. LM**	***q*-value**
Protein folding	1.73E-04	Primary metabolism	3.37E-02	Amino acid metabolism	3.90E-02	Protein translation	6.37E-04	Locomotion and flagellum	4.71E-02	–	
Ion transport	4.89E-04	Ion transport	1.10E-06	Glutathione metabolism	4.45E-02	Protein folding	8.68E-04				
Sulfate transport	8.20E-04	ATP biosynthesis	1.34E-03			ATP biosynthesis*	1.31E-02				
		Protein translation	1.21E-12								
		Protein folding	3.30E-04								
		Serine family amino acid catabolism*	1.05E-02								
		Sulfate transport	1.28E-02								

Comparisons for enrichment of biological processes were done between the inoculum (LM), 6 (t_6), 24 (t_{24}) and 168 (t_{168}) hours post-inoculation into Arabidopsis leaves. An asterisk denotes that a biological process had less than 5 DEGs. False discovery rate adjusted *p*-values (*q*-values) calculated by BINGO for each comparison are shown.

Table S3. Enriched up- and down-regulated biological processes of differentially expressed genes (DEG) in *Pandoraea* sp. Col-0-28.

Up-regulated processes in *Pandoraea* sp. Col-0-28											
t_6 vs. t_{24}	*q*-value	t_6 vs. t_{168}	*q*-value	t_6 vs. LM	*q*-value	t_{24} vs. t_{168}	*q*-value	t_{24} vs. LM	*q*-value	t_{168} vs. LM	*q*-value
Cell division	9.29E-04	Two-component system	4.04E-03	–		Two-component system	4.22E-02	Nucleotide metabolism	9.57E-03	Primary metabolism	1.93E-06
Peptidoglycan biosynthesis*	2.76E-02							Tricarboxylic acid cycle	4.21E-02	Nucleotide metabolism	1.32E-02
Sulfate transport*	9.29E-04							Aerobic respiration	5.71E-03	Transcription termination*	3.97E-02
								Sulfur compound biosynthesis	4.66E-02	Protein translation	1.73E-24
										tRNA aminoacylation	9.33E-03
										Protein folding	1.32E-02
										Amino acid biosynthesis	4.75E-02
										Urea metabolism*	3.97E-02
										Tricarboxylic acid cycle	6.78E-05
										Aerobic respiration	1.51E-05
										ATP biosynthesis	2.05E-02
										Sulfur compound biosynthesis	1.04E-02
										Response to oxidative stress	4.41E-04
										Siderophore transport*	3.04E-02

Down-regulated processes in *Pandoraea* sp. Col-0-28											
t_6 vs. t_{24}	*q*-value	t_6 vs. t_{168}	*q*-value	t_6 vs. LM	*q*-value	t_{24} vs. t_{168}	*q*-value	t_{24} vs. LM	*q*-value	t_{168} vs. LM	*q*-value
Protein translation	3.39E-03	Primary metabolism	1.86E-15	Primary metabolism	2.76E-04	Primary metabolism	4.80E-09	Allantoin metabolism	2.17E-02	–	
Tricarboxylic acid cycle*	3.78E-02	Nucleotide biosynthesis	2.52E-02	Protein translation	3.87E-02	Protein translation	4.54E-26				
Trehalose metabolism*	5.03E-03	Transcription	2.52E-02	Branched-chain amino acid biosynthesis	3.56E-02	Tricarboxylic acid cycle	2.90E-02				
Nitrate transport	3.78E-02	Protein translation	1.76E-37			ATP biosynthesis	1.50E-02				
		Ribosome biogenesis	3.49E-02	Allantoin metabolism	5.29E-03	Siderophore transport*	4.76E-03				
		Methionine biosynthesis*	4.04E-02	Lipid metabolism	4.29E-02						
		Tricarboxylic acid cycle	2.42E-03								
		ATP biosynthesis	8.04E-03								
		Siderophore transport*	1.87E-02								

Comparisons for enrichment of biological processes were done between the inoculum (LM), 6 (t_6), 24 (t_{24}) and 168 (t_{168}) hours post-inoculation into Arabidopsis leaves. An asterisk denotes that a biological process had less than 5 DEGs. False discovery rate adjusted *p*-values (*q*-values) calculated by BINGO for each comparison are shown.

Table S4. Enriched biological processes based on significantly different expression profiles after STEM (Short-Time series Expression Miner) analysis in *Pseudomonas syringae* pv. *tomato* (*Pst*) Δ*hrcC*Δ*CFA*.

**Enriched *Pst* Δ*hrcC*Δ*CFA* biological processes in Profile:**													
**1**	***q*-value**	**11**	***q*-value**	**26**	***q*-value**	**39**	***q*-value**	**44**	***q*-value**	**45**	***q*-value**	**47**	***q*-value**
Flagellum and locomotion	1.03E-16	Flagellum and locomotion	1.24E-03	Transcriptional regulation	1.68E-02			Virulence	1.25E-03	Primary metabolism	4.62E-03	Virulence	1.39E-10
		Nucleoside transport	2.64E-02					Glutamine family amino acid metabolism	3.47E-02	Purine ribonucleotide biosynthesis	2.04E-02	Primary metabolism	7.88E-13
								Lysine metabolism*	1.71E-02	Protein translation	2.04E-02	Nucleotide metabolism	4.06E-02
								Carbohydrate transport*	1.31E-02			Transcription	2.24E-03
								Vitamin biosynthesis	4.57E-02			Protein translation	8.90E-21
												Ribosome biogenesis	2.14E-06
												tRNA aminoacylation	5.73E-03
												Amino acid biosynthesis	1.00E-03
												Carbohydrate metabolism	4.31E-04
												Entner-Doudoroff and Pentose phosphate	1.99E-02
												Tricarboxylic acid cycle	3.12E-02
												Alginate biosynthesis	5.22E-05
												Transport across membranes	1.36E-03
												Response to oxidative stress	8.31E-03

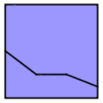
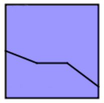
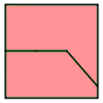
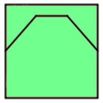
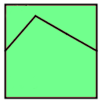
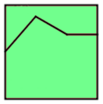
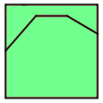

An image of the expression profile is shown below the list of processes, starting from the zero time point (the inoculum; the x-axis denotes time [not to scale], while the y-axis indicates gene expression). The time points used for the analysis were the inoculum, 6, 24, and 168 hours post-inoculation into Arabidopsis leaves. An asterisk denotes that a biological process had less than 5 genes. False discovery rate adjusted *p*-values (*q*-values) for biological process enrichment for each expression profile as calculated by BINGO are shown.

Table S5. Enriched biological processes based on significantly different expression profiles after STEM (Short-Time series Expression Miner) analysis in *Achromobacter xylosoxidans* Col-0-50.

Enriched *Achromobacter xylosoxidans* Col-0-50 biological processes in Profile:									
**9**	***q*-value**	**26**	***q*-value**	**34**	***q*-value**	**39**	***q*-value**	**47**	***q*-value**
Flagellum and locomotion	2.82E-02	Transcriptional regulation	1.94E-04	Primary metabolism	2.50E-02	Primary metabolism	7.70E-04	Primary metabolism	6.18E-06
				Nucleotide metabolism	4.64E-03	Histidine biosynthesis	7.26E-03	Protein translation	1.69E-17
				Vitamin biosynthesis	2.00E-02	Lipid biosynthesis	1.89E-03	ATP biosynthesis	4.11E-03
								Osmolyte (Ectoine) biosynthesis*	6.28E-03

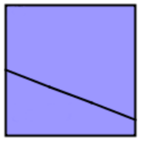
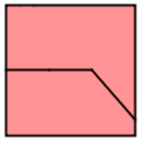
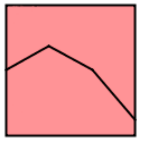
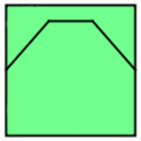
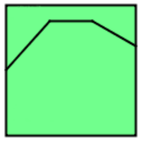

An image of the expression profile is shown below the list of processes, starting from the zero time point (the inoculum; the x-axis denotes time [not to scale], while the y-axis indicates gene expression). The time points used for the analysis were the inoculum, 6, 24, and 168 hours post-inoculation into Arabidopsis leaves. An asterisk denotes that a biological process had less than 5 genes. False discovery rate adjusted *p*-values (*q*-values) for biological process enrichment for each expression profile as calculated by BINGO are shown.

Table S6. Enriched biological processes based on significantly different expression profiles after STEM (Short-Time series Expression Miner) analysis in *Pandoraea* sp. Col-0-28.

**Enriched *Pandoraea* sp. Col-0-28 biological processes in Profile:**							
**39**	***q*-value**	**44**	***q*-value**	**47**	***q*-value**	**49**	***q*-value**
Transcriptional regulation	3.10E-02	Two-component system	1.45E-02	Primary metabolism	5.02E-10	Primary metabolism	1.12E-03
		Transposition	4.82E-03	Nucleotide metabolism	4.08E-02	Protein translation	5.92E-15
				tRNA aminoacylation	2.32E-02	Tricarboxylic acid cycle	1.32E-02
				RNA processing	7.04E-03	ATP biosynthesis	6.10E-03
				Amino acid biosynthesis	1.31E-03	Inositol phosphate dephosphorylation*	3.74E-02
				Vitamin biosynthesis	1.61E-02		
				Ubiquinone biosynthesis	1.86E-02		

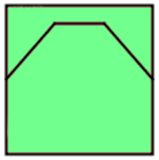
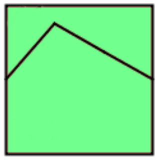
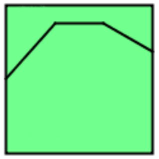
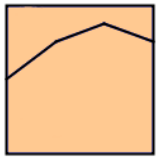

An image of the expression profile is shown below the list of processes, starting from the zero time point (the inoculum; the x-axis denotes time [not to scale], while the y-axis indicates gene expression). The time points used for the analysis were the inoculum, 6, 24, and 168 hours post-inoculation into Arabidopsis leaves. An asterisk denotes that a biological process had less than 5 genes. False discovery rate adjusted *p*-values (*q*-values) for biological process enrichment for each expression profile as calculated by BINGO are shown.

Table S7. Secondary metabolite biosynthetic gene clusters in *Pseudomonas syringae* pv. *tomato ΔhrcCΔCFA* whose expression is differentially regulated *in planta*.

Locus name	Gene name	$log_2(t_6/KB)$	< 0.05	$log_2(t_{168}/KB)$	< 0.05	$log_2(t_6/t_{168})$	< 0.05	Annotation description
**N-acetylglutaminylglutamine amide**								
PSPTO_1627	*PSPTO_1627*	-1.01		-1.53	yes	0.52		Short chain dehydrogenase
PSPTO_1631	*PSPTO_1631*	3.26	yes	3.60	yes	-0.34		
PSPTO_1632	*PSPTO_1632*	3.21	yes	2.35	yes	0.86	yes	GNAT family acetyltransferase
PSPTO_1633	*asnB*	2.50	yes	2.33	yes	0.17		Asparagine synthetase
**Coronatine**								
PSPTO_4671		-0.60	yes	-0.97	yes	0.37		Alkaline D-peptidase
PSPTO_4680	*cfl*							Coronafacic acid synthetase, ligase component; deleted in *Pst ΔhrcCΔCFA*.
PSPTO_4681	*cfa1*							Coronafacic acid synthetase, acyl carrier protein component; deleted in *Pst ΔhrcCΔCFA*.
PSPTO_4682	*cfa2*							Coronafacic acid synthetase, dehydratase component; deleted in *Pst ΔhrcCΔCFA*.
PSPTO_4683	*cfa3*							Coronafacic acid beta-ketoacyl synthetase component; deleted in *Pst ΔhrcCΔCFA*.
PSPTO_4684	*cfa4*							Coronafacic acid synthetase component; deleted in *Pst ΔhrcCΔCFA*.
PSPTO_4685	*cfa5*							Coronafacic acid synthetase, ligase component; deleted in *Pst ΔhrcCΔCFA*.
PSPTO_4686	*cfa6*							Coronafacic acid polyketide synthase I; deleted in *Pst ΔhrcCΔCFA*.
PSPTO_4687	*cfa7*							Coronafacic acid polyketide synthetase II; deleted in *Pst ΔhrcCΔCFA*.
PSPTO_4689	*cfa8*							Crotonyl-CoA reductase; deleted in *Pst ΔhrcCΔCFA*.
PSPTO_4690	*cfa9*							CFA synthetase, thioesterase component; deleted in *Pst ΔhrcCΔCFA*.
PSPTO_4699		-0.85	yes	-1.05	yes	0.20		Non-ribosomal peptide synthetase, terminal component
PSPTO_4704	*corR*	0.69		-0.59		1.28		DNA-binding response regulator CorR
PSPTO_4705	*corS*	0.00		-0.05	yes	0.04		Sensor histidine kinase CorS
PSPTO_4706	*corP*	-1.18		-0.59	yes	-0.59		Response regulator CorP
PSPTO_4707	*cmaD*	3.25	yes	-0.45		3.70	yes	Coronamic acid synthetase CmaD
PSPTO_4708	*cmaE*	1.92	yes	-0.31		2.23	yes	Coronamic acid synthetase CmaE
PSPTO_4709	*cmaA*	3.21	yes	0.56		2.66	yes	Coronamic acid synthetase CmaA
PSPTO_4710	*cmaB*	3.52	yes	1.13	yes	2.39	yes	Coronamic acid synthetase CmaB
PSPTO_4711	*cmaC*	3.19	yes	-2.36	yes	5.56	yes	Coronamic acid synthetase CmaC
PSPTO_4712	*cmaT*	2.63	yes	0.98	yes	1.65	yes	Coronamic acid synthetase, thioesterase component
PSPTO_4714	*cmaU*	0.05		-2.20	yes	2.25	yes	CmaU protein
PSPTO_4723	*cmaL*	4.11	yes	1.12	yes	3.00	yes	Coronofacate ligase CmaL

Table S7 (Continued).

Locus name	Gene name	$log_2(t_6/KB)$	< 0.05	$log_2(t_{168}/KB)$	< 0.05	$log_2(t_6/t_{168})$	< 0.05	Annotation description
				**Pyoverdin**				
PSPTO_2120	*PSPTO_2120*	-1.09	yes	-2.69	yes	1.61	yes	Peptidase, M20/M25/M40 family
PSPTO_2123	*PSPTO_2123*	-0.88	yes	0.70		-1.58	yes	Sensor histidine kinase
PSPTO_2128	*PSPTO_2128*	2.63	yes	1.11	yes	1.52	yes	Response regulator
PSPTO_2130	*PSPTO_2130*	2.88	yes	2.28	yes	0.60		LuxR family DNA-binding response regulator
PSPTO_2131	*PSPTO_2131*	2.11	yes	0.59	yes	1.51	yes	Sensor histidine kinase
PSPTO_2133	*sigX*	2.37	yes	1.64	yes	0.73	yes	RNA polymerase sigma-70 family protein
PSPTO_2134	*pvdG*	-3.71	yes	-2.47		0.00		Pyoverdine synthetase, thioesterase component
PSPTO_2135	*pvsA, pvdL*	-2.24	yes	-1.01	yes	-1.23	yes	Pyoverdine chromophore precursor synthetase
PSPTO_2136	*daT, pvdH*	-0.68		0.30		-0.98		2,4-diaminobutyrate 4-transaminase
PSPTO_2137	*PSPTO_2137*	-0.50		-1.91	yes	1.41		MbtH-like protein
PSPTO_2140	*PSPTO_2140*	-1.50		0.50		-1.99		Cation ABC transporter ATP-binding protein
PSPTO_2141	*PSPTO_2141*	-1.57	yes	-1.74	yes	0.17		Cation ABC transporter substrate-binding protein
PSPTO_2146	*PSPTO_2146*	-0.65		-4.92	yes	4.27	yes	Pyoverdine biosynthesis regulatory gene
PSPTO_2147	*PSPTO_2147*	-0.16	yes	-0.15	yes	-0.02		Pyoverdine sidechain peptide synthetase I, epsilon-Lys module
PSPTO_2148	*PSPTO_2148*	-0.75		-0.17		-0.58		Pyoverdine sidechain peptide synthetase II, D-Asp-L-Thr component
PSPTO_2149	*pvdD*	-0.52		0.49		-1.00		Pyoverdine sidechain peptide synthetase III, L-Thr-L-Ser component
PSPTO_2150	*PSPTO_2150*	-0.81	yes	-0.12		-0.69		Pyoverdine sidechain peptide synthetase IV, D-Asp-L-Ser component
PSPTO_2151	*fpvA*	0.99	yes	-0.65		1.64	yes	TonB-dependent siderophore receptor
PSPTO_2152	*PSPTO_2152*	-0.80		-0.53		-0.27		TonB-dependent siderophore receptor
PSPTO_2153	*pvdE*	-0.22	yes	-0.36	yes	0.14		Pyoverdine ABC transporter, ATP-binding/permease protein
PSPTO_2154	*pvdO*	-0.07	yes	-1.24		1.17	yes	
PSPTO_2155	*pvdN*	-1.92	yes	-0.09	yes	-1.83	yes	Class V aminotransferase
PSPTO_2156	*pvdM*	0.51		-3.80	yes	4.31	yes	Renal dipeptidase family protein
PSPTO_2158	*PSPTO_2158*	0.10		0.26		-0.17		Outer membrane efflux protein
PSPTO_2159	*PSPTO_2159*	-1.18	yes	-1.53	yes	0.35		Macrolide ABC efflux protein
PSPTO_2160	*PSPTO_2160*	-3.15	yes	-0.02	yes	-3.13		RND family efflux transporter MFP subunit
PSPTO_2161	*pvdQ*	-1.07	yes	-0.55	yes	-0.51		Penicillin amidase family protein

Table S7 (Continued).

Locus name	Gene name	$log_2(t_6/KB)$	< 0.05	$log_2(t_{168}/KB)$	< 0.05	$log_2(t_6/t_{168})$	< 0.05	Annotation description
				**Yersiniabactin**				
PSPTO_2583		-1.88		-3.05		1.17		Glutamine amidotransferase, class-II protein
PSPTO_2590		1.59	yes	2.75	yes	-1.16	yes	Luciferase family protein
PSPTO_2591		-0.69	yes	0.34		-1.03		Diguanylate cyclase
PSPTO_2592	*saxG*	0.10		0.45		-0.35		Aliphatic isothiocyanate resistance protein SaxG; AcrB/AcrD/AcrF family
PSPTO_2593		0.77	yes	-3.34	yes	4.11	yes	Multidrug resistance protein, AcrA/AcrE family
PSPTO_2595	*pchA*	-0.42		0.14		-0.56		Isochorismate synthase
PSPTO_2596	*pchB*	-0.62	yes	-0.96	yes	0.34		Isochorismate pyruvate-lyase
PSPTO_2597	*irp5, ybtE*	0.28		-0.15		0.43		Yersiniabactin synthetase, salycilate ligase component
PSPTO_2598	*irp4, ybtT*	-0.57		-0.50		-0.07		Yersiniabactin synthetase, thioesterase component
PSPTO_2599	*irp3, ybtU*	-1.32		-0.71		-0.61		Yersiniabactin synthetase, thiazolinyl reductase component
PSPTO_2600	*irp1, hmwp1*	-0.64	yes	-0.48	yes	-0.15		Yersiniabactin polyketide/non-ribosomal peptide synthetase
PSPTO_2601	*ybtX*	-0.09		-1.52	yes	1.43	yes	Membrane protein
PSPTO_2602	*hmwp2*	-0.57	yes	-1.13	yes	0.55		Yersiniabactin non-ribosomal peptide synthetase
PSPTO_2603	*ybtQ*	-1.09		-1.25		0.16		ABC transporter ATP-binding protein/permease
PSPTO_2604	*ybtP*	-0.84	yes	-1.73	yes	0.89		ABC transporter ATP-binding protein/permease
PSPTO_2605	*fyuA*	-0.99	yes	-0.18		-0.81	yes	TonB-dependent siderophore receptor
PSPTO_2606	*ybtA*	-0.87	yes	-1.37	yes	0.50		AraC family transcriptional regulator
PSPTO_2607		-0.80		0.18		-0.99		TonB-dependent siderophore receptor
PSPTO_2612		-0.97		-1.61		0.64		GNAT family acetyltransferase
PSPTO_2614		1.43	yes	-0.38		1.81	yes	Dioxygenase, TauD/TfdA family
PSPTO_2617		0.84		0.93		-0.09		LysR family transcriptional regulator
PSPTO_2618	*argD-2*	-0.48		-1.90	yes	1.42		Acetylornithine aminotransferase

Gene expression used the logarithm in base 2 fold-change values calculated by DESeq2 after aligning the reads with StringTie when comparing the inoculum (KB) with 6 (t_6) and 168 (t_{168}) hours post-inoculation (hpi) into Arabidopsis leaves, and 6 with 168 hpi. Genes that are up-regulated are highlighted in cyan, while those that are down-regulated are highlighted in magenta. A column indicating if a gene was differentially regulated, as determined by DESeq2 or Cuffdiff (false discovery rate adjusted $p < 0.05$), is also shown. Gene clusters were identified as enriched using a hypergeometric enrichment test. The yersiniabactin gene cluster did not show differential expression, but is included as it is a cluster already known to be associated with secondary metabolite biosynthesis.

Table S8. Secondary metabolite biosynthetic gene clusters in *Achromobacter xylosoxidans* Col-0-50 whose expression is differentially regulated *in planta*.

Locus name	Gene name	$log_2(t_6/LM)$	< 0.05	$log_2(t_{168}/LM)$	< 0.05	$log_2(t_6/t_{168})$	< 0.05	Annotation description
**Siderophore, similar to desferrioxamine**								
FLPKDCKG_00714	*bcr_1*	-4.39	yes	-2.87	yes	-1.52		Bicyclomycin resistance protein
FLPKDCKG_00715	*pchR_3*	-4.22	yes	-2.31	yes	-1.91	yes	Regulatory protein PchR
FLPKDCKG_00718	*iucC*	-5.23	yes	-3.00	yes	-2.23		Aerobactin synthase
FLPKDCKG_00719	*iucB*	-4.04	yes	-3.53	yes	-0.50		N(6)-hydroxylysine O-acetyltransferase
FLPKDCKG_00720	*iucD*	-5.42	yes	-3.15	yes	-2.27		L-lysine N6-monooxygenase
FLPKDCKG_00721	*fetA*	-0.46		-0.85		0.39		Putative iron export ATP-binding protein FetA
FLPKDCKG_00722	*fetB*	-0.92		-5.36	yes	4.43		Putative iron export permease protein FetB.
**Ectoine**								
FLPKDCKG_01732	*ectD_1*	6.13	yes	4.58	yes	1.55	yes	Ectoine dioxygenase
FLPKDCKG_01733	*ectC*	6.68	yes	5.20	yes	1.48	yes	L-ectoine synthase
FLPKDCKG_01734	*ectB*	7.33	yes	5.45	yes	1.88	yes	Diaminobutyrate--2-oxoglutarate transaminase
FLPKDCKG_01735	*ectA*	5.67	yes	3.05	yes	2.62	yes	L-2,4-diaminobutyric acid acetyltransferase
FLPKDCKG_01736	*yusO*	2.59	yes	1.26	yes	1.32	yes	Putative HTH-type transcriptional regulator YusO
**Non-ribosomal peptide synthase-like**								
FLPKDCKG_01927	*livH_6*	-0.83		0.88		-1.71		High-affinity branched-chain amino acid transport system permease protein LivH
FLPKDCKG_01928	*livF_6*	0.80		2.66		-1.86		High-affinity branched-chain amino acid transport ATP-binding protein LivF
FLPKDCKG_01929	*lptB_3*	-1.96		3.24		-5.20		Lipopolysaccharide export system ATP-binding protein LptB
FLPKDCKG_01930	*gno_1*	-0.59	yes	2.50		-3.09	yes	Gluconate 5-dehydrogenase
FLPKDCKG_01932	-	-1.08		2.14		-3.23		
FLPKDCKG_01933	*fabG2*	-0.23		0.27		-0.50		Putative oxidoreductase
FLPKDCKG_01934	*kipR_2*	-0.31		-0.23	yes	-0.08		HTH-type transcriptional regulator KipR
FLPKDCKG_01947	*dhbF*	-0.69		0.13		-0.82	yes	Dimodular nonribosomal peptide synthase
FLPKDCKG_01952	*tctD_1*	-0.54		0.13		-0.67		Transcriptional regulatory protein tctD
FLPKDCKG_01953	*qseC_2*	0.11		1.27	yes	-1.16		Sensor protein QseC
FLPKDCKG_01954	*proP_3*	2.39	yes	2.91	yes	-0.52		Proline/betaine transporter
FLPKDCKG_01957	*dosC_2*	0.24		-1.01	yes	1.25	yes	Diguanylate cyclase DosC
FLPKDCKG_01958	*pyrB_1*	-1.52	yes	-0.63		-0.89	yes	Aspartate carbamoyltransferase catalytic subunit
**Resorcinol**								
FLPKDCKG_03746	*yajR_2*	0.83		-0.56		1.39		Inner membrane transport protein YajR
FLPKDCKG_03748	*bioC_1*	0.44		-0.21		0.66		Malonyl-[acyl-carrier protein] O-methyltransferase
FLPKDCKG_03751	*acuR*	-0.13		-0.37		0.24		Transcriptional regulator AcuR
FLPKDCKG_03754	-	-2.51	yes	-2.94	yes	0.43		
FLPKDCKG_03756	*glcC_2*	0.40		-0.64		1.04		Glc operon transcriptional activator
FLPKDCKG_03766	*fabH2*	0.46		-0.30		0.76		3-oxoacyl-[acyl-carrier-protein] synthase 3 protein 2
FLPKDCKG_03774	*yijE_8*	0.95	yes	-0.09		1.04		Putative cystine transporter YijE
FLPKDCKG_03775	*bmr3_1*	0.82	yes	0.60		0.23		Multidrug resistance protein 3
FLPKDCKG_03777	*rkpK_2*	0.51	yes	0.19		0.32		UDP-glucose 6-dehydrogenase
FLPKDCKG_03778	-	0.68	yes	0.48		0.20		UDP-glucose 4-epimerase
FLPKDCKG_03779	*pseB*	0.45		0.24		0.20		UDP-N-acetylglucosamine 4,6-dehydratase (inverting)
FLPKDCKG_03780	*pseC*	0.92	yes	0.41		0.51		UDP-4-amino-4,6-dideoxy-N-acetyl-beta-L-altrosamine transaminase

Gene expression used the logarithm in base 2 fold-change values calculated by DESeq2 after aligning the reads with StringTie when comparing the inoculum (LM) with 6 (t_6) and 168 (t_{168}) hours post-inoculation (hpi) into Arabidopsis leaves, and 6 with 168 hpi. Genes that are up-regulated are highlighted in cyan, while those that are down-regulated are highlighted in magenta. A column indicating if a gene was differentially regulated, as determined by DESeq2 or Cuffdiff (false discovery rate adjusted $p < 0.05$), is also shown. Gene clusters were identified as enriched using a hypergeometric enrichment test.

Table S9. Enrichment of plant-associated genes that were up-regulated within the differentially expressed genes in the transcriptome analysis.

Strains	Comparison	*q*-value
Pseudomonas syringae pv. *tomato* Δ*hrcC*Δ*CFA*	Up t_6 vs. KB	1.000
	Up t_{24} vs. KB	1.000
	Up t_{168} vs. KB	1.000
	Up Log vs. KB	1.000
	Up Stat vs. KB	1.000
	Up t_6 vs. t_{24}	0.015
	Up t_6 vs. t_{168}	4.84E-05
	Up t_{24} vs. t_{168}	2.67E-05
	Up t_6 vs. Log	4.32E-09
	Up t_6 vs. Stat	0.003
	Up t_{24} vs. Log	2.86E-09
	Up t_{24} vs. Stat	1.99E-05
	Up t_{168} vs. Log	5.37E-07
	Up t_{168} vs. Stat	0.049
	Up Log vs. Stat	1.000
Achromobacter xylosoxidans Col-0-50	Up t_6 vs. LM	0.998
	Up t_{24} vs. LM	0.998
	Up t_{168} vs. LM	0.998
	Up t_6 vs. t_{24}	0.220
	Up t_6 vs. t_{168}	0.658
	Up t_{24} vs. t_{168}	0.228
Pandoraea sp. Col-0-28	Up t_6 vs. LM	0.072
	Up t_{24} vs. LM	0.182
	Up t_{168} vs. LM	0.182
	Up t_6 vs. t_{24}	0.002
	Up t_6 vs. t_{168}	0.002
	Up t_{24} vs. t_{168}	0.002

The Benjamini-Hochberg adjusted *p*-value (*q*-value) for each hypergeometric enrichment test for up-regulated plant-associated genes is shown. Comparisons were performed between the inoculum (KB or LM), 6 (t_6), 24 (t_{24}), and 168 (t_{168}) hours post-inoculation into Col-0 plants, and *in vitro* logarithmic (Log) and stationary (Stat) populations. Adjusted *p*-values that were significant (p < 0.05) are highlighted in cyan.

Table S10. Bacterial strains used in this study.

Strains	Comments	References
Achromobacter xylosoxidans Col-0–50	Non-pathogenic Betaproteobacteria isolated from Arabidopsis Col-0 leaves. Kanamycin-resistant strain.	(32)
Escherichia coli DH5α	*E. coli* strain used for cloning of most constructs.	(33)
E. coli DH5α pUC18-mini-Tn7T-Gm	Strain used for integration of genes into the attTn7 site of *Pseudomonas*.	(5)
E. coli MaH1	Strain is a derivative of *E. coli* DH5α with attTn7::*pir116* integrated into the genome for expression of the π protein, which is necessary for R6K plasmid replication.	(9)
E. coli PIR2 pBG42	Strain used for integration of *msfGFP* into the attTn7 site of *Pseudomonas*.	(6)
E. coli RHO3 pTNS3	Tn7 transposase helper strain, site-specific TnsD pathway only.	(8)
E. coli RHO5	*E. coli* SM10 derivative carrying the *pir116* gene for replication of R6K plasmids. Used for conjugation of plasmids into *Pseudomonas*.	(9)
E. coli RHO5 pUC18-mini-Tn7T-Gm::*tetR(BD)-*P_{tet}*-mCitrine_mCerulean3-BCD2-*P_{14g}	Strain used for integration of a dual fluorescent reporter for cell division into the attTn7 site of *Pseudomonas*. *mCitrine* is under the control of a tetracycline inducible promoter, while *mCerulean3* is under the control of the constitutive promoter P_{14g} and the translational enhancer BCD2.	This study
Pandoraea sp. Col-0–28	Non-pathogenic Betaproteobacteria isolated from Arabidopsis Col-0 leaves. Ampicillin-resistant strain.	(32)
Pseudomonas syringae pv. *tomato* DC3000	Rifampicin-resistant derivative from *Pseudomonas syringae* pv. *tomato* strain DC52.	(34)
Pst DC3000 attTn7::*tetR(BD)-*P_{tet}*-mCitrine_mCerulean-BCD2-*P_{14g}	*Pst* DC3000 strain carrying the cell division reporter: *mCitrine* expressed under the control of a tetracycline inducible promoter and *mCerulean3* under the control of the constitutive promoter P_{14g} and the translational enhancer BCD2.	This study
Pst DC3000 pDSK600::*avrPphB*	*Ps*t DC3000 expressing *avrPphB* effector from plasmid pPPY3031 (pDSK600::*avrPphB*). AvrPphB is expressed under the control of a triple lacUV5 promoter.	(35)
Pst DC3000 pDSK600::*avrRpt2*	*Ps*t DC3000 expressing *avrRpt2* effector from plasmid pDSK600.	(36)
Pst DC3000 pVSP61::*avrRps4*	*Ps*t DC3000 expressing *avrRps4* effector from plasmid pVSP61.	(37)
Pst Δ*hrcC*Δ*CFA*	Non-pathogenic *Pseudomonas syringae* strain with the coronafacic acid cluster (*CFA*; 11 genes are deleted) and a gene necessary for type III secretion system formation (*hrcC*) deleted.	(38)
Pst Δ*hrcC*Δ*CFA* attTn7T::*tetR(BD)-*P_{tet}*-mCitrine_mCerulean-BCD2-*P_{14g}	Non-pathogenic *Pst* Δ*hrcC*Δ*CFA* strain carrying the cell division reporter: *mCitrine* expressed under the control of a tetracycline inducible promoter and *mCerulean3* under the control of the constitutive promoter P_{14g} and the translational enhancer BCD2.	This study
Rhodococcus sp. 964	Non-pathogenic Actinobacteria strain isolated from Arabidopsis leaves. Rifampicin-resistant strain.	(39)

Table S11. Plant genotypes used in this study.

Plant genotypes	Comments	References
A. thaliana Bu-22	Carries *RPS7*, a resistance locus that recognizes the AvrPto effector.	(13)
Arabidopsis thaliana Col-0		
A. thaliana Col-0 *bak1-5 bkk1-1 cerk1*	Three genes involved in pattern recognition receptor signaling are mutated: *bak1*, *bkk1*, and *cerk1*.	(40)

Table S12. Primer sequences used in this study.

Primer name	Primer sequence (5' to 3')	Comments	References
AVL001	**GCCTGCAAGGGAGCTCACTAG**TTAATTAAGCCCATTGACAAGG	Used with primer AVL002 to amplify the 14g promoter and BCD2 translational enhancer from pBG42 for Gibson assembly of pUC18-mini-Tn7T-Gm::*tetR(BD)-P_{tet}-mCitrine_ mCerulean3-BCD2-P_{14g}*. In bold, sequence complementary to pUC18-mini-Tn7T-Gm.	This study
AVL002	**CCTCGCCCTTGCTCACCAT**TAGAAAACCTCCTTAGCATG	Used with primer AVL001 to amplify the 14g promoter and BCD2 translational enhancer from pBG42 for Gibson assembly of pUC18-mini-Tn7T-Gm::*tetR(BD)-P_{tet}-mCitrine_ mCerulean3-BCD2-P_{14g}*. In bold, sequence complementary to *mCerulean3*.	This study
AVL003	**CATGCTAAGGAGGTTTTCTA**ATGGTGAGCAAGGGCGAGG	Used with primer AVL004 to amplify *mCerulean3* for Gibson assembly *of* pUC18-mini-Tn7T-Gm::*tetR(BD)-P_{tet}-mCitrine_ mCerulean3-BCD2-P_{14g}* In bold, sequence complementary to the BCD2 translational enhancer.	This study
AVL004	CCGAGAcCGAAGAGGCACTA	Used with primer AVL003 for Gibson assembly of pUC18-mini-Tn7T-Gm::*tetR(BD)-P_{tet}-mCitrine_ mCerulean3-BCD2-P_{14g}*.	This study
AVL005	GTTTAGCGGACGACAATCCT	Used with primer AVL006 to check for transposants while with primer AVL007, it is used to check for bacteria in which transposition has not occurred. Primer hybridizes near the 5' end of the CDS of *PSPTO_5594*.	Kvitko lab (University of Georgia)
AVL006	ATTAGCTTACGACGCTACACCC	Used with primer AVL005 to check for transposants.	Kvitko lab (University of Georgia)
AVL007	GCAGCTGCTTTCGTATTATGTG	Used with primer AVL008 to check for transposants while with primer AVL005, it is used to check for bacteria in which transposition has not occurred. Primer hybridizes near the 3' end of *glmS* (*PSPTO_5595*).	Kvitko lab (University of Georgia)
AVL008	CACAGCATAACTGGACTGATTTC	Used with primer AVL007 to check for transposants.	Kvitko lab (University of Georgia)
AVL009	TTGACTcatATGGTCGCATTTGCAGGAT	Used with primer AVL010 to amplify *hrpA* (*PSPTO_1381*) from *Pseudomonas syringae* pv. *tomato* (*Pst*) DC3000 to check for genomic DNA contamination in RNA samples. In lowercase, an added NdeI site.	This study
AVL010	GCCgaattcTTAGTAACTGATACCTTTAGCG	Used with primer AVL009 to amplify *hrpA* (*PSPTO_1381*) from *Pst* DC3000 to check for genomic DNA contamination in RNA samples. In lowercase, an added EcoRI site.	This study

Table S12 (Continued).

Primer name	Primer sequence (5' to 3')	Comments	References
AVL011	GAAACTGATCGACTTGCAGG	Used with primer AVL012 to amplify *rpoD* (*FEPFGCPE_00166*) of *Pandoraea* sp. Col-0-28 to check for genomic DNA contamination in RNA samples.	This study
AVL012	ATGTGCACCGGAATACGGAT	Used with primer AVL011 to amplify *rpoD* (*FEPFGCPE_00166*) of *Pandoraea* sp. Col-0-28 to check for genomic DNA contamination in RNA samples.	This study
AVL013	TGCAGTGGGTCGTCGACGAA	Used with primer AVL014 to amplify *rpoD1* (*FLPKDCKG_01693*) of *Achromobacter xylosoxidans* Col-0-50 to check for genomic DNA contamination in RNA samples.	This study
AVL014	CGACGGTACTCGAACTTGTC	Used with primer AVL013 to amplify *rpoD1* (*FLPKDCKG_01693*) of *A. xylosoxidans* Col-0-50 to check for genomic DNA contamination in RNA samples.	This study
AVL015	ATCTCATACTGACCCATATGTTCC	Used with primer AVL016 to amplify microsatellite marker *nga151a* from *Arabidopsis thaliana* to check for genomic DNA contamination in RNA samples.	(41)
AVL016	ATTGTACAGTCTAAAAGCGAGAG	Used with primer AVL015 to amplify microsatellite marker *nga151a* from *A. thaliana* to check for genomic DNA contamination in RNA samples.	(41)
AVL017	CTGACCGGTGGCATCTATTT	Used with primer AVL018 for quantitative real-time PCR (qRT-PCR) of reference *Pst* DC3000 gene *leuB* (*PSPTO_2175*).	This study
AVL018	ATTTCGCTCTCGCTGTACG	Used with primer AVL017 for qRT-PCR of reference *Pst* DC3000 gene *leuB* (*PSPTO_2175*).	This study
AVL019	GTCTCCAGAGCGATTTGTTG	Used with primer AVL020 for qRT-PCR of *Pst* DC3000 effector gene *avrPto* (*PSPTO_4001*).	(42)
AVL020	GGGCTAGGAGAAAGTGTTGA	Used with primer AVL019 for qRT-PCR of *Pst* DC3000 effector gene *avrPto* (*PSPTO_4001*).	(42)
AVL021	TGCTCGTCTCGTCGCCAAG	Used with primer AVL022 for qRT-PCR of *Pst* DC3000 *cfl* (*PSPTO_4680*); a coronafacic acid ligase involved in the biosynthesis of the toxin coronatine.	(38)
AVL022	CGATACCCTTAGTTAGTCCTGTGG	Used with primer AVL021 for qRT-PCR of *Pst* DC3000 *cfl* (*PSPTO_4680*); a coronafacic acid ligase involved in the biosynthesis of the toxin coronatine.	(38)
AVL023	CAACATGACATCCCGGTCA	Used with primer AVL024 for qRT-PCR of *Pst* DC3000 *fmt* (*PSPTO_0178*), a $tRNA^{fMet}$-formyl transferase involved in formylating the methionine of the initiator tRNA.	This study

Table S12 (Continued).

Primer name	Primer sequence (5' to 3')	Comments	References
AVL024	CGGAATATCCAGCACCACTT	Used with primer AVL023 for qRT-PCR of *Pst* DC3000 *fmt* (*PSPTO_0178*), a $tRNA^{fMet}$-formyl transferase involved in formylating the methionine of the initiator tRNA.	This study
AVL025	TCACCATTCCCAACGAGAAG	Used with primer AVL026 for qRT-PCR of *Pst* DC3000 cell division gene *ftsZ* (*PSPTO_4403*).	This study
AVL026	CGCTTGATGATGTCGGAGATA	Used with primer AVL025 for qRT-PCR of *Pst* DC3000 cell division gene *ftsZ* (*PSPTO_4403*).	This study
AVL027	AGATGCTCAGGGCGTTTATC	Used with primer AVL028 for qRT-PCR of *Pst* DC3000 *hrpL* RNA polymerase sigma factor (*PSPTO_1404*).	Kvitko lab (University of Georgia)
AVL028	ACGCAGGGCTTCAAGAAA	Used with primer AVL027 for qRT-PCR of *Pst* DC3000 *hrpL* RNA polymerase sigma factor (*PSPTO_1404*).	Kvitko lab (University of Georgia)
AVL029	TGAAGGTCGGTGACAAGTTC	Used with primer AVL030 for qRT-PCR of *Pst* DC3000 gene *PSPTO_4337*, which encodes a pyruvate kinase involved in the Entner-Doudoroff pathway.	This study
AVL030	CCGCAATCCTTGACCAGAT	Used with primer AVL029 for qRT-PCR of *Pst* DC3000 gene *PSPTO_4337*, which encodes a pyruvate kinase involved in the Entner-Doudoroff pathway.	This study
AVL031	CAACTCGGGTGACGAAGATAAC	Used with primer AVL032 for qRT-PCR of *Pst* DC3000 housekeeping RNA polymerase sigma factor *rpoD* (*PSPTO_0537*).	This study
AVL032	GCTTTGAGGTGCTTGGTTACTA	Used with primer AVL031 for qRT-PCR of *Pst* DC3000 housekeeping RNA polymerase sigma factor *rpoD* (*PSPTO_0537*).	This study
AVL033	TACAACGCCATCCACGGCAA	Primer specific for *mCerulean3* that will not hybridize with the sequence of *mCitrine*. Used for sequencing.	This study
AVL034	GAACTTCAAGATCCGCCACA	Primer specific for *mCitrine* that will not hybridize with the sequence of *mCerulean3*. Used for sequencing.	This study

Table S13. Comparison of the three different methods used to determine differential gene expression in *Pseudomonas syringae* pv. *tomato* Δ*hrcC*Δ*CFA*.

Comparison	Unique to DEStringTie	Unique to DESalmon	Unique to Cuffdiff	DEStringTie and DESalmon	DEStringTie and Cuffdiff	DESalmon and Cuffdiff	Shared by all methods	Total DEGs
t_6 vs. t_{24}	90	158	18	177	9	5	58	425
t_6 vs. t_{168}	284	289	7	701	11	1	229	1,238
t_6 vs. Inoculum	148	388	224	343	118	76	1,505	2,654
t_6 vs. Logarithmic	205	184	220	336	160	67	1,684	2,651
t_6 vs. Stationary	171	192	203	258	162	58	1,247	2,120
t_{24} vs. t_{168}	275	285	9	674	6	2	146	1,122
t_{24} vs. Inoculum	203	269	125	436	91	46	1,311	2,278
t_{24} vs. Logarithmic	222	176	131	464	112	32	1,595	2,510
t_{24} vs. Stationary	215	189	111	378	113	45	1,106	1,942
t_{168} vs. Inoculum	292	468	14	985	17	18	926	2,428
t_{168} vs. Logarithmic	415	291	24	1,077	37	12	1,017	2,458
t_{168} vs. Stationary	286	320	23	870	34	12	624	1,883
Inoculum vs. Logarithmic	77	89	1,221	79	223	243	2,326	4,181
Inoculum vs. Stationary	57	196	1,308	50	155	259	1,548	3,516
Logarithmic vs. Stationary	38	34	2,077	18	354	150	1,434	4,067

Differential gene expression was determined by either of three methods: DESeq2 after pseudo-alignment with Salmon (DESalmon), DESeq2 after alignment with HISAT2 and analysis with StringTie (DEStringTie), and Cuffdiff after alignment with HISAT2 (Cuffdiff), when comparing the inoculum, 6 (t_6), 24 (t_{24}) and 168 (t_{168}) hours post-inoculation into Arabidopsis leaves, and *in vitro* cultures grown to logarithmic or stationary phase. The number of differentially expressed genes (false discovery rate adjusted *p*-value less than 0.05) unique or shared by these analyses is shown.

Table S14. Comparison of the three different methods used to determine differential gene expression in *Achromobacter xylosoxidans* Col-0-50.

Comparison	Unique to DEStringTie	Unique to DESalmon	Unique to Cuffdiff	DEStringTie and DESalmon	DEStringTie and Cuffdiff	DESalmon and Cuffdiff	Shared by all methods	Total DEGs
t_6 vs. t_{24}	55	34	67	65	18	7	128	319
t_6 vs. t_{168}	167	122	23	344	32	3	459	983
t_6 vs. Inoculum	46	32	567	145	153	36	938	1,871
t_{24} vs. t_{168}	145	74	10	252	19	6	216	577
t_{24} vs. Inoculum	71	57	382	227	140	37	996	1,839
t_{168} vs. Inoculum	147	105	132	404	68	23	909	1,641

Differential gene expression was determined by either of three methods: DESeq2 after pseudo-alignment with Salmon (DESalmon), DESeq2 after alignment with HISAT2 and analysis with StringTie (DEStringTie), and Cuffdiff after alignment with HISAT2 (Cuffdiff), when comparing the inoculum, 6 (t_6), 24 (t_{24}) and 168 (t_{168}) hours post-inoculation into Arabidopsis leaves. The number of differentially expressed genes (false discovery rate adjusted *p*-value less than 0.05) unique or shared by these analyses is shown.

Table S15. Comparison of the three different methods used to determine differential gene expression in *Pandoraea* sp. Col-0-28.

Comparison	Unique to DEStringTie	Unique to DESalmon	Unique to Cuffdiff	DEStringTie and DESalmon	DEStringTie and Cuffdiff	DESalmon and Cuffdiff	Shared by all methods	Total DEGs
t_6 vs. t_{24}	32	40	29	68	9	14	169	329
t_6 vs. t_{168}	157	131	26	260	40	13	513	983
t_6 vs. Inoculum	25	36	422	35	66	85	670	1,314
t_{24} vs. t_{168}	111	99	25	192	28	5	221	570
t_{24} vs. Inoculum	25	34	302	49	62	102	715	1,264
t_{168} vs. Inoculum	129	115	105	175	77	40	843	1,355

Differential gene expression was determined by either of three methods: DESeq2 after pseudo-alignment with Salmon (DESalmon), DESeq2 after alignment with HISAT2 and analysis with StringTie (DEStringTie), and Cuffdiff after alignment with HISAT2 (Cuffdiff), when comparing the inoculum, 6 (t_6), 24 (t_{24}) and 168 (t_{168}) hours post-inoculation into Arabidopsis leaves. The number of differentially expressed genes (false discovery rate adjusted *p*-value less than 0.05) unique or shared by these analyses is shown.

Dataset S1. Average gene expression in *Pseudomonas syringae* pv. *tomato ΔhrcCΔCFA*. (*A*) Average gene expression in *Pseudomonas syringae* pv. *tomato ΔhrcCΔCFA* calculated using DESeq2 after StringTie read alignment. Gene expression used the logarithm in base 2 fold-change values calculated by DESeq2 after aligning the reads with StringTie, when comparing the inoculum (KB), 6 (t_6), 24 (t_{24}) and 168 (t_{168}) hours post-inoculation into Arabidopsis leaves, and *in vitro* cultures grown to logarithmic or stationary phase. Each treatment had four biological replicates. *p*-values and false discovery rate adjusted *p*-values (*q*-values) calculated by DESeq2 for each comparison are shown. Extreme outliers and zero read counts causes DESeq2 to not perform statistical analyses and set the *p*-values to NA. When available, gene name for each locus is shown. DNA template strand, coordinates for the start and end of each gene, and a brief annotation are also shown. (*B*) Average gene expression in *Pseudomonas syringae* pv. *tomato* *ΔhrcCΔCFA* calculated using DESeq2 after Salmon read alignment. Gene expression used the logarithm in base 2 fold-change values calculated by DESeq2 after aligning the reads with Salmon, when comparing the inoculum (KB), 6 (t_6), 24 (t_{24}) and 168 (t_{168}) hours post-inoculation into Arabidopsis leaves, and *in vitro* cultures grown to logarithmic or stationary phase. Each treatment had four biological replicates. *p*-values and false discovery rate adjusted *p*-values (*q*-values) calculated by DESeq2 for each comparison are shown. Extreme outliers and zero read counts causes DESeq2 to not perform statistical analyses and set the *p*-values to NA. When available, gene name for each locus is shown. DNA template strand, coordinates for the start and end of each gene, and a brief annotation are also shown. (*C*) Average gene expression in *Pseudomonas syringae* pv. *tomato ΔhrcCΔCFA* calculated using Cuffdiff. Gene expression used the logarithm in base 2 fold-change values calculated by Cuffdiff, when comparing the inoculum (KB), 6 (t_6), 24 (t_{24}) and 168 (t_{168}) hours post-inoculation into Arabidopsis leaves, and *in vitro* cultures grown to logarithmic or stationary phase. Each treatment had four biological replicates. *p*-values and false discovery rate adjusted *p*-values (*q*-values) calculated by Cuffdiff for each comparison are shown. Zero read counts causes Cuffdiff to set the $\log_2$ fold-change values to –Inf (–) or Inf. When available, gene name for each locus is shown. DNA template strand, coordinates for the start and end of each gene, and a brief annotation are also shown.

Dataset S2. Average gene expression in *Achromobacter xylosoxidans* Col-0-50. (*A*) Average gene expression in *Achromobacter xylosoxidans* Col-0-50 calculated using DESeq2 after StringTie read alignment. Gene expression used the logarithm in base 2 fold-change values calculated by DESeq2 after aligning the reads with StringTie, when comparing the inoculum (LM), 6 (t_6), 24 (t_{24}) and 168 (t_{168}) hours post-inoculation into Arabidopsis leaves. Each treatment had four biological replicates. *p*-values and false discovery rate adjusted *p*-values (*q*-values) calculated by DESeq2

for each comparison are shown. Extreme outliers and zero read counts causes DESeq2 to not perform statistical analyses and set the *p*-values to NA. When available, gene name for each locus is shown. DNA template strand, coordinates for the start and end of each gene, and a brief annotation are also shown. (*B*) Average gene expression in *Achromobacter xylosoxidans* Col-0-50 calculated using DESeq2 after Salmon read alignment. Gene expression used the logarithm in base 2 fold-change values calculated by DESeq2 after aligning the reads with Salmon, when comparing the inoculum (LM), 6 (t_6), 24 (t_{24}) and 168 (t_{168}) hours post-inoculation into Arabidopsis leaves. Each treatment had four biological replicates. *p*-values and false discovery rate adjusted *p*-values (*q*-values) calculated by DESeq2 for each comparison are shown. Extreme outliers and zero read counts causes DESeq2 to not perform statistical analyses and set the *p*-values to NA. When available, gene name for each locus is shown. DNA template strand, coordinates for the start and end of each gene, and a brief annotation are also shown. (*C*) Average gene expression in *Achromobacter xylosoxidans* Col-0-50 calculated using Cuffdiff. Gene expression used the logarithm in base 2 fold-change values calculated by Cuffdiff, when comparing the inoculum (LM), 6 (t_6), 24 (t_{24}) and 168 (t_{168}) hours post-inoculation into Arabidopsis leaves. Each treatment had four biological replicates. *p*-values and false discovery rate adjusted *p*-values (*q*-values) calculated by Cuffdiff for each comparison are shown. Zero read counts causes Cuffdiff to set the $\log_2$ fold-change values to –Inf (–) or Inf. When available, gene name for each locus is shown. DNA template strand, coordinates for the start and end of each gene, and a brief annotation are also shown.

Dataset S3. Average gene expression in *Pandoraea* sp. Col-0-28. (*A*) Average gene expression in *Pandoraea* sp. Col-0-28 calculated using DESeq2 after StringTie read alignment. Gene expression used the logarithm in base 2 fold-change values calculated by DESeq2 after aligning the reads with StringTie, when comparing the inoculum (LM), 6 (t_6), 24 (t_{24}) and 168 (t_{168}) hours post-inoculation into Arabidopsis leaves. Each treatment had four biological replicates. *p*-values and false discovery rate adjusted *p*-values (*q*-values) calculated by DESeq2 for each comparison are shown. Extreme outliers and zero read counts causes DESeq2 to not perform statistical analyses and set the *p*-values to NA. When available, gene name for each locus is shown. DNA template strand, coordinates for the start and end of each gene, and a brief annotation are also shown. (*B*) Average gene expression in *Pandoraea* sp. Col-0-28 calculated using DESeq2 after Salmon read alignment. Gene expression used the logarithm in base 2 fold-change values calculated by DESeq2 after aligning the reads with Salmon, when comparing the inoculum (LM), 6 (t_6), 24 (t_{24}) and 168 (t_{168}) hours post-inoculation into Arabidopsis leaves. Each treatment had four biological replicates. *p*-values and false discovery rate adjusted *p*-values (*q*-values) calculated by DESeq2

for each comparison are shown. Extreme outliers and zero read counts causes DESeq2 to not perform statistical analyses and set the *p*-values to NA. When available, gene name for each locus is shown. DNA template strand, coordinates for the start and end of each gene, and a brief annotation are also shown. (*C*) Average gene expression in *Pandoraea* sp. Col-0-28 calculated using Cuffdiff. Gene expression used the logarithm in base 2 fold-change values calculated by Cuffdiff, when comparing the inoculum (LM), 6 (t_6), 24 (t_{24}) and 168 (t_{168}) hours post-inoculation into Arabidopsis leaves. Each treatment had four biological replicates. *p*-values and false discovery rate adjusted *p*-values (*q*-values) calculated by Cuffdiff for each comparison are shown. Zero read counts causes Cuffdiff to set the $\log_2$ fold-change values to –Inf (–) or Inf. When available, gene name for each locus is shown. DNA template strand, coordinates for the start and end of each gene, and a brief annotation are also shown.

SI REFERENCES

1. T. Nobori *et al.*, Transcriptome landscape of a bacterial pathogen under plant immunity. Proc. Natl. Acad. Sci. USA **115**, E3055–E3064 (2018).
2. Y.J. Chen *et al.*, Characterization of 582 natural and synthetic terminators and quantification of their design constraints. Nat. Methods **10**, 659–664 (2013).
3. A. Kamionka *et al.*, Induction of single chain tetracycline repressor requires the binding of two inducers. Nucleic Acids Res. **34**, 3834–3841 (2006).
4. O. Griesbeck, G.S. Baird, R.E. Campbell, D.A. Zacharias, R.Y. Tsien, Reducing the environmental sensitivity of yellow fluorescent protein. Mechanism and applications. J. Biol. Chem. **276**, 29188–29194 (2001).
5. K.H. Choi, H.P. Schweizer, mini-Tn7 insertion in bacteria with single attTn7 sites: example *Pseudomonas aeruginosa*. Nat. Protoc **1**, 153–161 (2006).
6. S. Zobel *et al.*, Tn7-based device for calibrated heterologous gene expression in *Pseudomonas putida*. ACS Synth. Biol. **4**, 1341–1351 (2015).
7. M.L. Markwardt *et al.*, An improved cerulean fluorescent protein with enhanced brightness and reduced reversible photoswitching. PLoS One **6**, e17896 (2011).
8. K.H. Choi *et al.*, Genetic tools for select-agent-compliant manipulation of *Burkholderia pseudomallei*. Appl. Environ. Microbiol. **74**, 1064–75 (2008).
9. B.H. Kvitko *et al.*, A simple method for construction of pir+ Enterobacterial hosts for maintenance of R6K replicon plasmids. BMC Res. Notes **5**, 157 (2012).
10. K.J. Livak, T.D. Schmittgen, Analysis of relative gene expression data using real-time quantitative PCR and the $2^{-\Delta\Delta C_T}$ method. Methods **25**, 402–408 (2001).
11. A. Smith, A.H. Lovelace, B.H. Kvitko, Validation of RT-qPCR approaches to monitor *Pseudomonas syringae* gene expression during infection and exposure to Pattern-triggered immunity. Mol. Plant Microbe Interact. **31**, 410–419 (2018).

12. A.H. Lovelace, A. Smith, B.H. Kvitko, Pattern-triggered immunity alters the transcriptional regulation of virulence-associated genes and induces the sulfur starvation response in *Pseudomonas syringae* pv. *tomato* DC3000. Mol. Plant Microbe Interact. **31**, 750–765 (2018).
13. A.C. Velásquez, M. Oney, B. Huot, S. Xu, S.Y. He, Diverse mechanisms of resistance to *Pseudomonas syringae* in a thousand natural accessions of *Arabidopsis thaliana*. New Phytol. **214**, 1673–1687 (2017).
14. A. C. Velásquez, S. Y. He, Data from "In planta transcriptome of phyllosphere endophytic bacteria." Sequence Read Archive. Available at https://www.ncbi.nlm.nih.gov/sra?linkname=bioproject_sra_all&from_uid=738276. Deposited 16 June 2021.
15. S. Andrews, FastQC: A Quality Control Tool for High Throughput Sequence Data. (2010). http://www.bioinformatics.babraham.ac.uk/projects/fastqc/.
16. M. Martin, Cutadapt removes adapter sequences from high-throughput sequencing reads. EMBnet.j. **17**, 10–12 (2011).
17. C. Trapnell *et al.*, Differential analysis of gene regulation at transcript resolution with RNA-seq. Nat. Biotechnol. **31**, 46–53 (2013).
18. D. Kim, J.M. Paggi, C. Park, C. Bennett, S.L. Salzberg, Graph-based genome alignment and genotyping with HISAT2 and HISAT-genotype. Nat. Biotechnol. **37**, 907–915 (2019).
19. H. Li *et al.*, The Sequence Alignment/Map format and SAMtools. Bioinformatics **25**, 2078–2079 (2009).
20. S. Kovaka *et al.*, Transcriptome assembly from long-read RNA-seq alignments with StringTie2. Genome Biol. **20**, 278 (2019).
21. M.I. Love, W. Huber, S. Anders, Moderated estimation of fold change and dispersion for RNA-seq data with DESeq2. Genome Biol. **15**, 550 (2014).
22. R. Patro, G. Duggal, M.I. Love, R.A. Irizarry, C. Kingsford, Salmon provides fast and bias-aware quantification of transcript expression. Nat. Methods **14**, 417–419 (2017).
23. S. Maere, K. Heymans, M. Kuiper, BiNGO: a Cytoscape plugin to assess overrepresentation of gene ontology categories in biological networks. Bioinformatics **21**, 3448–3449 (2005).
24. F. Supek, M. Bošnjak, N. Škunca, T. Šmuc, REVIGO summarizes and visualizes long lists of gene ontology terms. PLoS One **6**, e21800 (2011).
25. R. Hernández-de-Diego *et al.*, PaintOmics 3: a web resource for the pathway analysis and visualization of multi-omics data. Nucleic Acids Res. **46**, W503–W509 (2018).
26. K. Blin *et al.*, antiSMASH 5.0: updates to the secondary metabolite genome mining pipeline. Nucleic Acids Res. **47**, W81–W87 (2019).
27. J. Ernst, Z. Bar-Joseph, STEM: a tool for the analysis of short time series gene expression data. BMC Bioinformatics **7**, 191 (2006).
28. L. Li, C.J. Stoeckert, Jr., D.S. Roos, OrthoMCL: identification of ortholog groups for eukaryotic genomes. Genome Res. **13**, 2178–2189 (2003).
29. A. Levy *et al.*, Genomic features of bacterial adaptation to plants. Nat. Genet. **50**, 138–150 (2018).
30. L. Fu, B. Niu, Z. Zhu, S. Wu, W. Li, CD-HIT: accelerated for clustering the next-generation sequencing data. Bioinformatics **28**, 3150–3152 (2012).
31. C. Chen *et al.*, TBtools: An integrative toolkit developed for interactive analyses of big biological data. Mol. Plant **13**, 1194–1202 (2020).
32. T. Chen *et al.*, A plant genetic network for preventing dysbiosis in the phyllosphere. Nature **580**, 653-657 (2020).
33. D. Hanahan, "Techniques for transformation of *E. coli*" in DNA cloning. A practical approach. Volume 1, D.M. Glover, Ed. (IRL Press, Oxford-Washington, DC, 1985), pp. 109–135.

34. D.A. Cuppels, Generation and characterization of Tn5 insertion mutations in *Pseudomonas syringae* pv. *tomato*. Appl. Environ. Microbiol. **51**, 323–327 (1986).
35. N. Puri *et al.*, Expression of *avrPphB*, an avirulence gene from *Pseudomonas syringae* pv. *phaseolicola*, and the delivery of signals causing the hypersensitive reaction in bean. Mol. Plant Microbe Interact. **10**, 247–256 (1997).
36. M.B. Mudgett, B.J. Staskawicz, Characterization of the *Pseudomonas syringae* pv. *tomato* AvrRpt2 protein: demonstration of secretion and processing during bacterial pathogenesis. Mol. Microbiol. **32**, 927–941 (1999).
37. M. Hinsch, B. Staskawicz, Identification of a new *Arabidopsis* disease resistance locus, *RPS4*, and cloning of the corresponding avirulence gene, *avrRps4*, from *Pseudomonas syringae* pv. *pisi*. Mol. Plant Microbe Interact. **9**, 55–61 (1996).
38. J.N. Worley *et al.*, *Pseudomonas syringae* pv. *tomato* DC3000 CmaL (*PSPTO4723*), a DUF1330 family member, is needed to produce L-allo-isoleucine, a precursor for the phytotoxin coronatine. J. Bacteriol. **195**, 287–96 (2013).
39. N. Bodenhausen, M. Bortfeld-Miller, M. Ackermann, J.A. Vorholt, A synthetic community approach reveals plant genotypes affecting the phyllosphere microbiota. PLoS Genet. **10**, e1004283 (2014).
40. X.F. Xin *et al.*, Bacteria establish an aqueous living space in plants crucial for virulence. Nature **539**, 524–529 (2016).
41. K. Berendzen *et al.*, A rapid and versatile combined DNA/RNA extraction protocol and its application to the analysis of a novel DNA marker set polymorphic between *Arabidopsis thaliana* ecotypes Col-0 and Landsberg *erecta*. Plant Methods **1**, 4 (2005).
42. R. González-Lamothe, M. El Oirdi, N. Brisson, K. Bouarab, The conjugated auxin indole-3-acetic acid-aspartic acid promotes plant disease development. Plant Cell **24**, 762–777 (2012).
